# Volatiles from the Mandibular Gland Reservoir Content of *Colobopsis explodens* Laciny and Zettel, 2018, Worker Ants (Hymenoptera: Formicidae)

**DOI:** 10.3390/molecules24193468

**Published:** 2019-09-24

**Authors:** Michaela Hoenigsberger, Alexey G Kopchinskiy, Christoph Bueschl, Alexandra Parich, Alice Laciny, Herbert Zettel, Kamariah A Salim, Linda BL Lim, Irina S Druzhinina, Rainer Schuhmacher

**Affiliations:** 1 Institute of Bioanalytics and Agro-Metabolomics (iBAM), Department of Agrobiotechnology (IFA-Tulln), University of Natural Resources and Life Sciences, Vienna (BOKU), Konrad-Lorenz-Strasse 20, A-3430 Tulln, Austria; michaela.fischer@boku.ac.at (M.H.); christoph.bueschl@boku.ac.at (C.B.); alexandra.parich@boku.ac.at (A.P.); 2 Institute of Chemical, Environmental and Bioscience Engineering (ICEBE), TU Wien, Gumpendorferstrasse 1a, A-1060 Vienna, Austria; alexey.kopchinskiy@tuwien.ac.at (A.G.K.); or irina.druzhinina@njau.edu.cn (I.S.D.); 3 2nd Zoological Department, Natural History Museum Vienna, Burgring 7, A-1010 Vienna, Austria; alice.laciny@nhm-wien.ac.at (A.L.); herbert.zettel@nhm-wien.ac.at (H.Z.); 4 Environmental and Life Sciences, Faculty of Science, Universiti Brunei Darussalam, Jalan Tungku Gadong BE1410, Brunei Darussalam; udhl_2003@yahoo.com; 5 Chemical Sciences, Faculty of Science, Universiti Brunei Darussalam, Jalan Tungku Link, Gadong BE1410, Brunei Darussalam; linda.lim@ubd.edu.bn; 6 Fungal Genomics Group, College of Resources and Environmental Sciences, Nanjing Agricultural University, Weigang NO. 1, Nanjing 210095, China

**Keywords:** *Colobopsis cylindrica* species group, headspace solid-phase microextraction (HS-SPME), mandibular gland, metabolomics, phenolics, phloroglucinols

## Abstract

Forty-five volatile organic compounds (VOCs) were identified or annotated in the mandibular gland reservoir content (MGRC) of the Southeast Asian ant *Colobopsis explodens* Laciny and Zettel, 2018 (Hymenoptera: Formicidae), using headspace solid-phase microextraction (HS-SPME) coupled to gas chromatography-mass spectrometry (GC-MS) and liquid extraction combined with GC-MS. In extension of previous reports on VOCs of *C. explodens*, members of different compound classes, such as alkanes, aliphatic and aromatic carboxylic acids, and phenolics, were detected. The ketone 2-heptanone and the biochemically related phenolics benzene-1,3,5-triol (phloroglucinol, PG), 1-(2,4,6-trihydroxyphenyl)ethanone (monoacetylphloroglucinol, MAPG), 5,7-dihydroxy-2-methylchromen-4-one (noreugenin), and 1-(3-Acetyl-2,4,6-trihydroxyphenyl)ethanone (2,4-diacetylphloroglucinol, DAPG) dominated the GC-MS chromatograms. The identities of the main phenolics MAPG and noreugenin were further verified by liquid chromatography-high resolution-tandem mass spectrometry (LC-HRMS/MS). A comparative study of MGRC samples originating from three distinct field expeditions revealed differences in the VOC profiles, but the presence and relative abundances of the dominating constituents were largely consistent in all samples. Our study considerably extends the knowledge about the number and type of VOCs occurring in the MGRC of *C. explodens*. Based on the type of the detected compounds, we propose that the likely irritant and antibiotic phenolic constituents play a role in defense against arthropod opponents or in protection against microbial pathogens.

## 1. Introduction

According to the World Health Organization (WHO), volatile organic compounds (VOCs) are small molecules with high vapor pressure at room-temperature and boiling points between 50 and approximately 260 °C at atmospheric pressure [1]. A large diversity of VOCs can be produced by eukaryotic plants, insects, and fungi as well as by prokaryotes [2,3,4,5,6]. Since VOCs mediate interactions among organisms, they have attracted great attention in pharmacy, agriculture, and in the food and perfumery industries [7,8,9,10,11].

VOCs present in secretions of insects have diverse roles, especially as pheromones and defense compounds [12,13,14]. In the family Formicidae, a wide range of VOCs, such as terpenes, formic acid or alkanes has been described to be utilized as alarm pheromones [14,15,16]. It is likely that such metabolites were originally used as defense compounds, before they have become relevant for communication between organisms [14,17]. Many alarm- and defense-related VOCs are known and have been found in diverse ant species [14,18,19,20,21]. However, for the vast majority of VOCs, their function(s) has (have) not been elucidated so far [22]. This also holds true for the compounds found in the special defensive secretion of *Colobopsis explodens* Laciny and Zettel, 2018, investigated in this study.

*Colobopsis explodens* belongs to the *Colobopsis cylindrica* (COCY) species group (Formicidae: Hymenoptera) and has recently been described in detail as a model species of Borneo’s “exploding ants” [23]. Within the COCY group, *C. explodens* can be recognized by its chiefly reddish-brown color, relatively short appendages, and the bright yellow mandibular gland secretion of minor workers [23]. Females of *C. explodens* can be assigned to three morphologically distinct castes. Queens (gynes) measure over 1 cm in body length, possess plug-shaped heads and are adapted for flight and reproduction. The slightly smaller major workers (“doorkeepers”) resemble the queens in head-shape and use these heads to plug the entrance holes of wooden nests against intruders (phragmosis) [23]. Minor workers exhibit a considerable size range of 4-7 mm and possess hypertrophied mandibular glands (MGs) the reservoirs of which extend through almost the ants’ whole bodies [23,24,25]. In a typical one-to-one confrontation with another arthropod, a *C. explodens* minor worker can suicidally rupture its gastral integument to release the sticky mandibular gland reservoir content (MGRC) onto the body of the opponent, which is subsequently detained by the glue-like secretion and usually dies after the attack [24,26,27,28]. Although the composition of VOCs of different COCY species has been investigated, the knowledge about their chemical structures is still scarce. In the few studies investigating the composition of the MGRC of COCY ants, mostly aliphatic and phenolic compounds have been reported [27,29,30,31,32]. Jones et al. analyzed the MG products in a small collection of whole specimen of already “exploded” *C. explodens* minor workers by gas chromatography-mass spectrometry (GC-MS) and reported the aliphatics *n*-undecane, *n*-heptadecane, tetradecan-1-ol, hexadecan-1-ol, octadecan-1-ol, heptan-2-one and methyl-3,5-dioxohexanoate plus the phenolic compounds 1-(2,4,6-trihydroxyphenyl)ethanone (monoacetylphloroglucinol, MAPG), 5,7-dihydroxy-2-methylchromen-4-one (noreugenin) and 1-(2,4-dihydroxyphenyl)ethanone (2,4-dihydroxyacetophenone, DHAP) [30] (*C. explodens* sp.n. herein designated as *Camponotus* (*cylindricus* group) sp. “02-108”).

Under both natural and laboratory conditions, it has frequently been observed that the MGRC constituents are used in defense against other ants [25,29,30]. Based on the assumption that the MGRC of *C. explodens* plays a crucial role for survival of this ant species, the objective of this study was to elucidate the VOCs present in the MGRC of *C. explodens* minor workers. For this purpose, a large number of ants was collected during three different sampling expeditions to Brunei. Individual specimens were dissected, and the VOCs contained in their MGRC were analyzed by GC-MS and LC-HRMS/MS.

Our study demonstrates that *C. explodens* accumulates a remarkable number of phenolic compounds in its mandibular gland reservoirs (MGRs) which may be of relevance for the defense against other insects or for protection against microbial pathogens. Interestingly, some of these compounds are only known to be produced by microorganisms so far.

## 2. Results

### 2.1. Identification and Annotation of VOCs in the MGRCs of *C. explodens* Minor Workers

**Figure 1:**
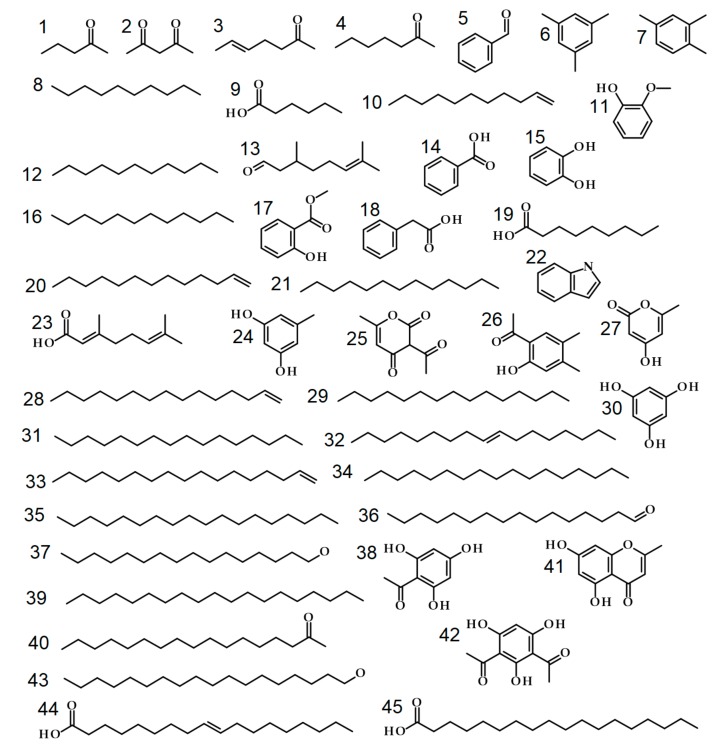
Structures of compounds identified or annotated in the MGRC of *C. explodens* minor workers. Numbering was chosen according to Table 1.

HS-SPME-GC-MS and liquid-injection GC-MS measurements of MGRC samples originating from a total of 380 *C. explodens* minor worker ants (Table S1) resulted in the detection of 151 and 275 compounds, respectively, with an overlap between the two methods of 102 VOCs. Forty VOCs were identified, and five were annotated based on comparison of their retention indices (RI) and mass spectra with authentic reference compounds or GC-MS spectral libraries (Table 1, compound structures shown in Figure 1). It is worth mentioning that the unambiguous identification of all biochemical constituents is a major challenge in untargeted metabolomics approaches and is a matter of current debate. For annotation of chemical structures, different levels of confidence have been suggested by the Metabolomics Standards Initiative (MIS) [33,34,35]. According to the MSI, compound identification (confidence level 1) requires data from at least two orthogonal techniques such as retention index (RI) and mass spectrum, which are confirmed with an authentic reference standard under the same conditions. In absence of a reference standard, level 2 can be achieved in case of a single remaining candidate or level 3 if RI and mass spectrum match but still allow more than one structure candidate. Here, identification of the detected VOCs was achieved by assignment of spectrum similarity factor and Kovats RI value and their combination to an overall similarity score (OSS) with the help of the MetaboliteDetector software [36]. Based on these criteria and the MSI classification system, 40 VOCs were identified (level 1), one was annotated according to level 2, and four were annotated according to level 3 (Table 1). A detailed list of the achieved scores and corresponding identification/annotation criteria are given in Table S2. For the sake of completeness, a comparison of library and experimental spectra of all 45 identified or annotated compounds are compiled together with their assigned structure formula and chemical identifier (Supplementary File Compounds.pdf). Most of the identified or annotated compounds of our study have been reported for ants or other insects before, as illustrated and exemplified in Table S3.

**Figure 2:**
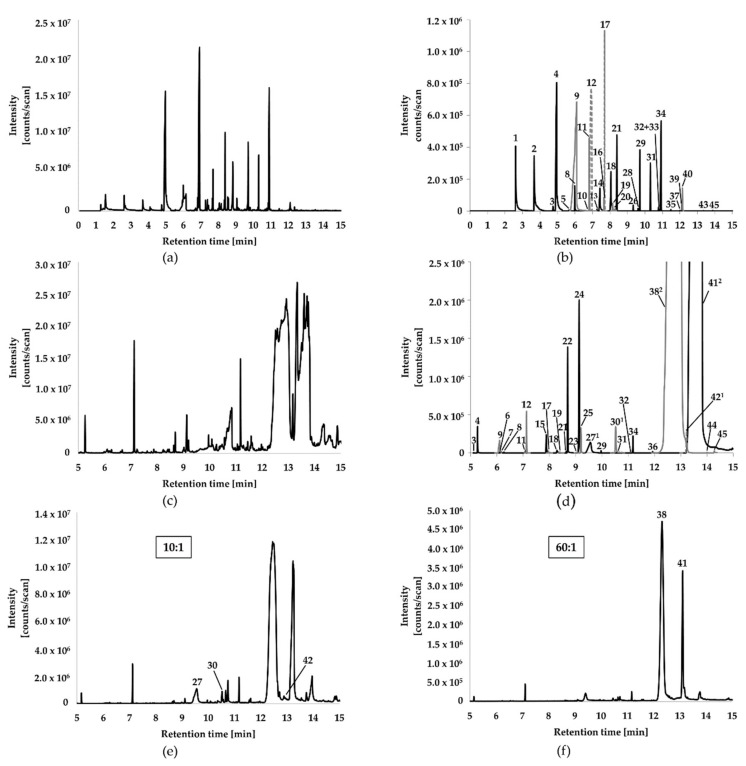
Total ion current (TIC) chromatogram and overlaid extracted ion chromatograms (EIC) of VOCs identified or annotated in the MGRC extracts of *C. explodens*. (**a**) TIC chromatogram obtained after headspace solid-phase microextraction-gas chromatography-mass spectrometry (HS-SPME-GC-MS) measurement. (**b**) Overlay of EICs obtained after HS-SPME-GC-MS. (**c**) TIC chromatogram obtained after injection of EtOAc extracts into the gas chromatography-mass spectrometry (GC-MS) instrument. (**d**) Zoomed overlay of EICs obtained after injection of the extracts in splitless mode. ^1^ Compounds identified after choosing a GC split ratio of 10:1; ^2^ Compounds identified after choosing a GC split ratio of 60:1. The intensities at the peak maxima of compounds 38 and 41 were 4.5 × 10^6^ and 8 × 10^6^, respectively. (**e**) TIC chromatogram obtained after choosing a GC split ratio of 10:1, necessary for identification of compounds with IDs 27, 30, and 42 after liquid-injection GC-MS. (**f**) TIC chromatogram obtained after choosing a GC split ratio of 60:1, necessary for identification of compounds with IDs 38 and 41 after liquid-injection GC-MS. Grey peaks indicate partly coelution of different compounds. For their identification or annotation, spectra deconvolution was inevitable. Peak numbers correspond to IDs given in Table 1.

The identified compounds covered diverse substance classes including alkanes, alkenes, ketones, aliphatic and aromatic carboxylic acids, and phenolics. Total ion current (TIC) chromatograms and an overlay of extracted-ion chromatograms (EICs) of the identified or annotated VOCs are shown in Figure 2. Since the constituents with IDs 27, 30, 38, 41, and 42 caused column overloading and exhibited distorted peak shapes after injection of liquid extracts into the GC-MS instrument, an additional “dilution” of extracts by increasing the split ratio to 10:1 (in case of compounds with ID 27, 30, and 42) or to 60:1 (for compounds with IDs 38 and 41) was necessary for proper RI and spectra evaluation (Figure 2c–f).

### 2.2. LC-HRMS/MS for Confirmation of MAPG and Noreugenin.

**Figure 3:**
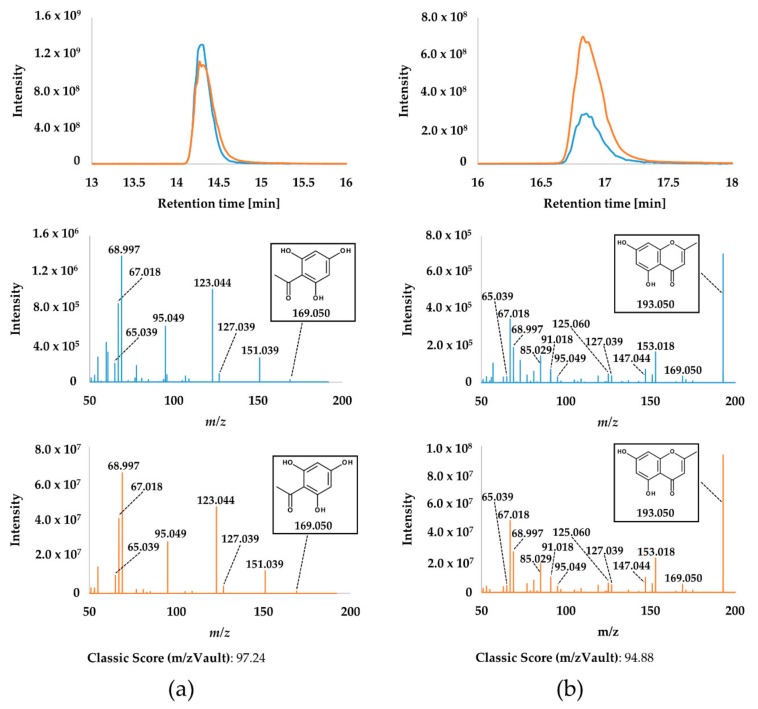
Liquid chromatography-high resolution-tandem mass spectrometry (LC-HRMS/MS) chromatograms and product ion spectra of monoacetylphloroglucinol (MAPG) (**a**) and noreugenin (**b**) for pure standard compounds (blue) and Cexpl_5/2014_1 MGRC samples (orange). For both compound mass spectra overlap, the classic score calculated by mzVault, is given. Chromatographic intensity values for the standard compounds were multiplied by factor 50 for better illustration.

For confirmation of compounds MAPG and noreugenin, additional LC-HRMS/MS measurements were carried out to compare authentic standards with selected biological samples. While LC-HRMS enabled the verification of retention time and molecular mass of the protonated molecules (relative mass deviation between 0.7 and 1.4 ppm), both structures were successfully confirmed by LC-HRMS/MS (Figure 3). In all analyzed MGRC extracts, the identities of MAPG and noreugenin were verified in positive and in negative ionization mode.

### 2.3. Statistical Analysis

**Figure 4:**
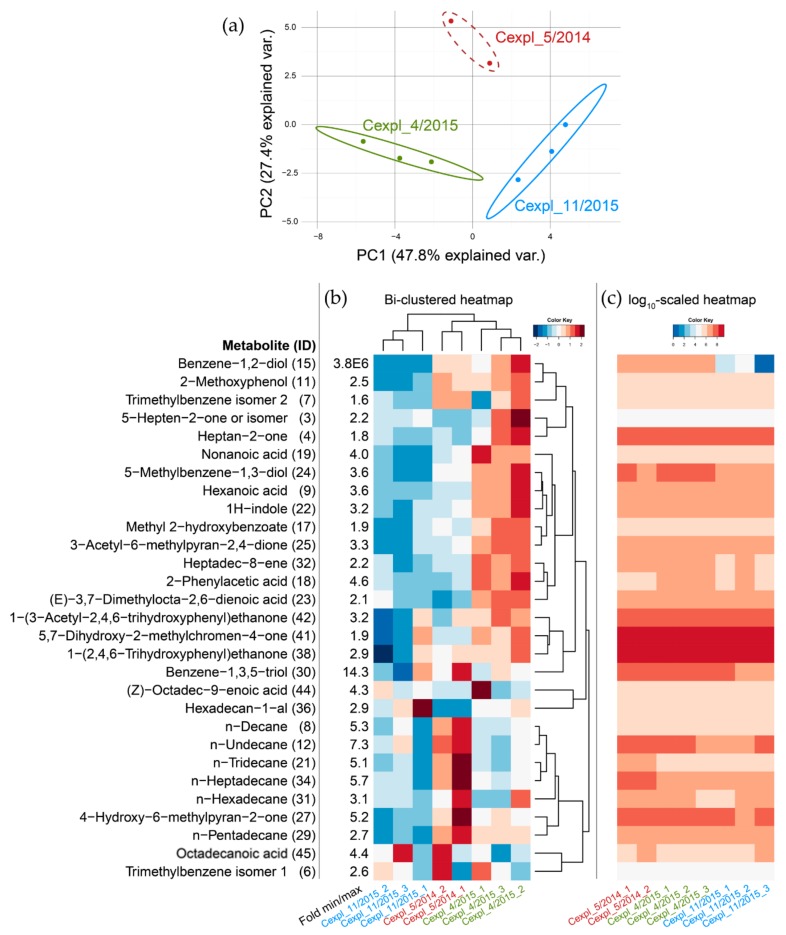
Statistical analysis based on GC-MS abundances (*i.e.*, EIC peak areas) of identified or annotated compounds in the MGRC of *C. explodens* after extraction with EtOAc. (**a**) PCA scores plot. Each dot represents a pooled MGRC sample. The X and Y axis show the principal components 1 (PC1) and 2 (PC2) that explain 47.8% and 27.4% of the total variance, respectively. Dashed circle around sample group “Cexpl_5/2014” was added for better illustration. (**b**) Two-way joining cluster analysis including sample- and compound-dendrograms. A color gradient, showing increased (red) and decreased (blue) abundance values relative to the average value of all samples, was chosen. The IDs given in brackets next to the compound names correspond to the numbering in Table 1. The column Fold min/max contains the ratios of largest to smallest EIC peak area as calculated for every listed compound. (**c**) Log10-scaled heatmap of measured EIC peak areas. For *m/z* values of EICs used to calculate the peak areas, see Table 1. The color code refers to arithmetic mean values of replicates and corresponds to compound levels in the measured experimental samples using a logarithmic (log10) color scale. Columns are ordered according to sample numbers and time-points.

Based on the GC-MS (liquid extraction, split/splitless injection)-derived EIC profiles, a data matrix consisting of all identified or annotated VOCs and their EIC peak areas obtained for all individual samples was established and subjected to statistical analysis (Table S4). Principal component analysis (PCA) and two-way joining cluster analysis resulted in a separation of the samples according to the sampling time points (Figure 4a,b). A group of MGR constituents (IDs 8, 12, 21, 27, 29, 31, and 34) was more abundant in *C. explodens* minor workers sampled in May 2014 (Cexpl_5/2014) compared to the ones sampled during the two later expeditions in 2015 (Figure 4b). Thereof all except the polyketide with the ID 27 constitute *n*-alkanes with chain lengths between 10 and 17 carbon atoms. In contrast, another major cluster of compounds (IDs 3, 4, 7, 9, 11, 15, 17, 18, 19, 22, 23, 24, 25, 32, 38, 41, and 42) was contained in higher amounts in the samples collected in April 2015 (Cexpl_4/2015) compared to the other two sample groups. Among those, the VOCs with the IDs 9, 14, 18, 19, 23, 25, 44, and 45 constitute carboxylic acids while compounds with the IDs 11, 15, 24, 38, 41, and 42 are phenolics. Further analysis revealed that, despite the clustering of samples into the different sampling time points (Figure 4a,b), the presence and the relative abundances of the constituents within individual VOC profiles were largely consistent over all samples and independent of the sampling time points. Interestingly, the phenolics MAPG (ID 38) and noreugenin (ID 41) dominated the VOC profiles in the MGRC collected irrespective of the sample or sampling time point. Moreover, the biochemically and structurally related compounds with the IDs 27, 30, and 42 were also consistently among the most abundant VOCs in the MGRC of the tested insects (Figure 4c).

## 3. Discussion

Secretions of insects can have their defensive potential via (semi-)volatile constituents. Consequently, GC-MS was the method of choice to elucidate putative bioactive metabolites contained in the MGRC of *C. explodens* [40]. Dissection of 380 ants for their MGRCs allowed the analysis of a relatively large number of biological samples. Since the samples used in this work originated from a single *C. explodens* colony, the number of collectable individuals was kept as small as possible to avoid destructive sampling and thus a break-down of the colony. HS-SPME and liquid extraction/injection have been used as complementary extraction/injection methods in this study. Liquid extraction and injection of extracts into a split-/splitless injector was used since this approach resulted in the detection of a higher number of compounds, especially of the higher-boiling point phenols, which otherwise would have been missed by SPME. Additionally, this method is suited for the comparative quantification of VOCs. The extraction step is less prone to matrix effects or discrimination of compounds compared to HS-SPME, which is known to be highly dependent on the matrix composition [41]. However, SPME as a complementary extraction technique provides added value as it enables the detection of low boiling/early eluting constituents. Compounds with IDs 1 and 2, for example, cannot be detected in the EtOAc extracts because of the broad solvent peak dominating the early GC-MS chromatogram. Thus, these two compounds were only detected after application of HS-SPME. Overall, six metabolites passed the strict criteria applied for identification and annotation after HS-SPME but not after liquid extraction, while another nine VOCs were exclusively assigned in the EtOAc extracts but not after HS-SPME.

In total, 45 VOCs were identified (confidence level 1 [35]) or annotated according to confidence level 2 or 3 [35] in this study (Table 1). All of these compounds can be assigned to the mandibular gland since dissected MGRC samples were used throughout the study. Seven of the 45 VOCs (IDs 4, 12, 34, 37, 38, 41, and 43) have already been reported for *C. explodens* before [30], while the remaining 38 volatile constituents (IDs 1–3, 5–11, 13–33, 35, 36, 39, 40, 42, 44, and 45) are identified or annotated in this species here for the first time. Three of the previously reported compounds, namely 1-(2,4-dihydroxyphenyl)ethanone, tetradecan-1-ol and methyl-3,5-dioxohexanoate were not detected in the MGRCs. EIC peaks for the first two were close to the limit of detection, thus the data was not suited for tentative structure identification. The third compound, methyl-3,5-dioxohexanoate, is the methyl ester of 3,5-dioxo-hexanoic acid and may had been formed by esterification with methanol, which had been used as solvent in the aforementioned study [30]. In order to exclude formation of methyl derivates from true biochemical constituents by reaction with the extraction solvent, ethyl acetate instead of methanol was used to extract the MGRC constituents. This may be the reason, why methyl-3,5-dioxohexanoate was not detected in our study [42,43]. Instead, the lactone of 3,5-dioxohexanoic acid 4-hydroxy-6-methylpyran-2-one (ID 27) was identified among the measured VOCs. This compound has been described to be formed as a polyketide from acetyl-CoA via two subsequent condensations with malonyl-CoA by different microbes, whereby the resulting intermediate (3,5-diketohexanoate thioester) undergoes ring closure to produce 4-hydroxy-6-methylpyran-2-one (ID 27) [44].

To compare the VOC profiles obtained for the samples of the different field expeditions, principal component analysis (PCA) and hierarchical clustering analysis (HCA) was carried out. With both unsupervised methods a separation of the samples according to the sampling time points was observed (Figure 4). The MGRCs of *C. explodens* minor workers sampled in 2014 revealed the highest abundances for the compounds with the IDs 8, 12, 21, 27, 29, 31, and 34 compared to the other two sampling times. This cluster contains all alkane compounds (IDs 8, 12, 21, 29, 31, and 34) identified after liquid-injection GC-MS. In general, hydrocarbons are often detected in exocrine secretions of insects and act as semiochemicals with various functions like sex pheromone activity or nest-mate recognition signals [2,16,21]. Moreover, shorter-chained alkanes, which were also identified in the MGRC of *C. explodens* here, show higher volatility and have been associated with alarm- and defense pheromone function or to act as wetting- and spreading agents for other, polar compounds such as formic acid [45,46,47]. Thus, the elevated abundance of alkanes in the ants collected in May 2014 is presumably related to the level of stress experienced by the living individuals during sampling. In the sample group collected in April 2015, the abundances of the carboxylic acids (IDs 9, 14, 18, 19, 23, 25, 44, and 45) and phenolics (IDs 11, 15, 24, 38, 41, and 42) revealed to be higher compared to the remaining sampling groups. Both compound classes comprise members with reported corrosive and irritant properties, which may have an effect in defense against arthropod enemies [30,48]. For some of them, also antimicrobial activity has been shown ([49,50]. An interpretation of differing abundance levels of the monitored VOCs is not straight forward. It has to be considered that the ants have been collected from their natural habitat under real-world conditions. Many unknown environmental factors (biotic and abiotic) may well have affected the abundance of some of the detected VOCs. Without a detailed knowledge or control of biotic as well as abiotic conditions an interpretation of the differences in compound abundances between the different sample groups is therefore not possible.

However, despite these slightly differing VOC profiles across sampling time points, the ketone with the ID 4 and the phenolics with the IDs 27, 30, 38, 41, and 42 consistently dominated the GC-MS profiles. 2-Heptanone (ID 4) is a suggested alarm pheromone from grass-cutting ants, but is also reported from honeybees that use the compound to paralyze small pests [51,52,53]. MAPG (ID 38) can be produced by an ectosymbiotic fungus of the ambrosia beetle, *Scolytoplatypus mikado* Blandford, 1893 [54]. The pure culture broth showed antimicrobial activity against some Gram-positive bacteria. After MS and nuclear magnetic resonance (NMR) analysis, the active components were revealed to be DAPG (ID 42) and MAPG (ID 38), besides PG (ID 30) [54]. DAPG (ID 42) was also extracted from cultured broth of a bacterial symbiont of the white-backed planthopper, *Sogatella furcifera* Horváth, 1899 which showed antibacterial activity against *Pseudomonas syringae* pv. *mori* and *Corynebacterium michiganense* pv. *michiganense* [54]. MAPG (ID 38) and noreugenin (ID 41) revealed to be the most dominant constituents in the MGRC of *C. explodens* analyzed in this study. Both have been identified in this species before and are thought to be responsible for the color of the MGRC, which can be seen through the tergites, when living *C. explodens* workers show their raised gasters during foraging [30]. The polyketides with the IDs 27, 30, 38, and 42 can also be produced by polyketide synthase enzymes via similar metabolic pathways as has been shown in Pseudomonads [55,56]. The bacteria derived and antimicrobial DAPG (ID 42) is used in the biological control of plant pathogens, e.g., in the control of take-all disease of wheat [57]. Pseudomonads or close relatives thereof are known symbionts in ants and may serve nitrogen-recycling or promote disease control in insects [27,55,58,59]. It is noteworthy that, next to the main phenolic constituents MAPG and noreugenin together with its biochemically related compounds, a number of other phenolic compounds were identified in the scope of our study. 2-Methoxyphenol (ID 11) is a known aggregation pheromone released from the feces of desert locusts *Schistocerca gregaria* Forsskål, 1775. In this species, the compound has been reported to be produced by gut bacteria and not by the insects themselves [60]. 2-Methoxyphenol (ID 11) is also known from the cyanogenic defensive secretions of polydesmid millipedes, in which the phenol arises from the arthropod′s own metabolism and not due to symbiotic bacteria [61]. The constituent 5-methylbenzene-1,3-diol (ID 24), reported from diverse COCY species, has been known from plant, fungal and lichen sources and also shows antibacterial and anti-phytopathogenic properties [29,30,62]. In general, phenols can be detected widely among insects, such as in Coleoptera, Orthoptera, Isoptera, and Blattodea, and provide protection against predators and pathogens [2]. Compounds derived from phloroglucinol (ID 30) possess broad spectrum antiviral, antibacterial, antifungal, antihelminithic, and phytotoxic properties and may therefore be utilized in protection against pathogens by the ants [55]. The likely antibiotic MGR mixture may be applied onto the ants’ bodies by self- or allo-grooming, thus ensuring a continuous antimicrobial shield on the cuticle. When the MGRC is ejected after the suicidal rupture of *C. explodens*, the secretion may also act against pathogens introduced by the arthropod opponents approaching the nest, thus protecting the colony and its microbiome. A recent study showed that the MG content obtained from individuals of an ant species belonging to the COCY species group inhibits spore germination of the fungi *Ophiocordyceps polyrhachis-furcata* and *Beauveria bassiana*, which points to a potential antimicrobial activity of the MGR constituents found in COCY ants [32] (ant species herein identified as “*Colobopsis saundersi* Emery, 1889”). Interestingly, GC-MS analysis of the MG content revealed phenolics (as MAPG) to be the major constituents in this species.

Our study demonstrates that the MGRC of *C. explodens* contains a number of potentially irritant compounds that may be utilized in the defense against arthropod opponents. Since some of the MGRC constituents have reported antimicrobial activity, they may play a role in the modulation of the microbiome of these ants, which is beyond the initially assumed role in defense against arthropods. Due to the polyketide origin of the dominant compounds we propose their potential production by microbial symbionts, thus one has to be aware that the detected constituents might not be synthesized de novo by the ants [63].

More research needs to be done to address i) the biosynthesis of the compounds detected in *C. explodens*, ii) the proposed antibacterial and antifungal properties of the MGRC and its main constituents, and iii) the role of the MGRC compounds in defense against other arthropods.

## 4. Materials and Methods

### 4.1. Ants

Minor workers of C. *explodens* were collected with aspirators in the lowland tropical rain forest surrounding the Kuala Belalong Field Studies Center (KBFSC) located in the Temburong District of Brunei Darussalam (4°32′48.2′′N 115°09′27.9′′E; for species identification see [23]). Ants were killed by freezing them rapidly at −20 °C and transported on dry ice to a −80 °C freezer. Frozen ants were transported on dry ice to Austria and kept at −80 °C until further analysis. Members of the *C. explodens* model colony to be analyzed by GC-MS were sampled in the course of three field expeditions, as shown in Table S1. To obtain the MGRCs from *C. explodens*, minor worker ants were dissected under cooled conditions as described in detail in Hoenigsberger et al. [37].

### 4.2. Chemical Analysis of MGRC VOCs

#### 4.2.1. Chemicals and Reagents

Ethyl acetate (EtOAc, ≥99.8% purity) was obtained from Carl Roth GmbH + Co. KG (Karlsruhe, Germany). Acetonitrile (ACN, HPLC-grade) and benzoic acid (≥99%) were purchased from VWR (Vienna, Austria). Water was purified successively by reverse osmosis and an ELGA Purelab Ultra-AN-MK2 system from Veolia Water (Vienna, Austria). Hexanoic acid (98%), 2-methoxyphenol (98%), hexadecan-1-al (>97%), undec-1-ene (99.5%, analytical standard), hexadecan-1-ol (>98%), 5-methylbenzene-1,3-diol (>98%), pentane-2,4-dione (>99%), tridec-1-ene (>95%), 1-(2,4,6-trihydroxyphenyl)ethanone (MAPG, >98%), octadecan-1-ol (>98%), pentadec-1-ene (>99.0%, analytical standard), 1,2,4-trimethylbenzene (>98%), benzene-1,2-diol (≥99%) and 1,3,5- trimethylbenzene (>97%) were bought from TCI Europe N.V. (Eschborn, Germany). 3-Acetyl-6-methylpyran-2,4-dione (98%) and 1-(2-hydroxy-4,5-dimethylphenyl)ethanone (98%) were obtained from Alfa Aesar (now: Thermo Fisher Scientific, Karlsruhe, Germany). 1-(3-Acetyl-2,4,6-trihydroxyphenyl)ethanone (>98%) was purchased from Santa Cruz Biotechnology, Inc. (Heidelberg, Germany). Benzene-1,3,5-triol (≥99%), indole (≥99%), 4-hydroxy-6-methylpyran-2-one (≥99%) and n-hexane (GC-grade) were bought from Merck (Darmstadt, Germany). Nonanoic acid (≥97%) was obtained from Sigma (Vienna, Austria). Heptan-2-one (99%), pentan-2-one (≥99%), benzaldehyde (≥99%), 2-phenylacetic acid (99%), heptadec-1-ene (98%), heptadec-8-ene (≥96%), (*Z*)-octadec-9-enoic acid (≥99%) and (±)-3,7-Dimethyl-6-octenal (≥95%) were obtained from Sigma-Aldrich (Vienna, Austria). 3,7-Dimethylocta-2,6-dienoic acid (85%) was purchased from Aldrich (Vienna, Austria). 5,7-Dihydroxy-2-methylchromen-4-one (noreugenin, ≥99%) was obtained from MolPort (Riga, Latvia). Methyl-2-hydroxybenzoate (≥99.5%) and *n*-alkane standard solutions (C_8_–C_20_: 40 mg L^−1^ each in hexane and C_21_–C_40_: 40 mg L^−1^ each in toluene) were bought from Fluka (Vienna, Austria). Methanol (MeOH, LC-MS Chromasolv, HPLC grade) was obtained from Honeywell Riedel de Haën, (Seelze, Germany).

#### 4.2.2. GC-MS

For HS-SPME-GC-MS analysis, 10 mL HS-vials were used. For qualitative analysis of the MGR products by HS-SPME-GC-MS, the MGRCs (for detailed sample description see Table S1) were isolated by dissection and weighed directly into cooled HS vials before vials were sealed with 1.3 mm silicone/PTFE septa containing screw caps (La-Pha-Pack, Langerwehe, Germany; distributed by Markus Bruckner Analysentechnik, Linz, Austria). For HS-SPME-GC-MS measurements, the method described in Weingart et al. [64] was used with the following adaptions: For SPME, the samples were equilibrated for 40 min and extracted for 60 min, both at 50 °C. The gas chromatograph was fitted with a 30 m × 0.25 mm × 0.25 µm cross-linked 5%-phenyl 95%-methyl polysiloxane capillary column (HP-5MS UI, Agilent Technologies, Santa Clara, CA, USA). The oven program started with 35 °C (hold 2 min), followed by an increase of 20 °C min^–1^ to 205 °C, followed by a ramp of 10 °C min^−1^ to 250 °C, and then 25 °C min^−1^ to 300 °C (hold for 5 min). MS scan range was *m/z* 45–500 at a scan rate of approximately 3 scans sec^−1^.

Sample preparation for the qualitative analysis of the MGRC compounds via liquid-injection GC-MS was carried out similar to Hoenigsberger et al. [37]. Briefly summarized, the analytical samples, each consisting of MGRCs of 10 ants (Table S1), were extracted with ice-cold (to prevent chemical modifications) EtOAc in a ratio of 1 + 15 (*w*/*v*) by vortexing for 5 min under cooled conditions (laboratory thermoregulated to 7 °C), followed by centrifugation of the extraction mixture (10 min, 4400 rpm, 7 °C). For GC-MS measurement, 1 µL-aliquots of the obtained supernatants were injected into the split/splitless injector of an Agilent 7890 A gas chromatograph, fitted with an HP-5MS UI column coupled to an Agilent 5975 C mass selective detector. The same GC-MS parameters as for HS-SPME-GC-MS were used, but a solvent delay of 4.5 min was chosen. Qualitative analyses were carried out in splitless mode, as well as with split ratios of 10:1 and 60:1.

#### 4.2.3. Preparation of Standards, Retention Index (RI)-Calibrants, and Blanks

Individual standard stock solutions with a concentration of 1000 mg L^−1^ in ACN or EtOAc were prepared from the pure standard compounds and stored at −20 °C until further use. The standard stock solutions were diluted with ACN/H_2_O (1 + 1, *v*/*v*) in case of HS-SPME-GC-MS or with EtOAc in case of liquid-injection GC-MS to concentrations that resulted in narrow peak shapes during GC-MS analysis (1–100 mg L^−1^, depending on the components). For HS-SPME, 20 µL of the respective standard solutions were transferred into 10 mL HS-vials and analyzed as described for the MGRCs above. The RI values were established on the basis of a series of *n*-alkanes. Different volumes of the RI calibrant solutions and parameter settings for the SPME step were necessary to obtain narrow peak shapes for the RI calibrants: C_8_–C_20_: 8 μL in 10 mL HS-vial, 20 min incubation, and 10 min extraction both at 90 °C; C_21_–C_40_: 13 μL in 10 mL HS vial, 30 min incubation, and 60 min extraction both at 120 °C. For the C_8_–C_20_ mix, a solvent delay of 4.2 min and for the C_21_–C_40_ mix, a solvent delay of 6.5 min was chosen. For liquid-injection, the RI calibrant solution was prepared as follows: *n*-alkane standard solution C_8_–C_20_ + *n*-alkane standard solution C_21_–C_40_ + hexane (1 + 1 + 6 *v*/*v*/*v*). Empty vials, as well as vials containing 20 µL ACN/H_2_O (HS-SPME) or EtOAc (liquid-injection-GC-MS), were used to record background (blank) chromatograms with the aim to recognize substances originating from the ambient air, vials, septa, SPME fiber, solvents, or the GC-MS system.

### 4.3. Annotation and Identification of MGRC Compounds

Peak detection, spectrum deconvolution, RI calibration, and comparison of RIs and mass spectra against either an in-house or the Wiley Registry 10th Edition/NIST 2014 Mass Spectral Library [39] were carried out with the open source software MetaboliteDetector [36] version 3.1.Lisa20170127Ra-Linux current version available at [65]. The parameter settings as described in Hoenigsberger et al. [37] were used with the following modifications for spectrum deconvolution: a) Peak Settings: peak threshold 5 and b) minimum peak height 5. Based on literature search for volatile metabolites already described in defensive secretions of ants, e.g., The Pherobase [66] and initial GC-MS measurements, an in-house library consisting of RIs and mass spectra of 132 authentic reference standards was created by the use of MetaboliteDetector. Compounds detected in the MGRC extracts of *C. explodens* for which the overall similarity score (OSS, [36]), considering a combination of spectrum similarity and RI similarity score, was equal or greater than 0.9 compared to the in-house library entries, were designated as identified. Since OSS calculation of unknown compounds not included in the in-house library was not supported by MetaboliteDetector, annotation for compounds not matching to any of the in-house library entries was based on a separate comparison of a) spectrum similarity with the implemented Wiley Registry 10th Edition/NIST 2014 Mass Spectral Library [39] and b) RI values of the NIST Chemistry Webbook [38]. If the spectrum similarity score was equal or greater than 0.9 and the reference RI for the compound listed in the NIST Chemistry Webbook (for similar stationary phase, film thickness, and column diameter) matched to the experimentally derived RI (deviation maximum ± 1%), the compound was designated as annotated. In cases of multiple literature RI values, the median RI of that respective substance was used for comparison. Only compounds for which the criteria for identification or annotation were fulfilled in ≥50% of the tested analytical samples are mentioned in this manuscript. For peak area determination, one quantification ion (QI) per compound was chosen for processing with the batch quantification tool of MetaboliteDetector (parameter settings: Compound matching: ΔRI 5; pure/impure 0.5. Identification: ΔRI 10; pure/impure 0.5. Quantification: Redetect all quant. ions and extended SIC scan activated).

### 4.4. Confirmatory Analysis of MAPG and Noreugenin by LC-HRMS/MS.

MGRC extracts obtained after first extraction with EtOAc (see 4.2.2) were further diluted 1 + 999 (*v*/*v*) with methanol/water/formic acid (1 + 1 + 0.1%, *v*/*v*/*v*) and analyzed with a high resolution mass spectrometer Orbitrap Q Exactive HF (Thermo Fisher Scientific, Boston, MA, USA) equipped with a heated electrospray ionisation (HESI) source coupled to a Liquid Chromatography (LC) system (Vanquish, Thermo Fisher Scientific, Boston, MA, USA). Chromatographic separation was carried out using a reversed-phase XBridge C18 column (2.1 mm × 150 mm, 3.5 µm particle size, Waters, Milford, MA, USA), which was preceded by a C18 4 × 3 mm security cartridge (Phenomenex, Torrance, CA, USA). The column temperature was kept constant at 25 °C. The mobile phase consisted of water + 0.1% formic acid (eluent A) and MeOH + 0.1% formic acid (eluent B), and the flow rate was set to 0.25 mL min^−1^. After 2 min at 10% B, a linear gradient up to 100% B was reached within 30 min and 100% B were kept constant for 5 min. Then, the column was re-equilibrated at 10% B for 8 min. Samples were kept at 10 °C in the autosampler unit. Then, 2 µL-aliquots of the diluted samples were injected into the LC-HRMS system. The HESI-source was operated in fast polarity switching mode (positive/negative ionization). Mass spectra were recorded in full MS mode as well as the data dependent MS/MS mode using an inclusion list where MS/MS spectra were only recorded if predefined ions of the inclusion list ([M + H]^+^ (*m/z* 169.0495) and [M − H]^−^ (*m/z* 167.0350) for MAPG, and [M + H]^+^ (*m/z* 193.0495) and [M − H]^−^ (*m/z* 191.0350) for noreugenin) were detected in the preceding full scan. Settings: a) full scan: scan range, *m/z* 100–1000; R = 120,000 at *m/z* 200; profile mode; b) MS/MS: fragmentation in HCD cell with stepped collision energy (CE 25, 35, 45); R = 15,000 at *m/z* 200; profile mode. A standard solution containing the two compounds MAPG and noreugenin (2 mg L^−1^ in methanol/water/formic acid (1 + 1 + 0.1%)) was injected within the same sequence for confirmation. Data evaluation was performed using the vendor Thermo XCalibur software (Thermo Fisher Scientific, Boston, MA, USA). To test the similarity of the MS/MS spectra overlap between samples and standard solutions the classic score, that displays a ratio of the unknown spectrum (*i.e.,* sample spectrum) to the library spectrum (*i.e.,* standard spectrum), was calculated with the help of the software mzVault (Version 2.1; Thermo Fisher Scientific, Boston, MA, USA).

### 4.5. Statistical Analysis of Compounds Identified after Liquid-Injection GC-MS

For statistical analysis of the constituents identified and annotated after liquid-injection GC-MS, their GC-MS abundance values (*i.e.,* EIC peak areas) determined by the batch quantification tool of MetaboliteDetector (see also 4.3.), were used. The abundance values of the compounds identified after operation of the gas chromatograph in split mode were multiplied with the respective split ratio, and missing values in the data matrix (n = 1) were replaced with zero. The resulting matrix was autoscaled and mean-centered [67]. Then, a principal component analysis (PCA) and a hierarchical cluster analysis (HCA) (squared Euclidean distance, Ward linkage) were constructed. To generate the log10-scaled heatmap, all abundance values of the imputed data matrix were increased by one and transformed with the common logarithm (log10). All calculations were performed in the R environment (v. 3.1.0) for statistical calculations [68].

## Figures and Tables

**Table 1:** Volatile organic compounds (VOCs) identified or annotated in the mandibular gland reservoir content (MGRC) of *C. explodens*.

				HS-SPME-GC-MS				Liquid-Extraction-GC-MS			
ID	VOC	Trivial Name	PubChem CID	RI_Ref_	RI_Exp_	SSS	OSS	RI_Ref_	RI_Exp_	SSS	OSS
1	Pentan-2-one ^1^	Ethyl acetone	7895	660	660	0.96	0.97				
2	Pentane-2,4-dione ^1^	Acetylacetone	31261	766	767	1.00	1.00				
3	Hept-5-en-2-one or isomer *^,3^		5363108	866	874		0.93	866	872	0.91	
4	Heptan-2-one ^1^		8051	891	898	0.98	0.90	891	897	1.00	0.93
5	Benzaldehyde ^1^		57418027	967	969	0.94	0.96				
6	Trimethylbenzene isomer 1 *^,3^ possibly 1,3,5-trimethylbenzene		7947					979	986	0.96	
7	Trimethylbenzene isomer 2 *^,3^ possibly 1,2,4-trimethylbenzene		7247					990	991	0.98	
8	*n*-Decane ^1^		15600	1000	1001	0.99	0.99	1000	1002	0.94	0.95
9	Hexanoic acid ^1^	Caproic acid	8892	1013	1019	0.96	0.91	985	985	0.88	0.91
10	Undec-1-ene ^1^		13190	1092	1094	0.96	0.96				
11	2-Methoxyphenol ^1^	Guaiacol	460	1095	1097	1.00	0.99	1095	1097	0.96	0.97
12	*n*-Undecane ^1^		14257	1100	1101	0.99	0.99	1100	1102	1.00	0.99
13	(±)-3,7-Dimethyloct-6-enal ^1^	(±)-Citronellal	7794	1157	1158	0.97	0.98				
14	Benzoic acid ^1^		243	1173	1174	0.93	0.95				
15	Benzene-1,2-diol ^1^	Catechol	289					1197	1197	0.96	0.97
16	*n*-Dodecane ^1^		8182	1200	1201	0.94	0.95				
17	Methyl 2-hydroxybenzoate ^1^	Methyl salicylate	4133	1206	1207	1.00	1.00	1206	1208	0.98	0.98
18	2-Phenylacetic acid ^1^	Benzeneacetic acid	999	1255	1257	0.96	0.97	1252	1253	0.88	0.92
19	Nonanoic acid ^1^	Pelargonic acid	8158	1271	1270	0.90	0.93	1270	1268	0.95	0.96
20	Tridec-1-ene ^1^		17095	1294	1296	0.98	0.98				
21	*n*-Tridecane ^1^		12388	1300	1302	0.99	0.98	1300	1301	0.99	0.99
22	1H-indole ^1^	Indole	798					1308	1309	1.00	1.00
23	3,7-Dimethylocta-2,6-dienoic acid ^1^	Geranic acid	5275520					1360	1358	0.91	0.94
24	5-Methylbenzene-1,3-diol ^1^	Orcinol	10436					1377	1373	0.99	0.96
25	3-Acetyl-6-methylpyran-2,4-dione ^1^	Dehydroacetic acid	122903					1382	1382	0.98	0.98
26	1-(2-hydroxy-4,5-dimethylphenyl)ethanone ^1^		118976	1440	1444	0.96	0.95				
27	4-Hydroxy-6-methylpyran-2-one ^1^	Triacetic acid lactone	54675757					1442	1436	1.00	0.94
28	Pentadec-1-ene ^1^		25913	1493	1492	0.96	0.97				
29	*n*-Pentadecane ^1^		12391	1500	1502	0.99	0.99	1500	1501	0.99	0.99
30	Benzene-1,3,5-triol ^1^	Phloroglucinol	359					1593	1593	0.99	0.99
31	*n*-Hexadecane ^1^	Cetane	11006	1600	1601	0.99	0.99	1600	1601	0.97	0.98
32	Heptadec-8-ene ^1^		5364555	1681	1680	0.96	0.97	1681	1682	0.97	0.98
33	Heptadec-1-ene ^1^		23217	1694	1693	0.99	0.99				
34	*n*-Heptadecane ^1^		12398	1700	1704	0.98	0.97	1700	1702	1.00	0.99
35	*n*-Octadecane ^1^		11635	1800	1800	0.95	0.97				
36	Hexadecan-1-al ^1^	Palmitaldehyde	984					1821	1822	0.85	0.90
37	Hexadecan-1-ol ^1^	Cetyl alcohol	2682	1882	1882	0.88	0.92				
38	1-(2,4,6-Trihydroxyphenyl)ethanone ^1,^^∆^	Monoacetylphloroglucinol	68073					1885	1883	0.92	0.94
39	*n*-Nonadecane ^1^		12401	1900	1900	0.98	0.99				
40	Heptadecan-2-one or isomer *^,3^		18027	1899	1905	0.90					
41	5,7-Dihydroxy-2-methylchromen-4-one ^1,^^∆^	Noreugenin	5375252					2003	2000	1.00	0.98
42	1-(3-Acetyl-2,4,6-trihydroxyphenyl)ethanone ^1^	2,4-Diacetylphloroglucinol	16547					2020	2016	0.96	0.95
43	Octadecan-1-ol ^1^	Stearyl alcohol	8221	2090	2089	0.91	0.91				
44	(Z)-Octadec-9-enoic acid ^1^	Oleic acid	445639					2140	2146	0.92	0.90
45	Octadecanoic acid *^,2^	Stearic acid	5281	2178	2167	0.90		2178	2164	0.93	

ID: Numbering of compounds corresponds to the elution order of VOCs in the respective chromatograms as shown in Figure 2; RI_Ref_: RI of reference (standard) compound; RI_Exp_…Experimentally derived RI of compound; SSS: Spectrum similarity score depicting the spectra similarity between experimental and reference compound, as given by MetaboliteDetector software; OSS: Overall similarity score, which combines RI and mass spectrum similarity between experimental and reference compound measured under identical conditions in parallel. This OSS is a measure for the agreement between experimental and literature RI and mass spectrum ([for OSS calculation, see [36,37]). For OSS values for each compound in each sample file refer to Table S2; ^∆^ Identity additionally confirmed with LC-HRMS/MS, see 2.2; ^1^ Identified compounds (confidence level 1 identification, [35]); ^2^ Annotated compounds (confidence level 2, [35]); ^3^ Annotated compounds (confidence level 3, [35]); * Annotation is based on similarities of RIs and mass spectra provided by the National Institute of Standards and Technology (NIST) Chemistry WebBook and the Wiley Registry 10^th^ Edition/NIST 2014 Mass Spectral Library implemented in MetaboliteDetector [38,39]. For this table the RIs and similarity scores as obtained from the two samples of Cexpl_5/2014_1 were used. Exceptions are those for compounds with IDs 13, 19, and 37 (identified after HS-SPME-GC-MS) and with ID 44 (identified after liquid-extraction-GC-MS), for which identification was not possible in samples of Cexpl_5/2014_1. Instead, another sample was chosen to exemplify RIs and similarity scores.

## Supplementary Materials

The following are available online at https://www.mdpi.com/1420-3049/24/19/3468/s1. Table S1. *C. explodens* samples analyzed in this study; Table S2. Overall similarity scores for identified or annotated VOCs in single sample files as obtained by MetaboliteDetector data evaluation; Table S3. Identified or annotated VOCs in the MGRC of *C. explodens* reported from ants or other insects in the literature; Table S4. Data matrix used for statistical analysis of VOCs identified or annotated after liquid-injection GC-MS. Characteristics (structures, RIs, mass spectra, identifier, etc.) of compounds mentioned in Table 1 are compiled in the Supplementary File Compounds.pdf.
