# Supplementary material for: Volatiles from the Mandibular Gland Reservoir Content of Colobopsis explodens Laciny and Zettel, 2018, Worker Ants (Hymenoptera: Formicidae)
Source: Molecules. 2019 Sep 24;24(19):3468. doi: 10.3390/molecules24193468 (PMC6804081; doi:10.3390/molecules24193468)

Pentan−2−one

HS−SPME−GC−MS

splitless

ID: 1

RI: 660

PubChem CID: 7895

https://pubchem.ncbi.nlm.nih.gov/compound/7895

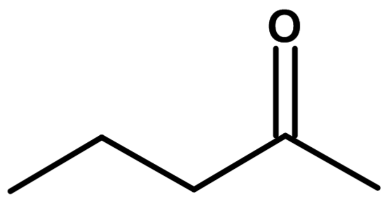

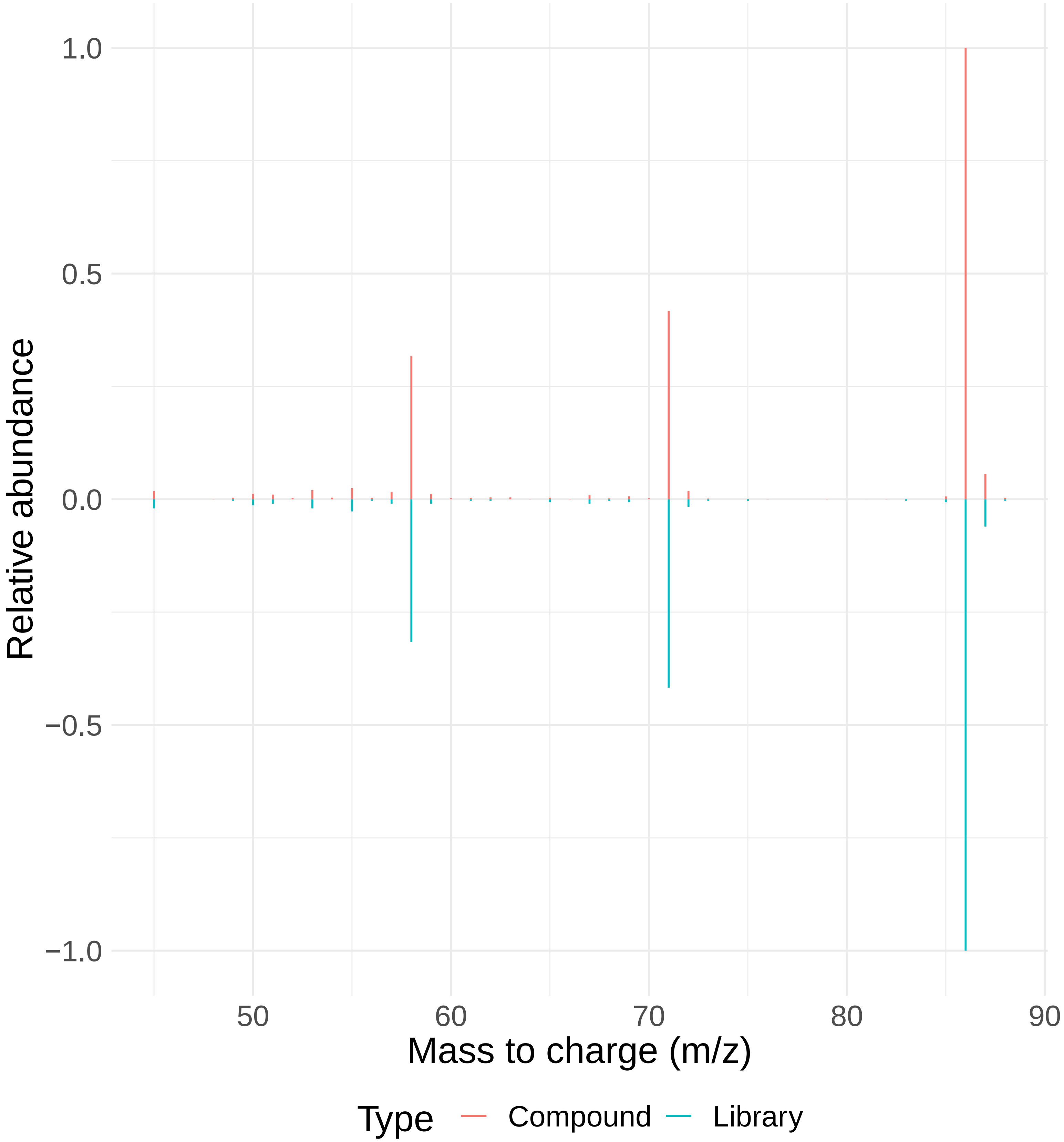

Pentane−2,4−dione

HS−SPME−GC−MS

splitless

ID: 2

RI: 767

PubChem CID: 31261

https://pubchem.ncbi.nlm.nih.gov/compound/31261

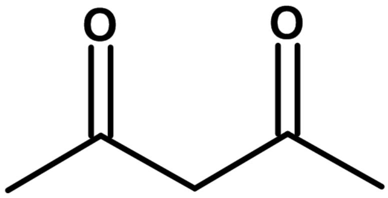

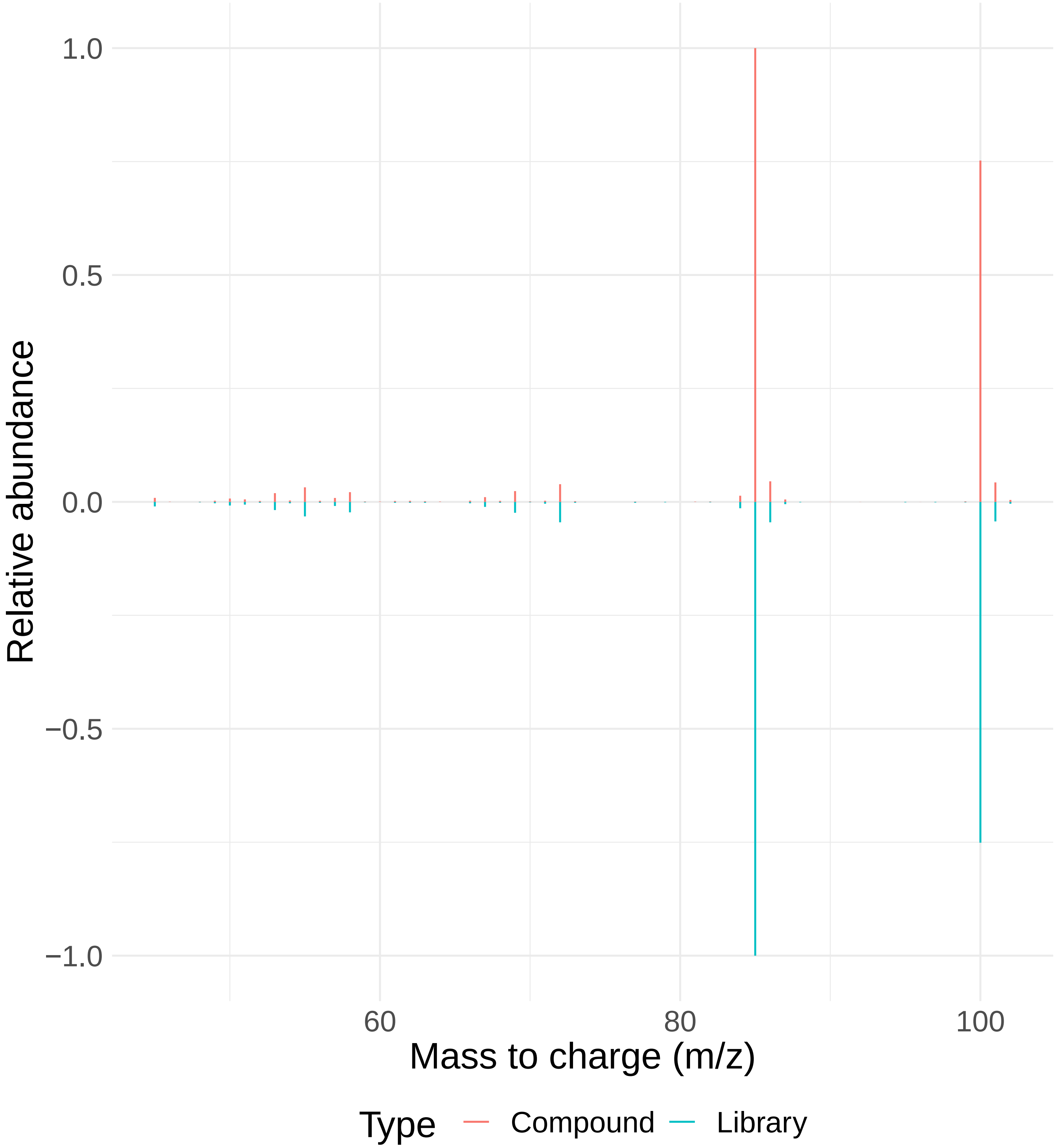

5−Hepten−2−one or isomer

HS−SPME−GC−MS

splitless

ID: 3

RI: 874

PubChem CID: 5363108

https://pubchem.ncbi.nlm.nih.gov/compound/5363108

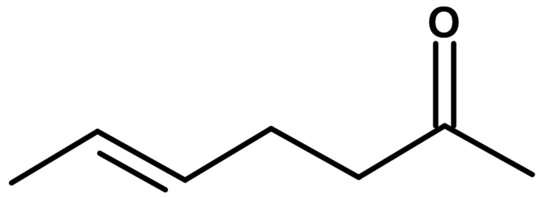

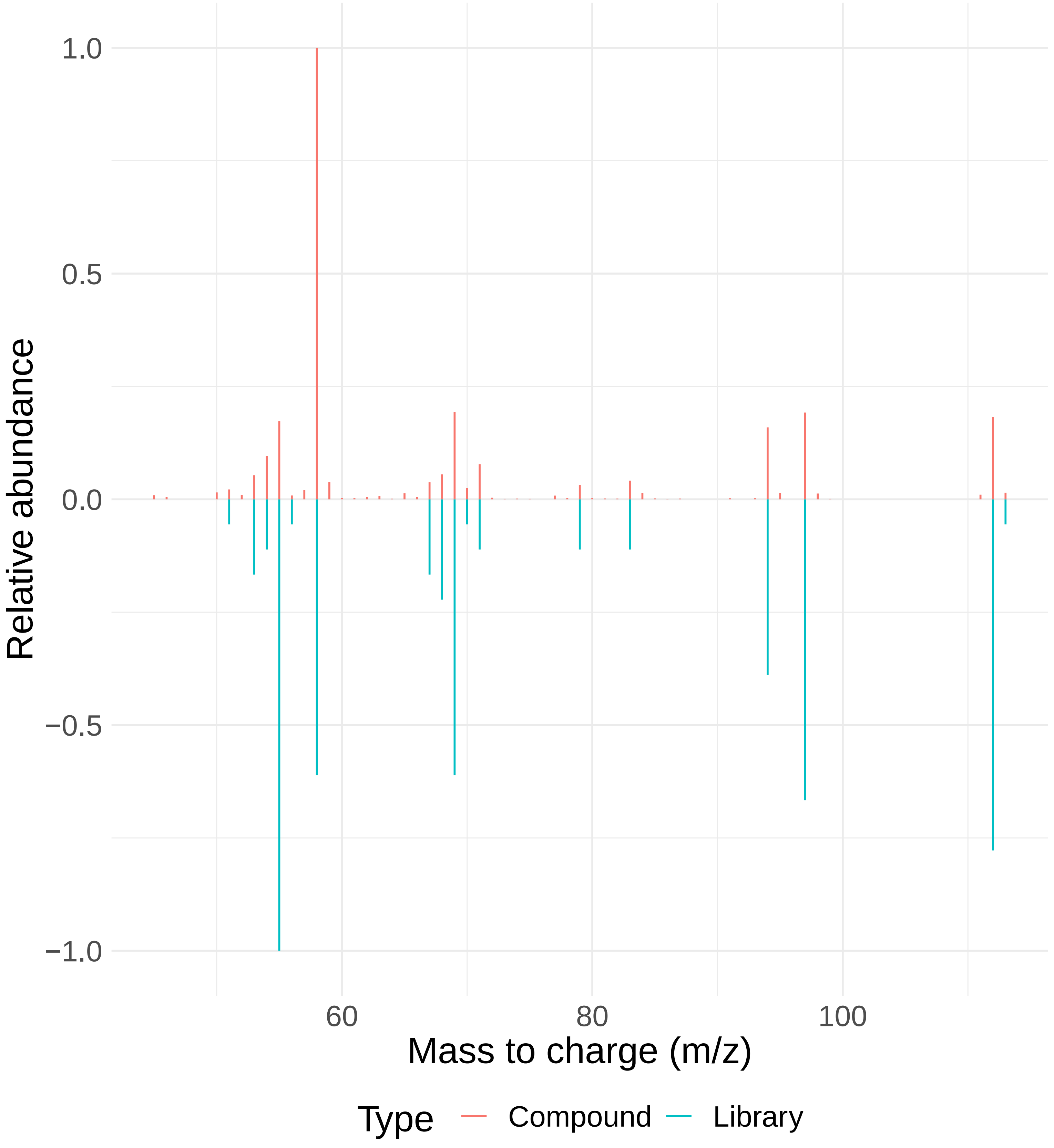

Heptan−2−one

HS−SPME−GC−MS

splitless

ID: 4

RI: 898

PubChem CID: 8051

https://pubchem.ncbi.nlm.nih.gov/compound/8051

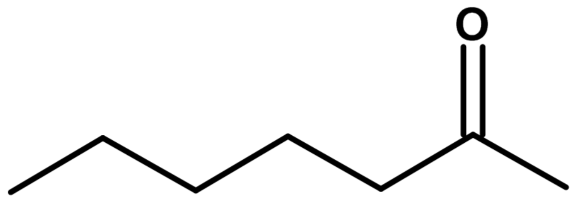

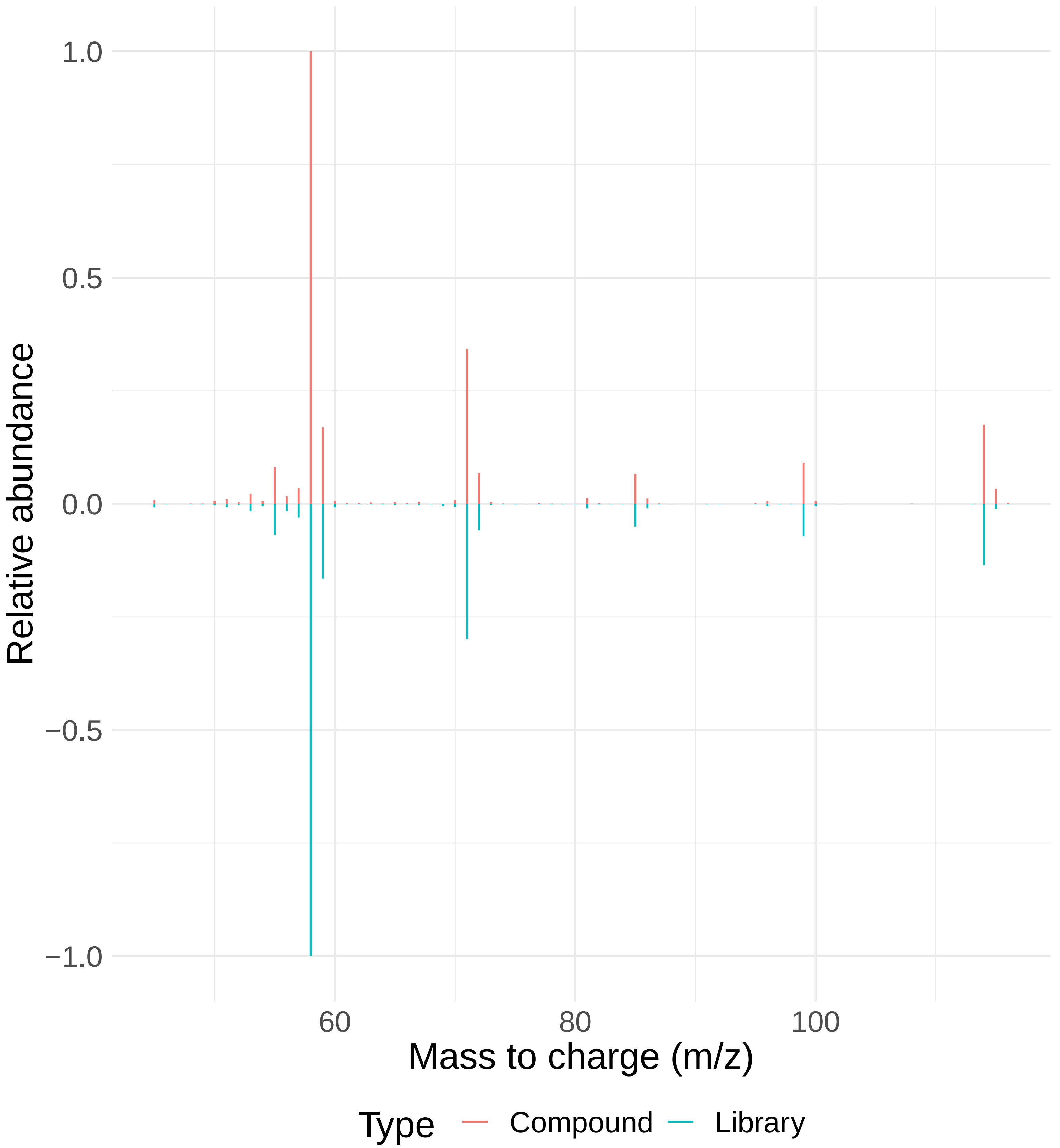

Benzaldehyde

HS−SPME−GC−MS

splitless

ID: 5

RI: 969

PubChem CID: 57418027

https://pubchem.ncbi.nlm.nih.gov/compound/57418027

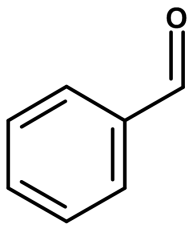

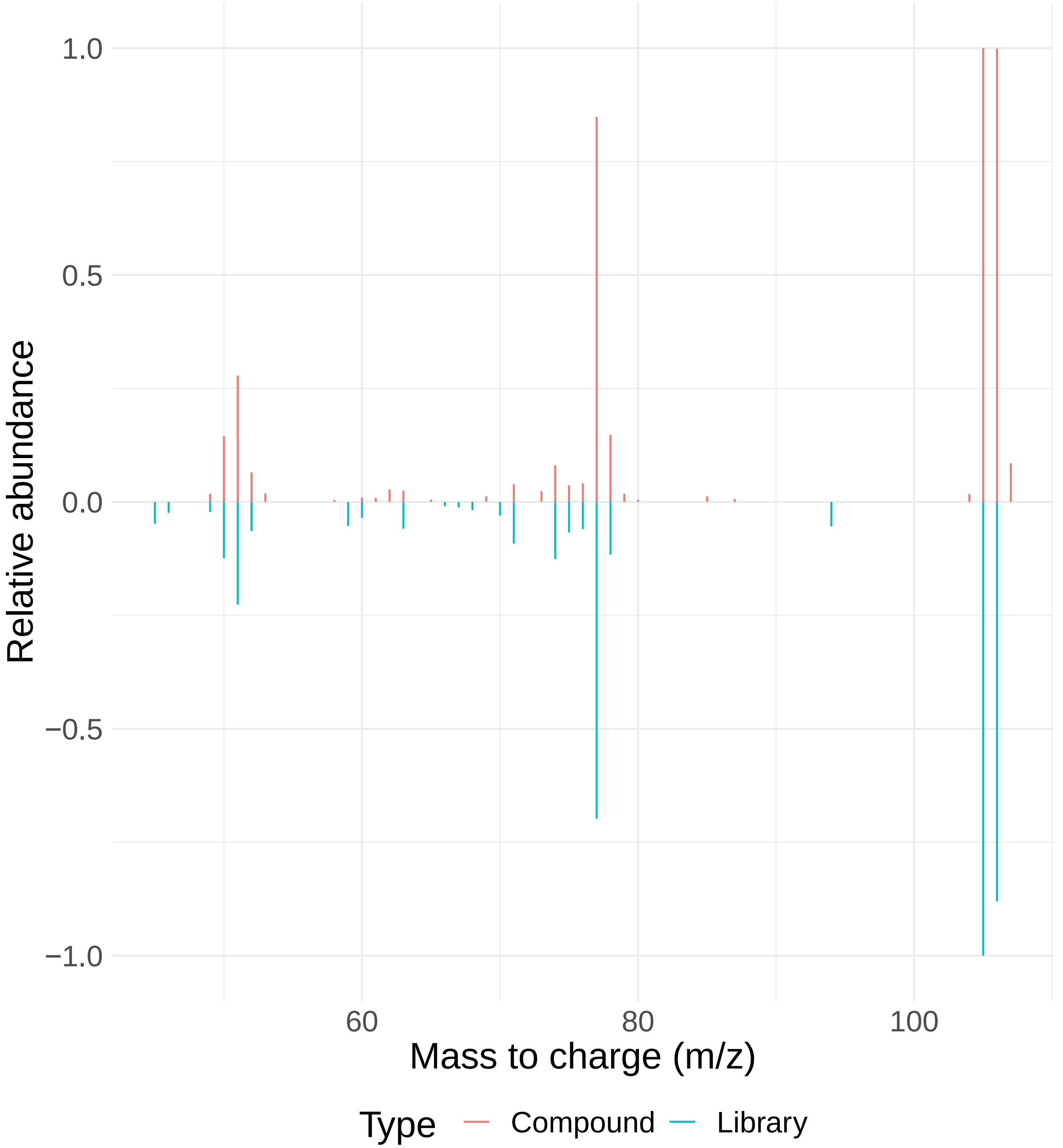

Trimethylbenzene isomer 1

Liquid−injection−GC−MS

splitless

ID: 6

RI: 986

PubChem CID: 7947

https://pubchem.ncbi.nlm.nih.gov/compound/7947

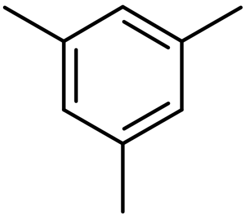

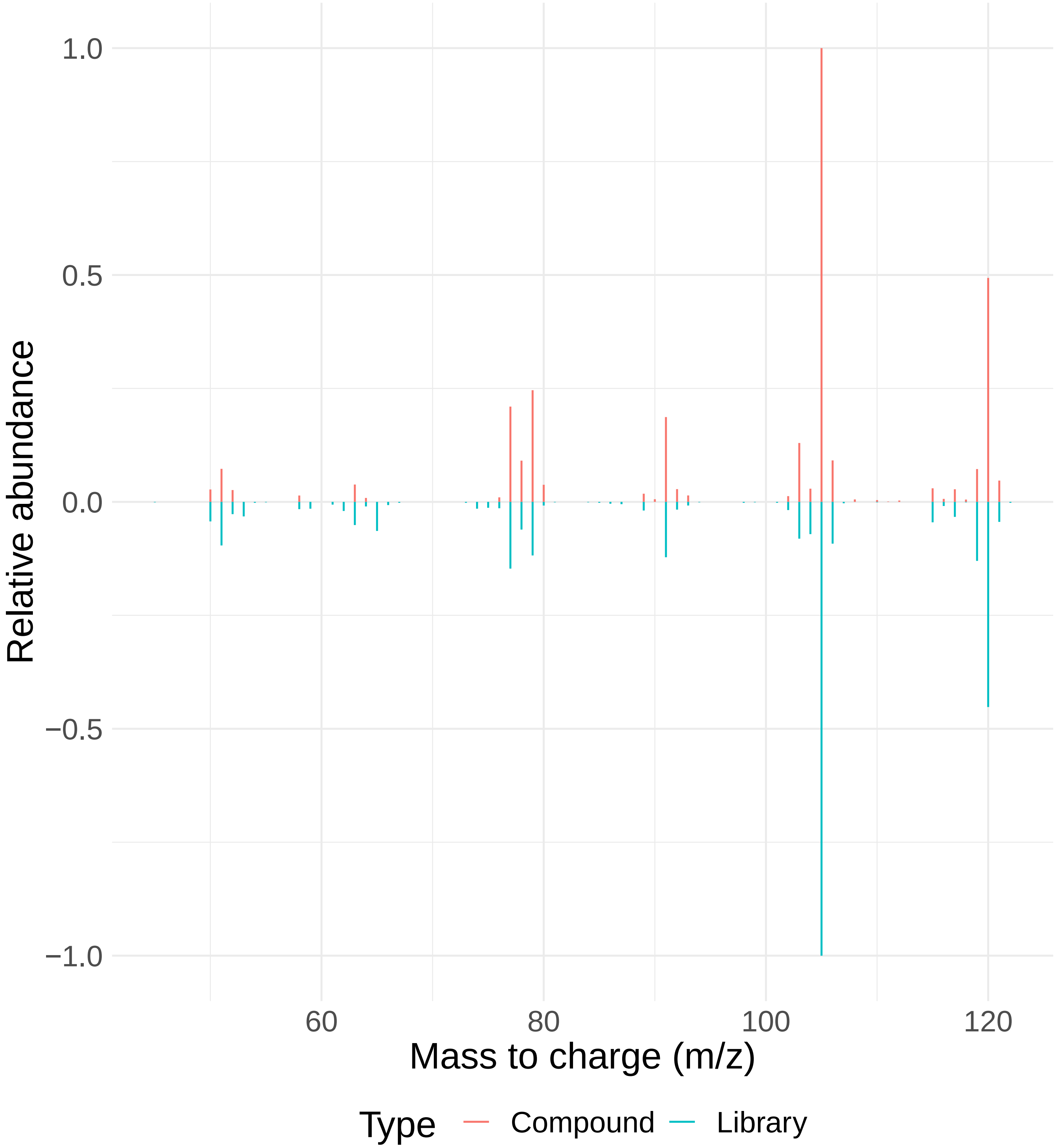

Trimethylbenzene isomer 2

Liquid−injection−GC−MS

splitless

ID: 7

RI: 991

PubChem CID: 7247

https://pubchem.ncbi.nlm.nih.gov/compound/7247

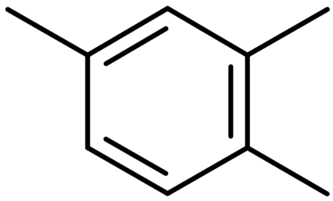

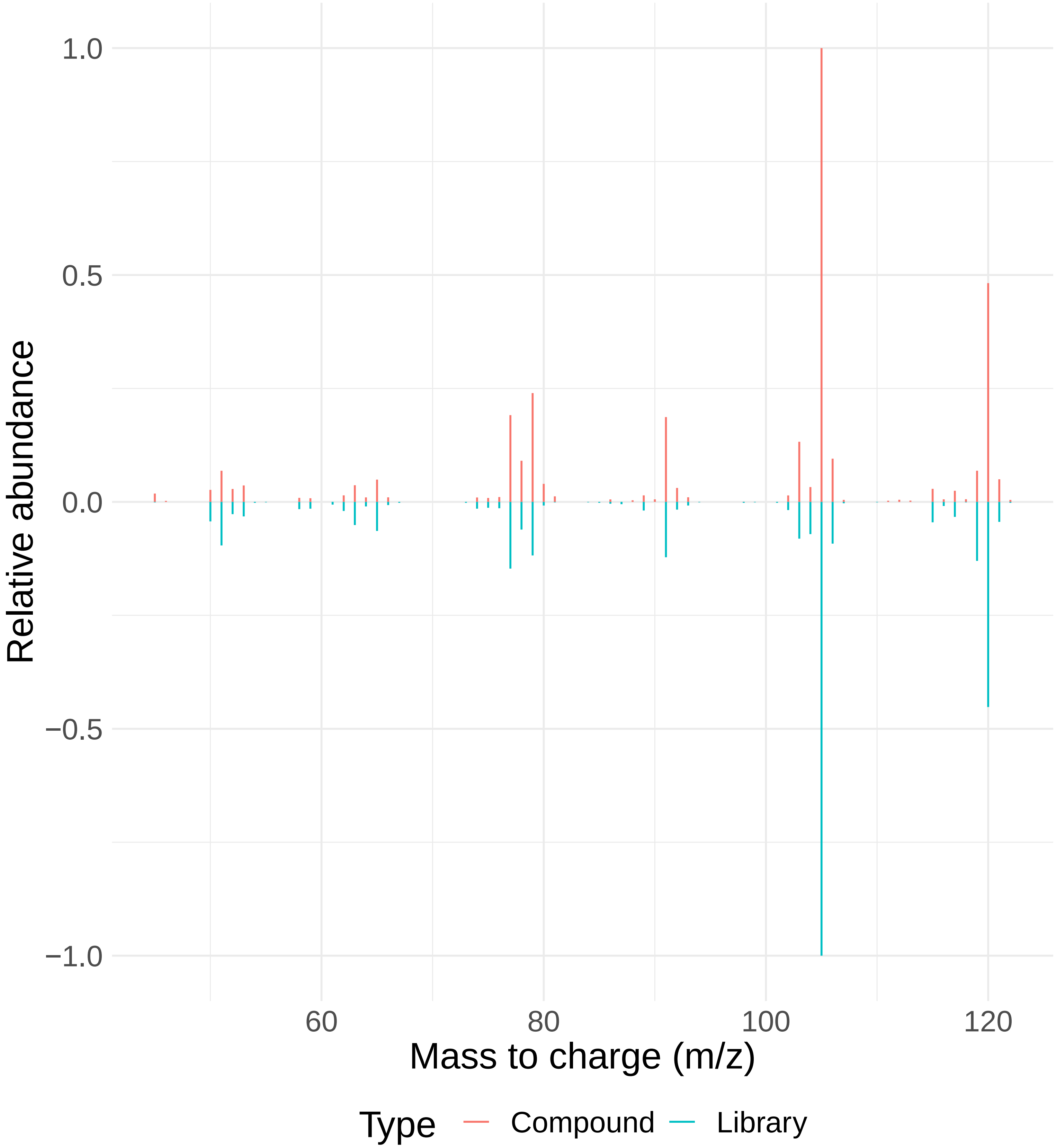

n−Decane

HS−SPME−GC−MS

splitless

ID: 8

RI: 1001

PubChem CID: 15600

https://pubchem.ncbi.nlm.nih.gov/compound/15600

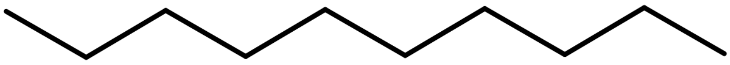

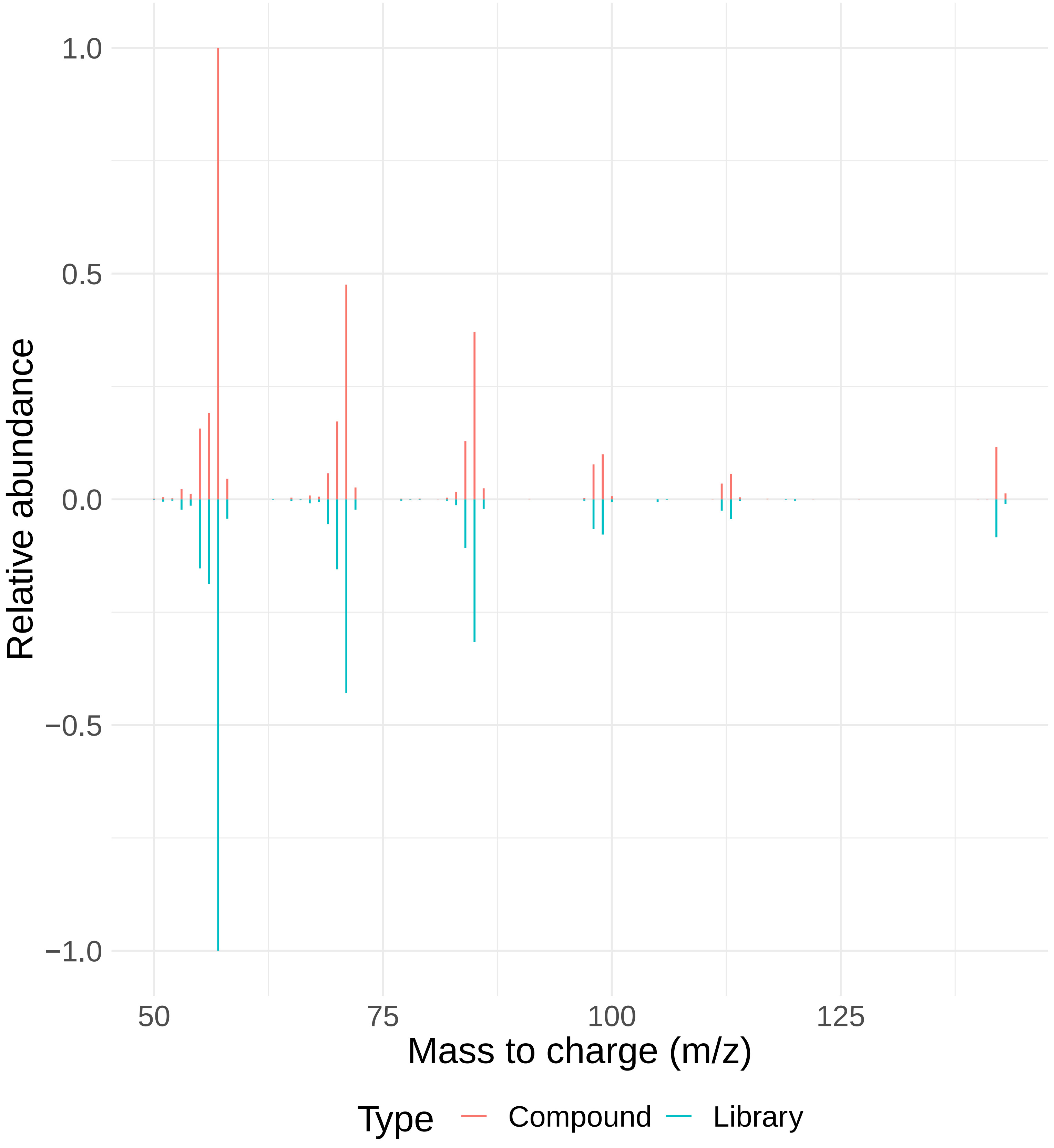

Hexanoic acid

HS−SPME−GC−MS

splitless

ID: 9

RI: 1019

PubChem CID: 8892

https://pubchem.ncbi.nlm.nih.gov/compound/8892

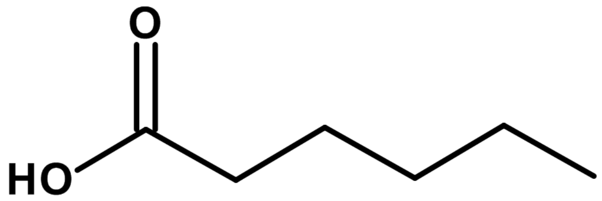

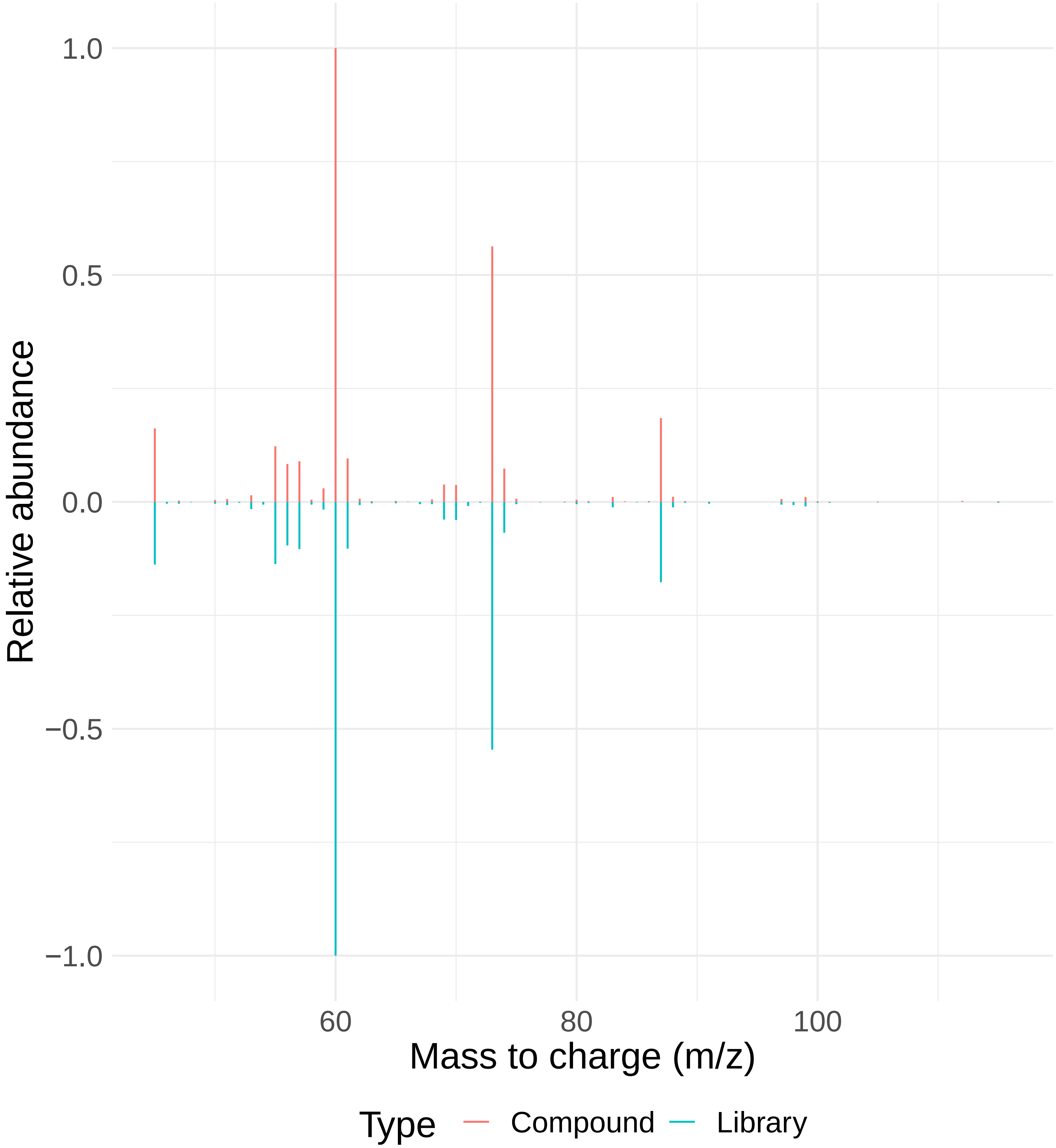

Undec−1−ene

HS−SPME−GC−MS

splitless

ID: 10

RI: 1094

PubChem CID: 13190

https://pubchem.ncbi.nlm.nih.gov/compound/13190

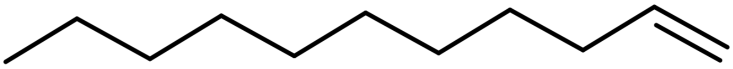

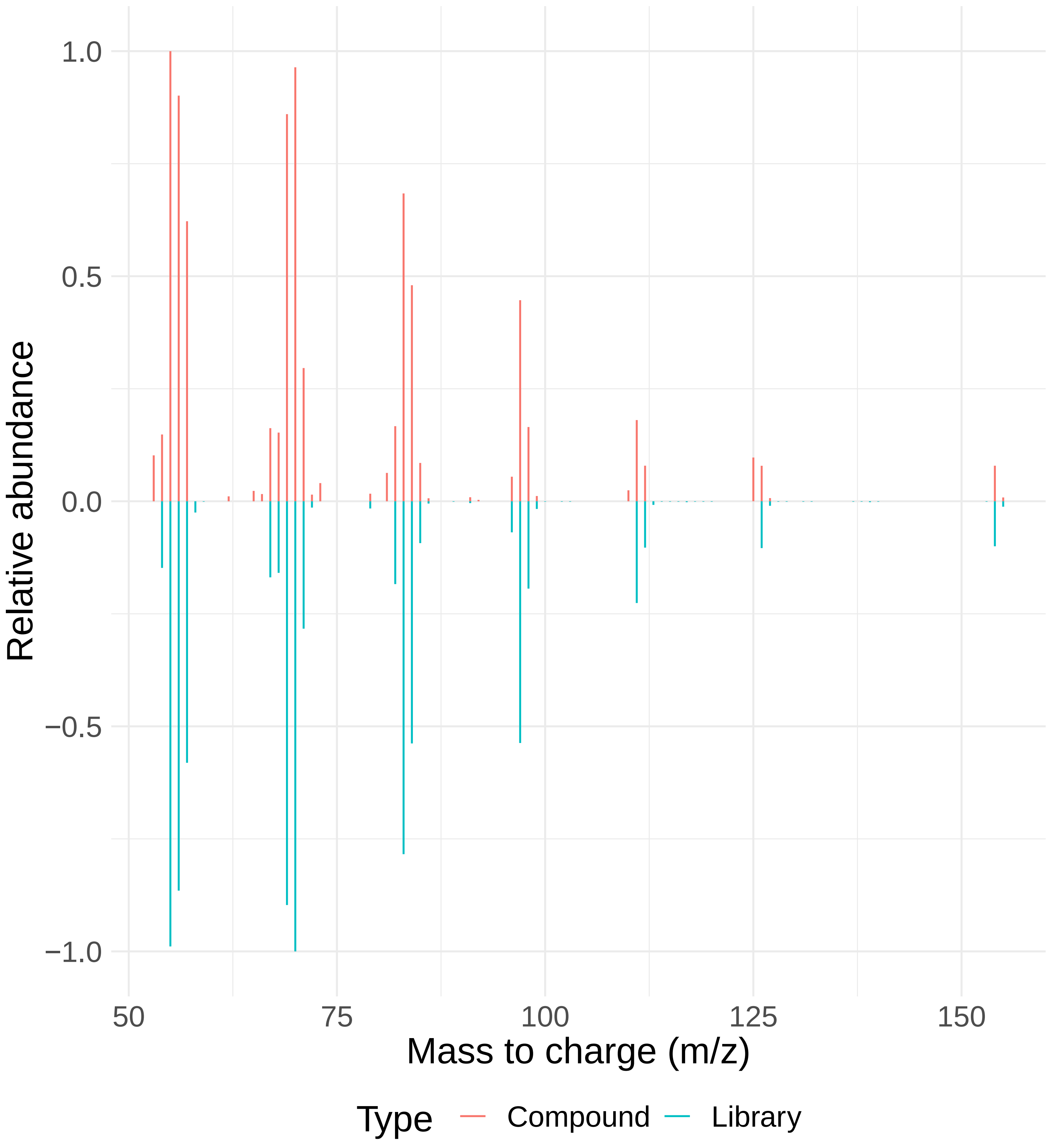

2−Methoxyphenol

HS−SPME−GC−MS

splitless

ID: 11

RI: 1097

PubChem CID: 460

https://pubchem.ncbi.nlm.nih.gov/compound/460

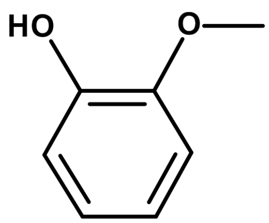

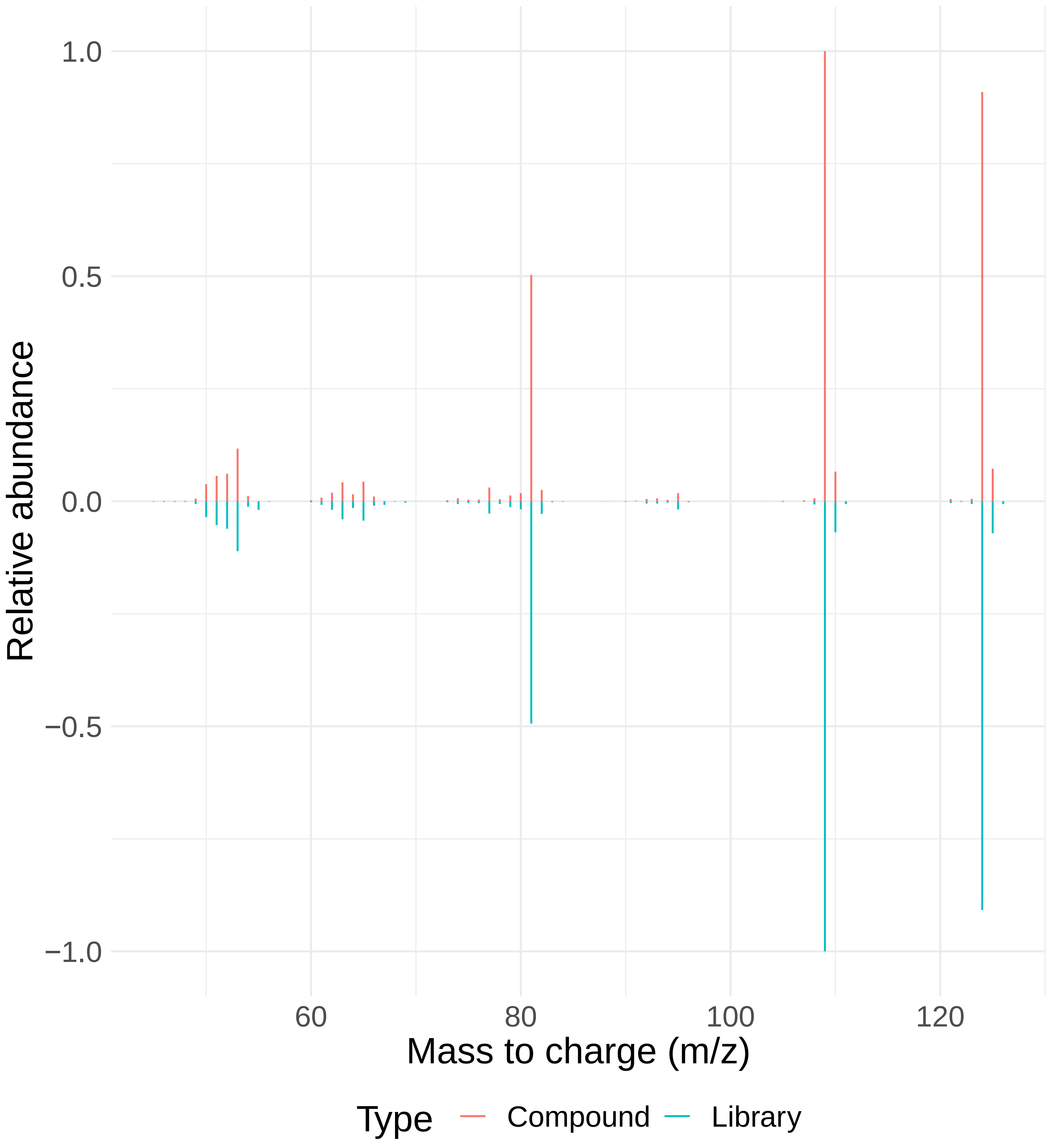

n−Undecane

HS−SPME−GC−MS

splitless

ID: 12

RI: 1101

PubChem CID: 14257

https://pubchem.ncbi.nlm.nih.gov/compound/14257

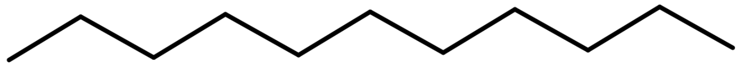

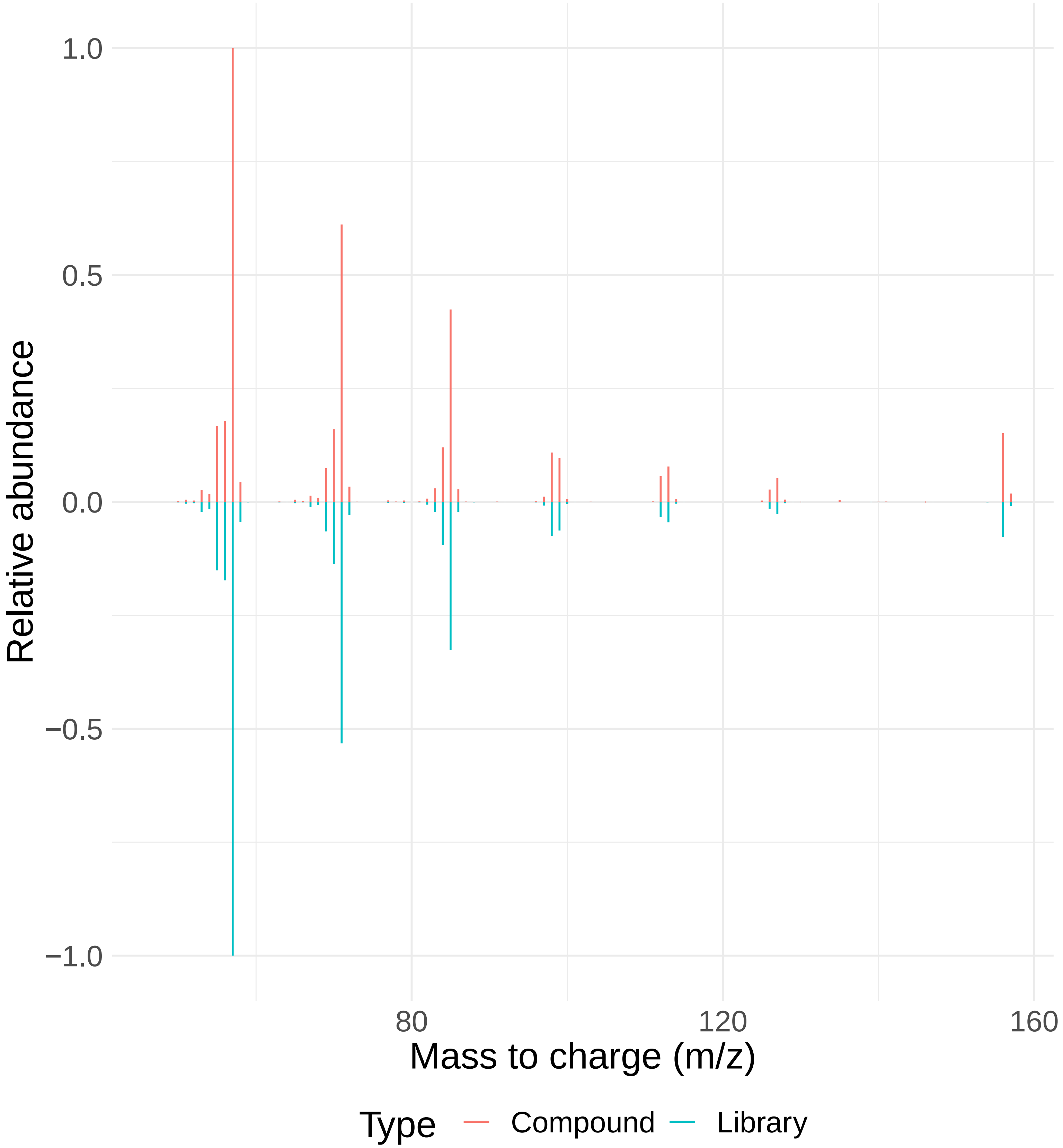

3,7−Dimethyloct−6−enal

HS−SPME−GC−MS

splitless

ID: 13

RI: 1158

PubChem CID: 7794

https://pubchem.ncbi.nlm.nih.gov/compound/7794

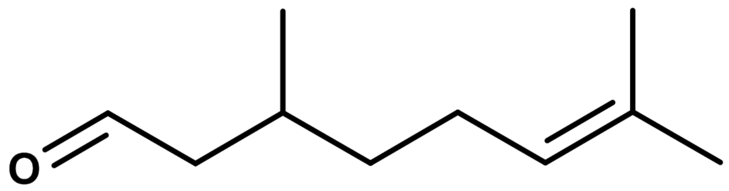

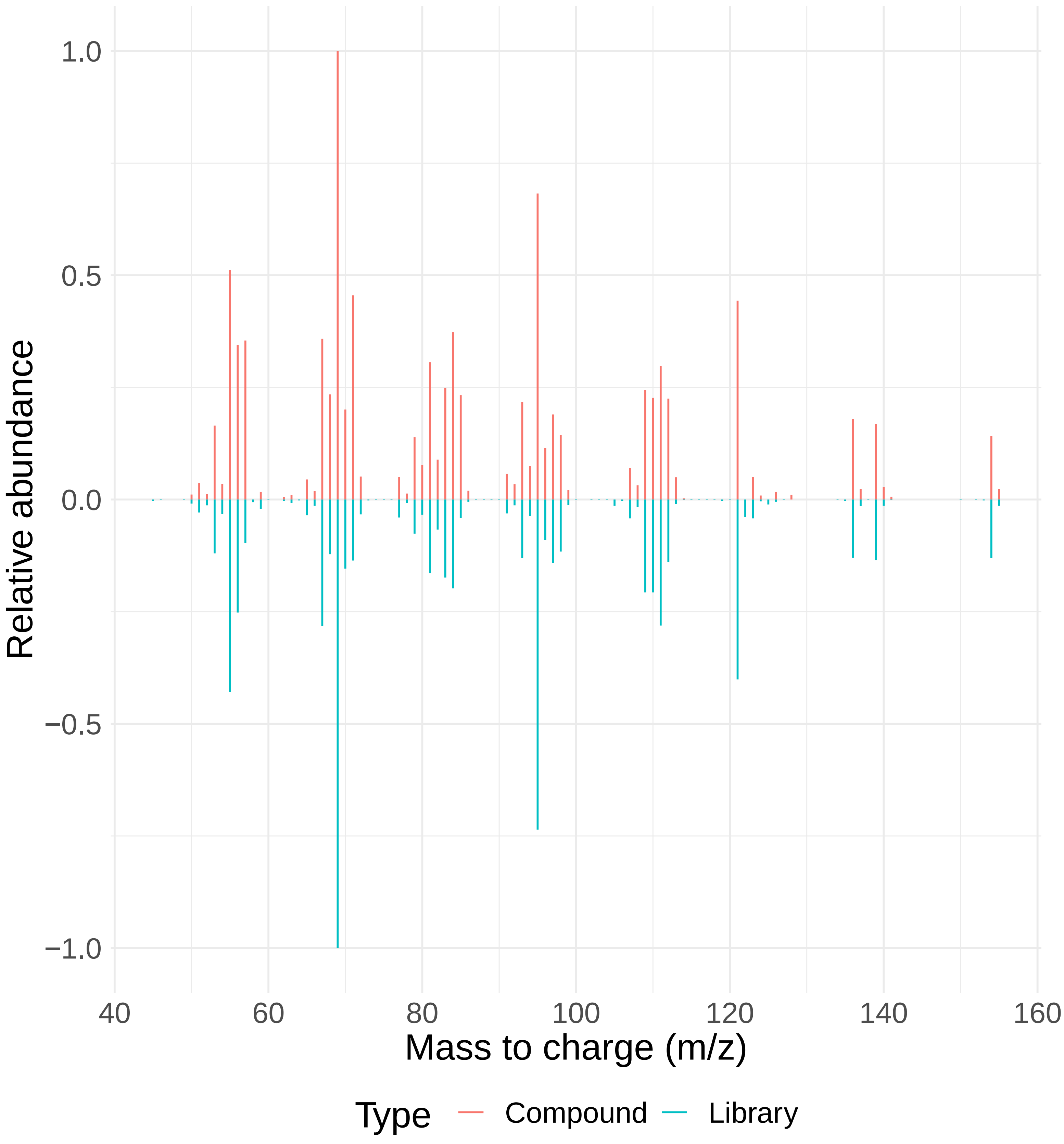

Benzoic acid

HS−SPME−GC−MS

splitless

ID: 14

RI: 1174

PubChem CID: 243

https://pubchem.ncbi.nlm.nih.gov/compound/243

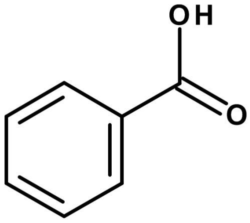

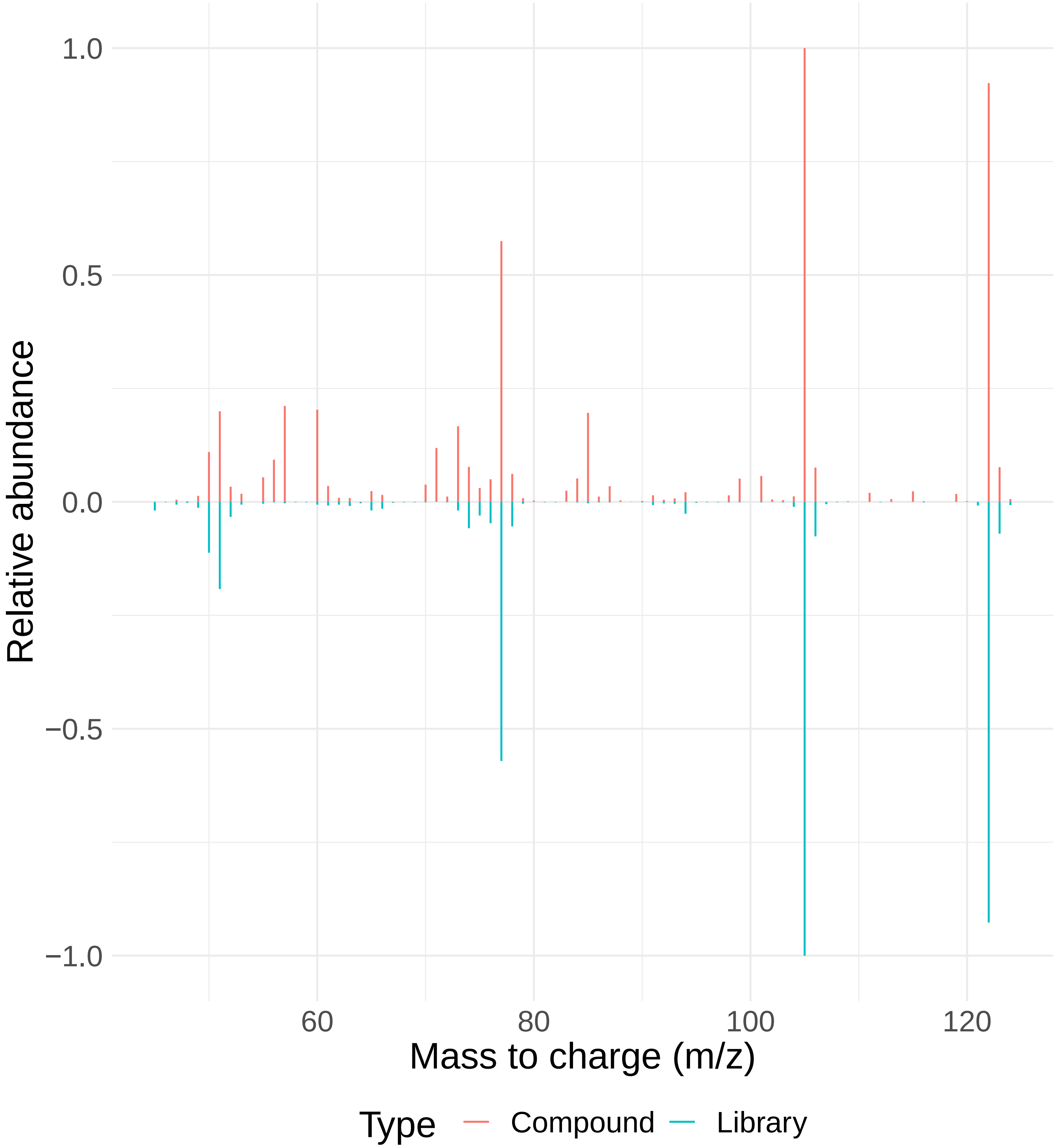

Benzene−1,2−diol

Liquid−injection−GC−MS

splitless

ID: 15

RI: 1197

PubChem CID: 289

https://pubchem.ncbi.nlm.nih.gov/compound/289

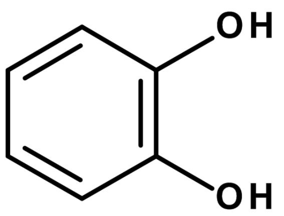

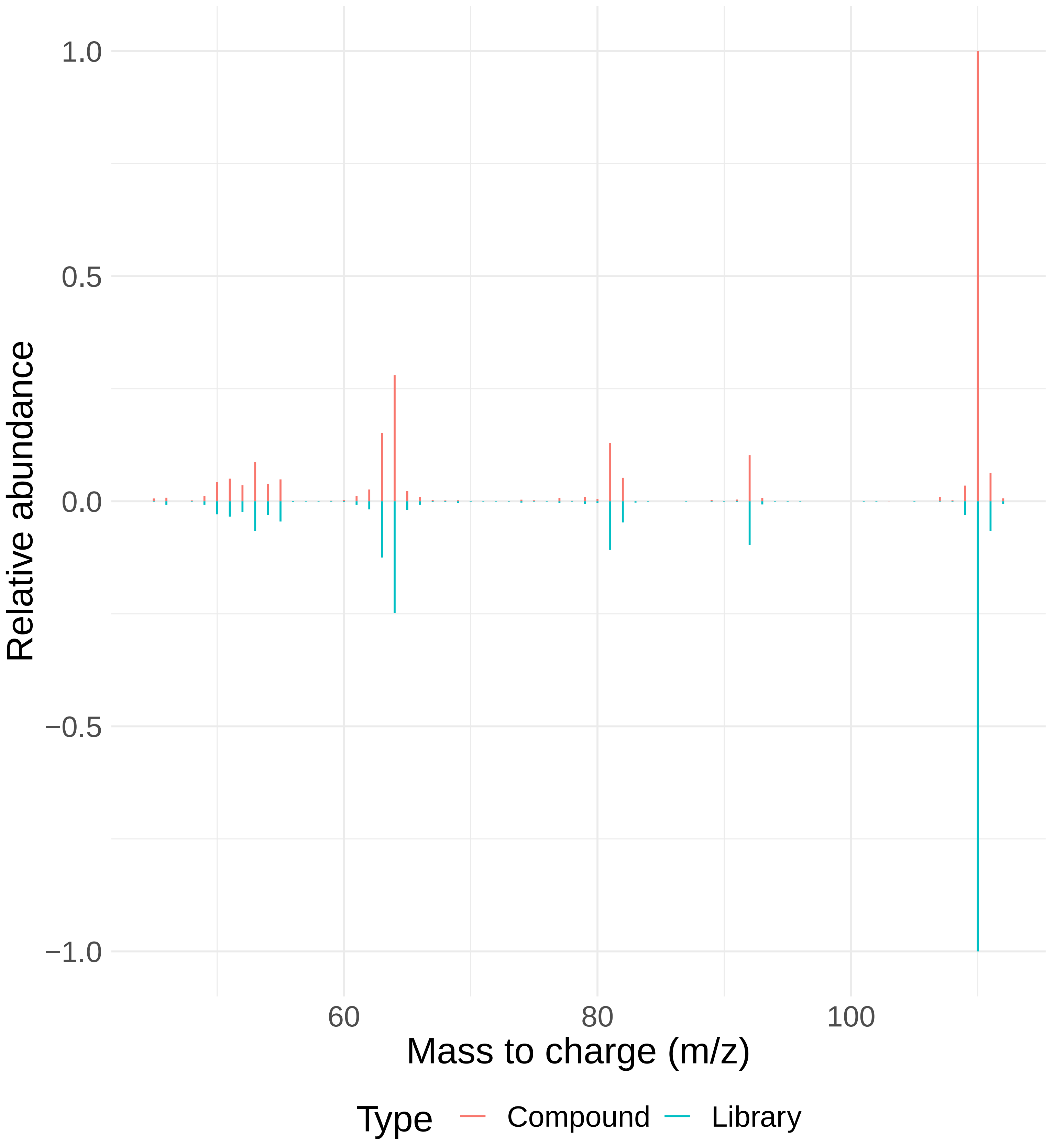

n−Dodecane

HS−SPME−GC−MS

splitless

ID: 16

RI: 1201

PubChem CID: 8182

https://pubchem.ncbi.nlm.nih.gov/compound/8182

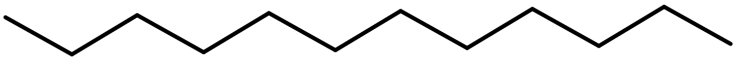

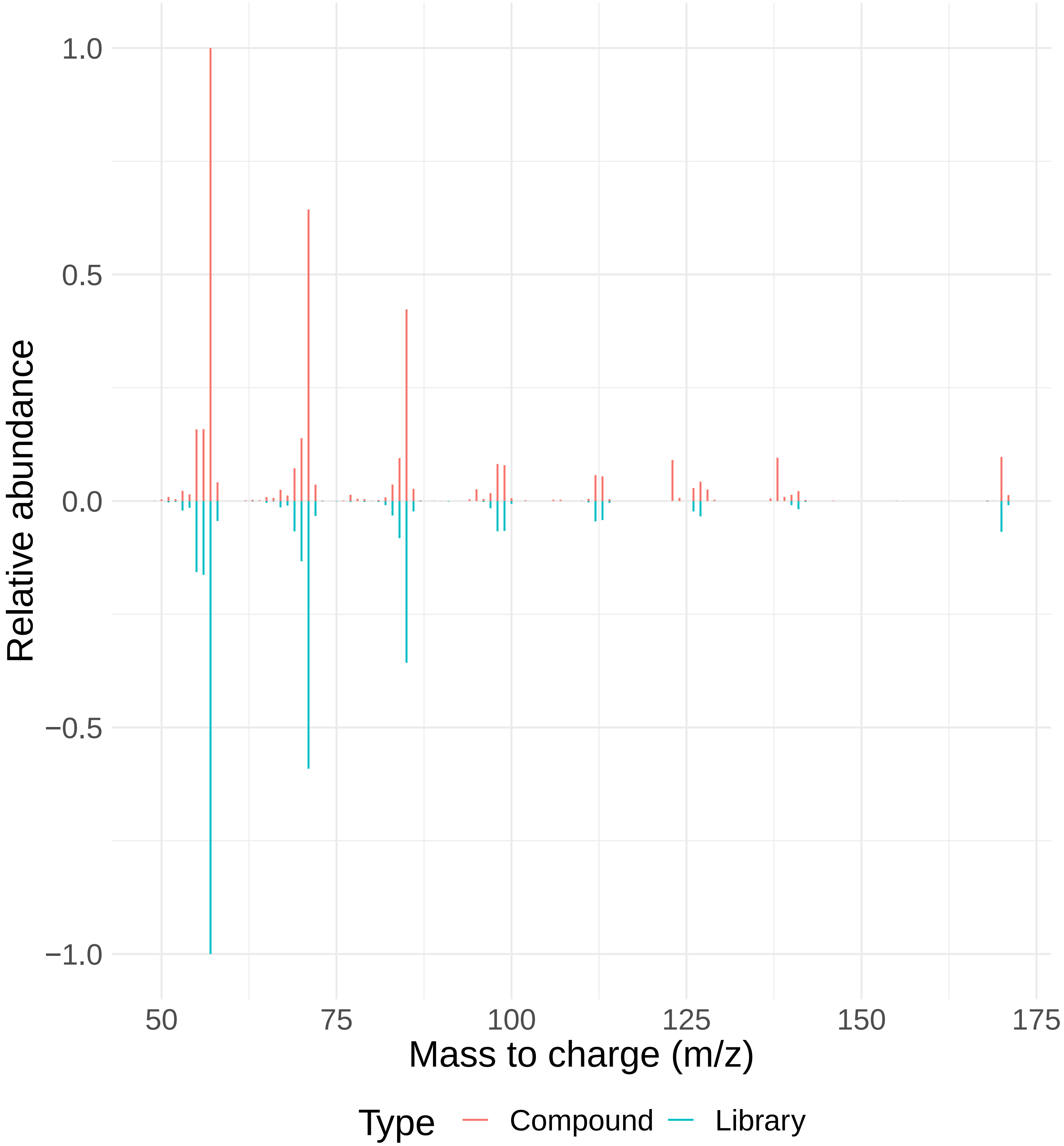

Methyl 2−hydroxybenzoate

HS−SPME−GC−MS

splitless

ID: 17

RI: 1207

PubChem CID: 4133

https://pubchem.ncbi.nlm.nih.gov/compound/4133

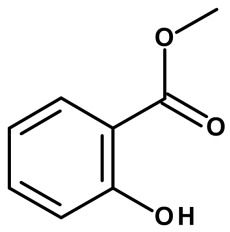

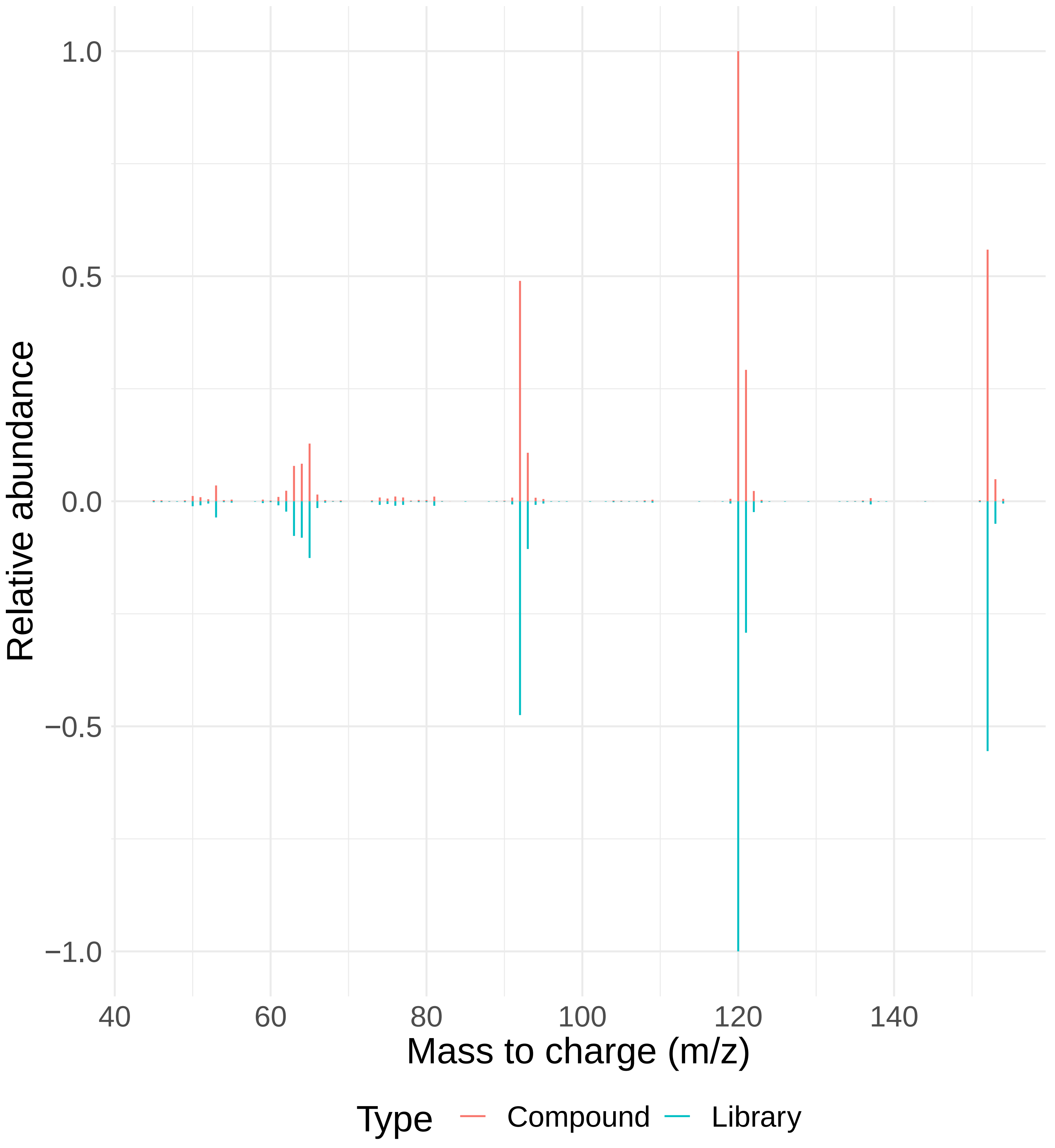

2−Phenylacetic acid

HS−SPME−GC−MS

splitless

ID: 18

RI: 1257

PubChem CID: 999

https://pubchem.ncbi.nlm.nih.gov/compound/999

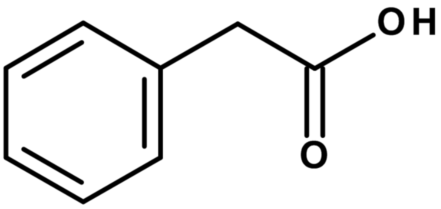

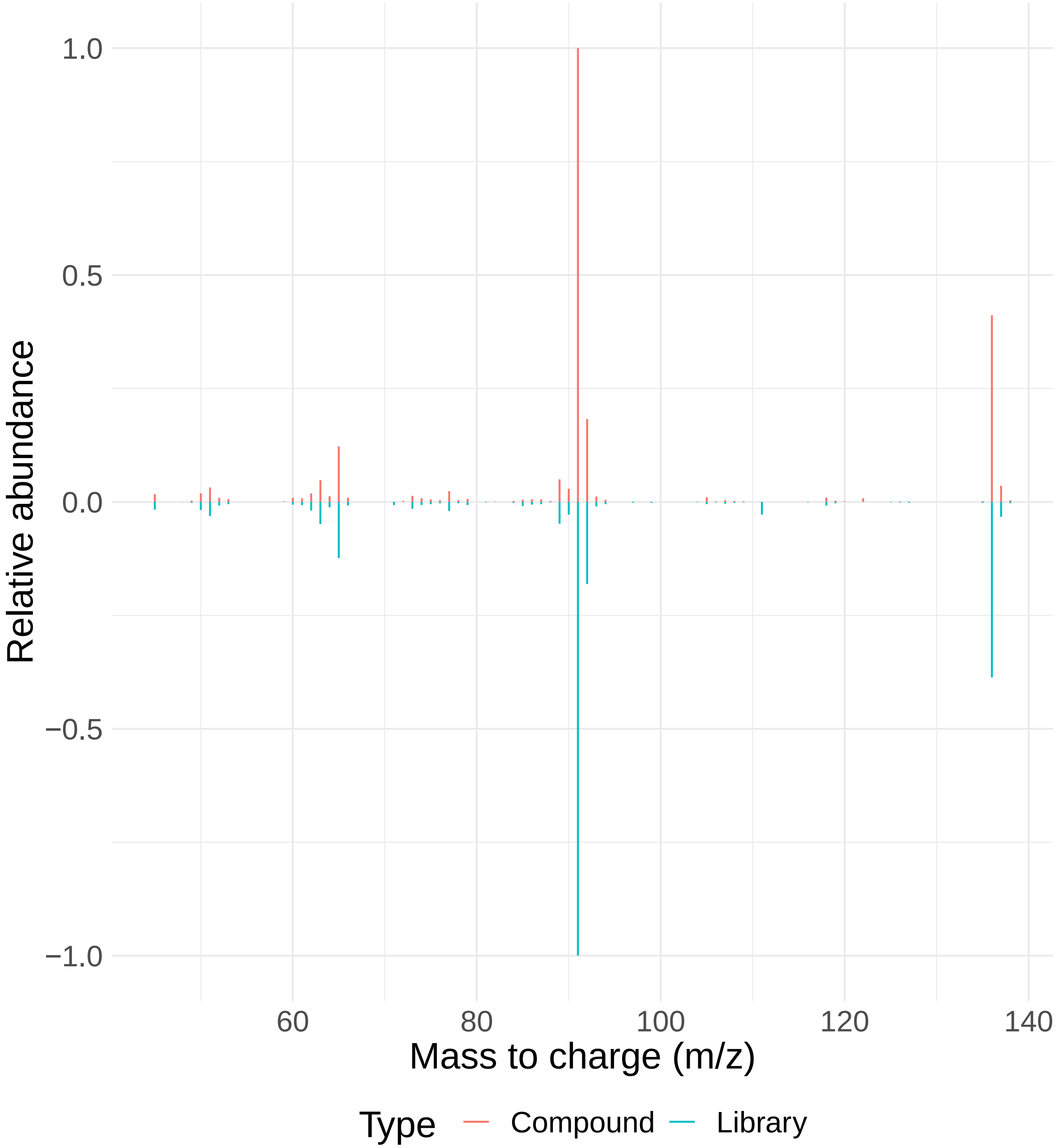

Nonanoic acid

HS−SPME−GC−MS

splitless

ID: 19

RI: 1270

PubChem CID: 8158

https://pubchem.ncbi.nlm.nih.gov/compound/8158

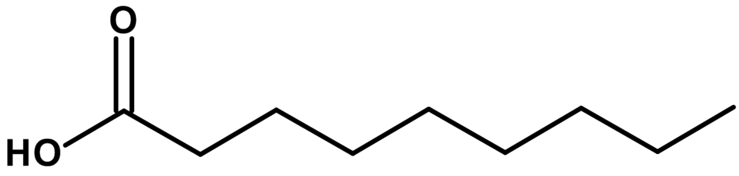

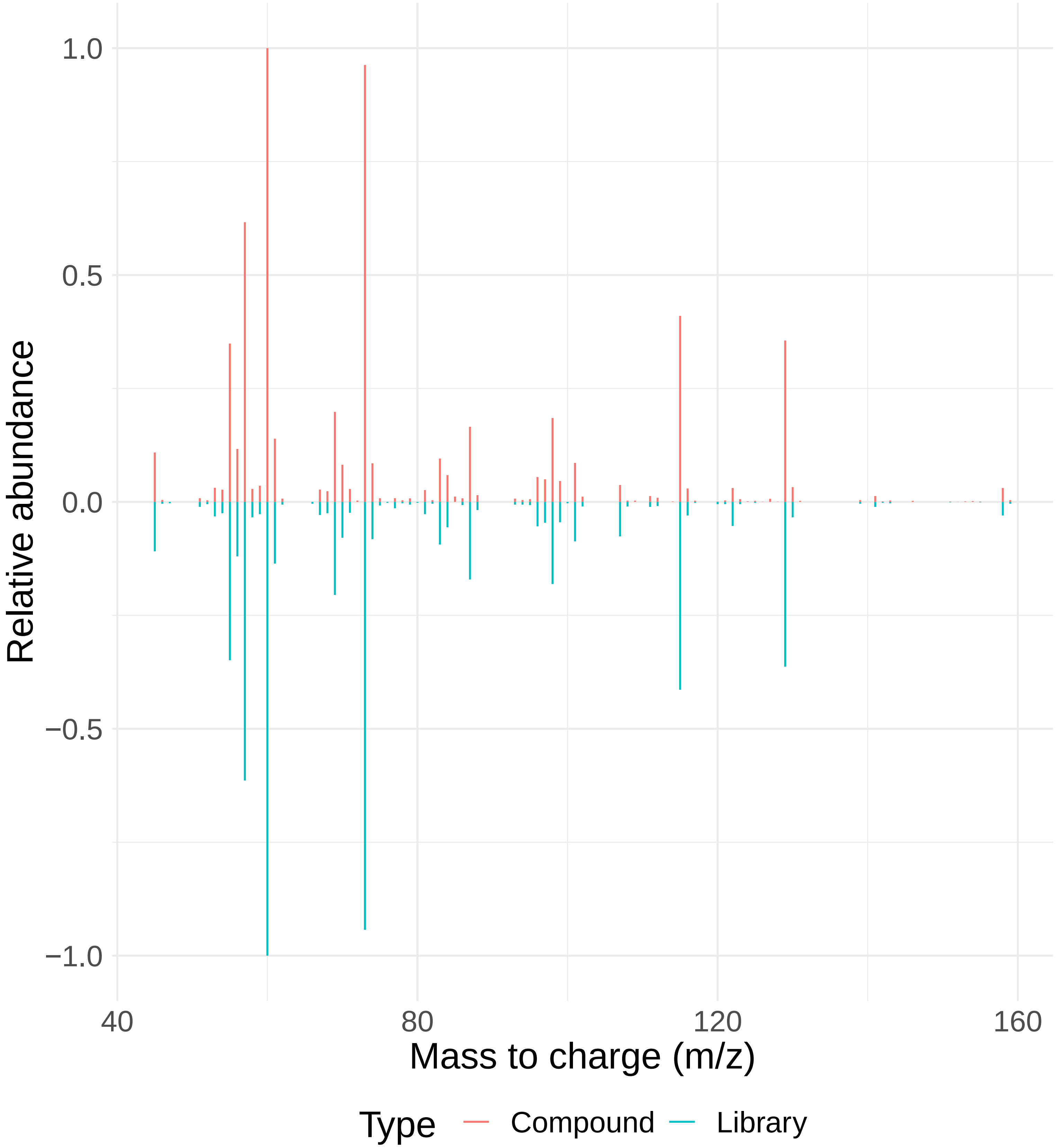

Tridec−1−ene

HS−SPME−GC−MS

splitless

ID: 20

RI: 1296

PubChem CID: 17095

https://pubchem.ncbi.nlm.nih.gov/compound/17095

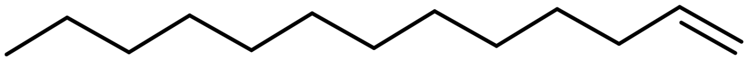

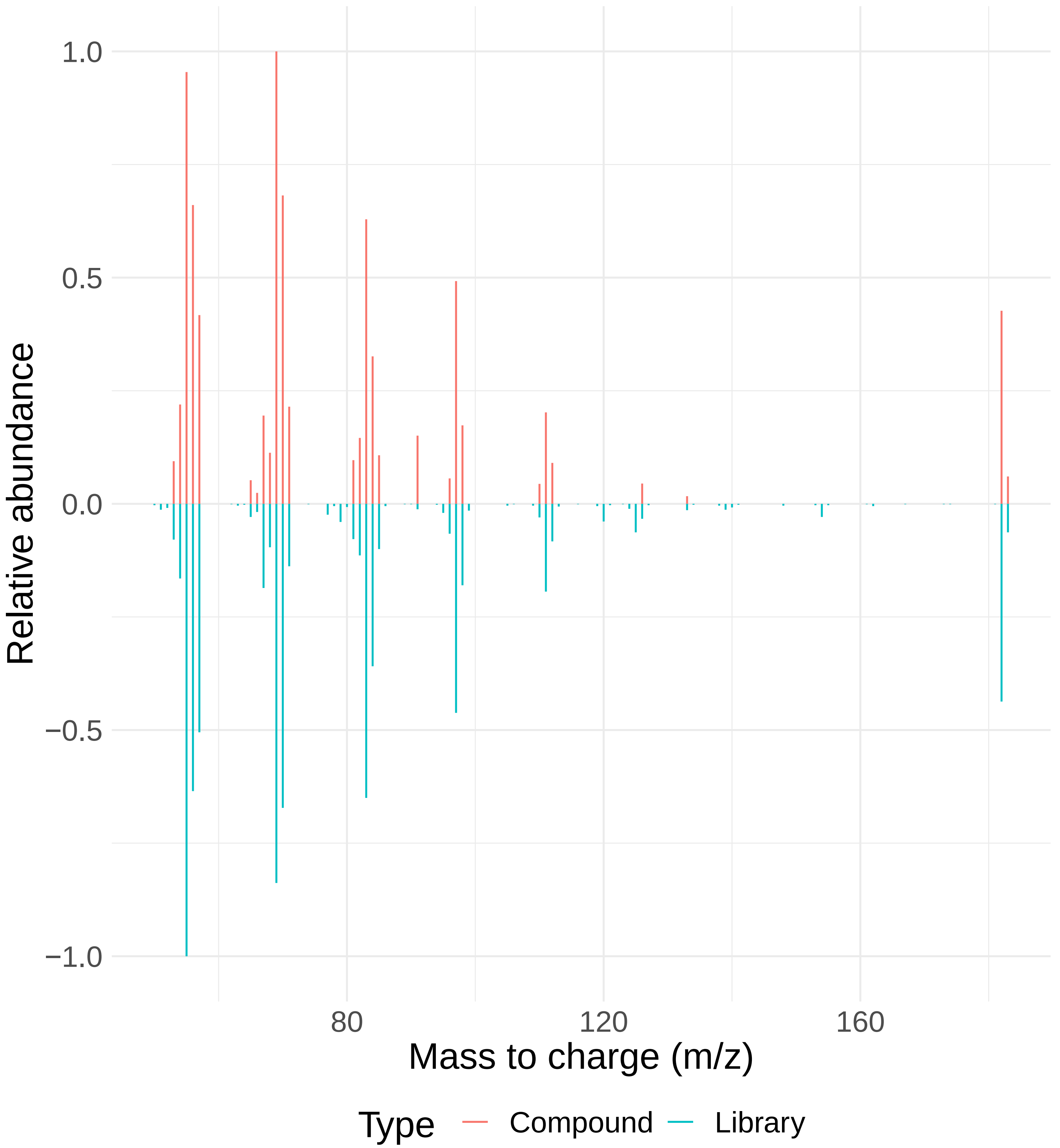

n−Tridecane

HS−SPME−GC−MS

splitless

ID: 21

RI: 1302

PubChem CID: 12388

https://pubchem.ncbi.nlm.nih.gov/compound/12388

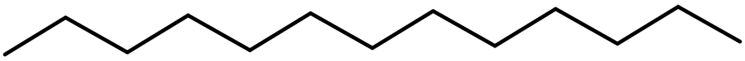

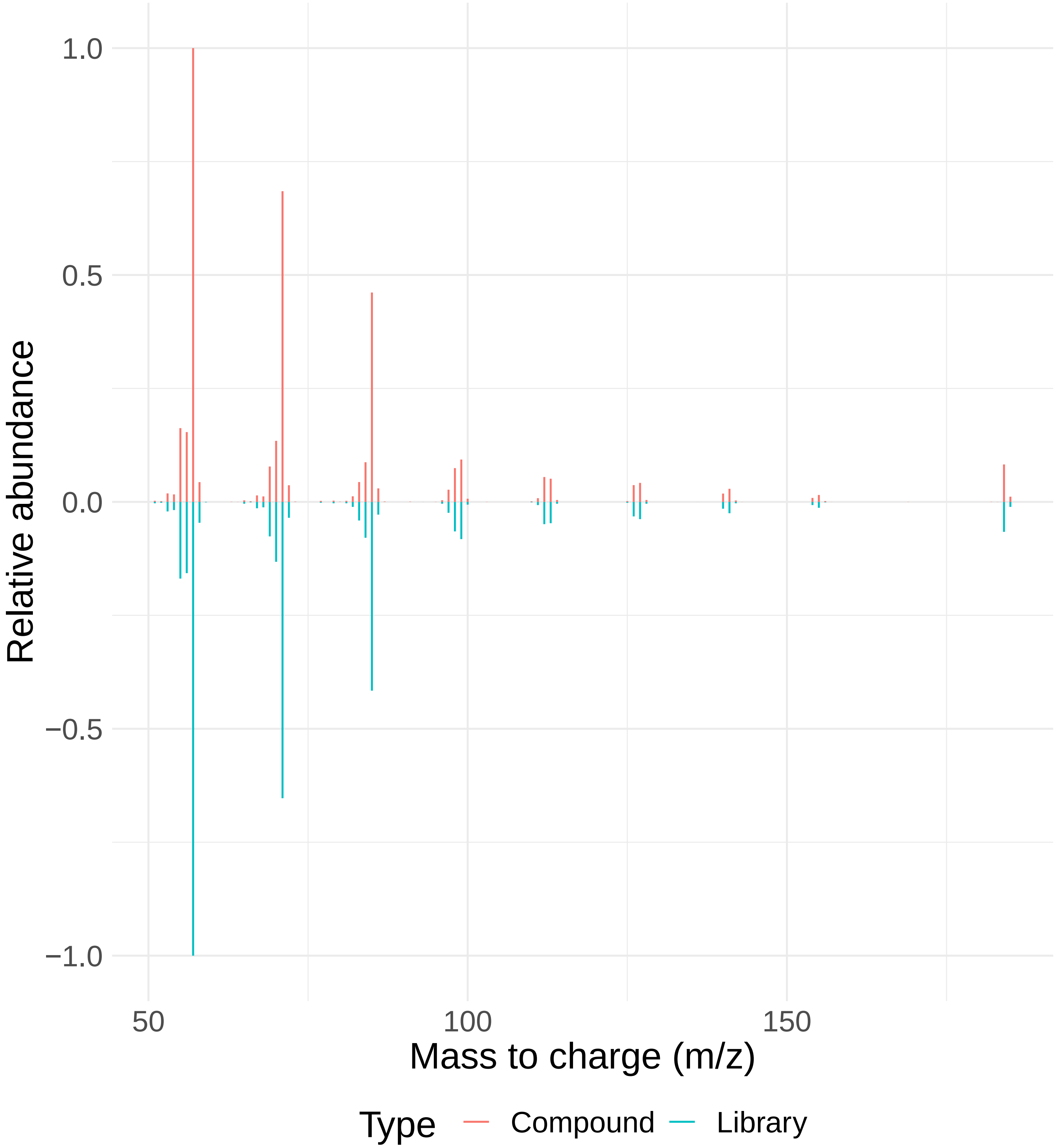

1H−indole

Liquid−injection−GC−MS

splitless

ID: 22

RI: 1309

PubChem CID: 798

https://pubchem.ncbi.nlm.nih.gov/compound/798

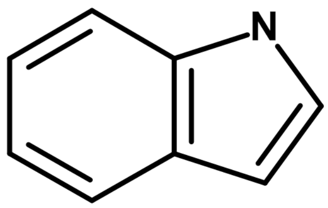

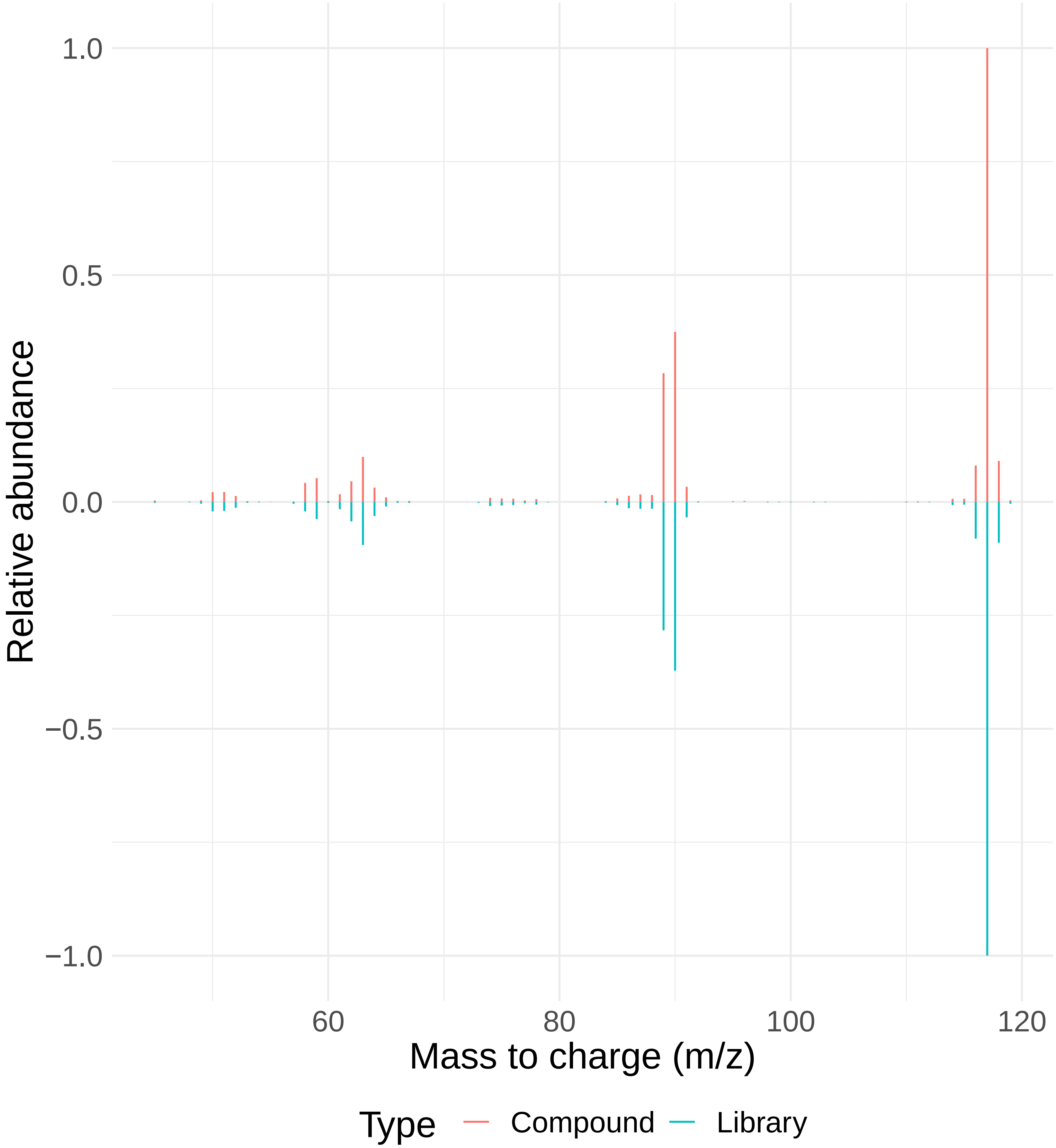

(E)−3,7−Dimethylocta−2,6−dienoic acid

Liquid−injection−GC−MS

splitless

ID: 23

RI: 1358

PubChem CID: 5275520

https://pubchem.ncbi.nlm.nih.gov/compound/5275520

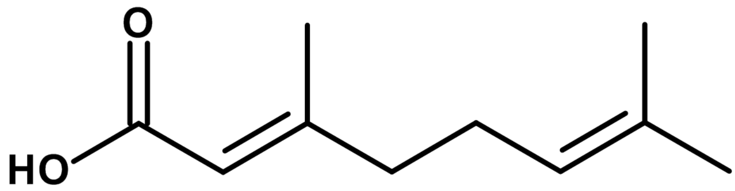

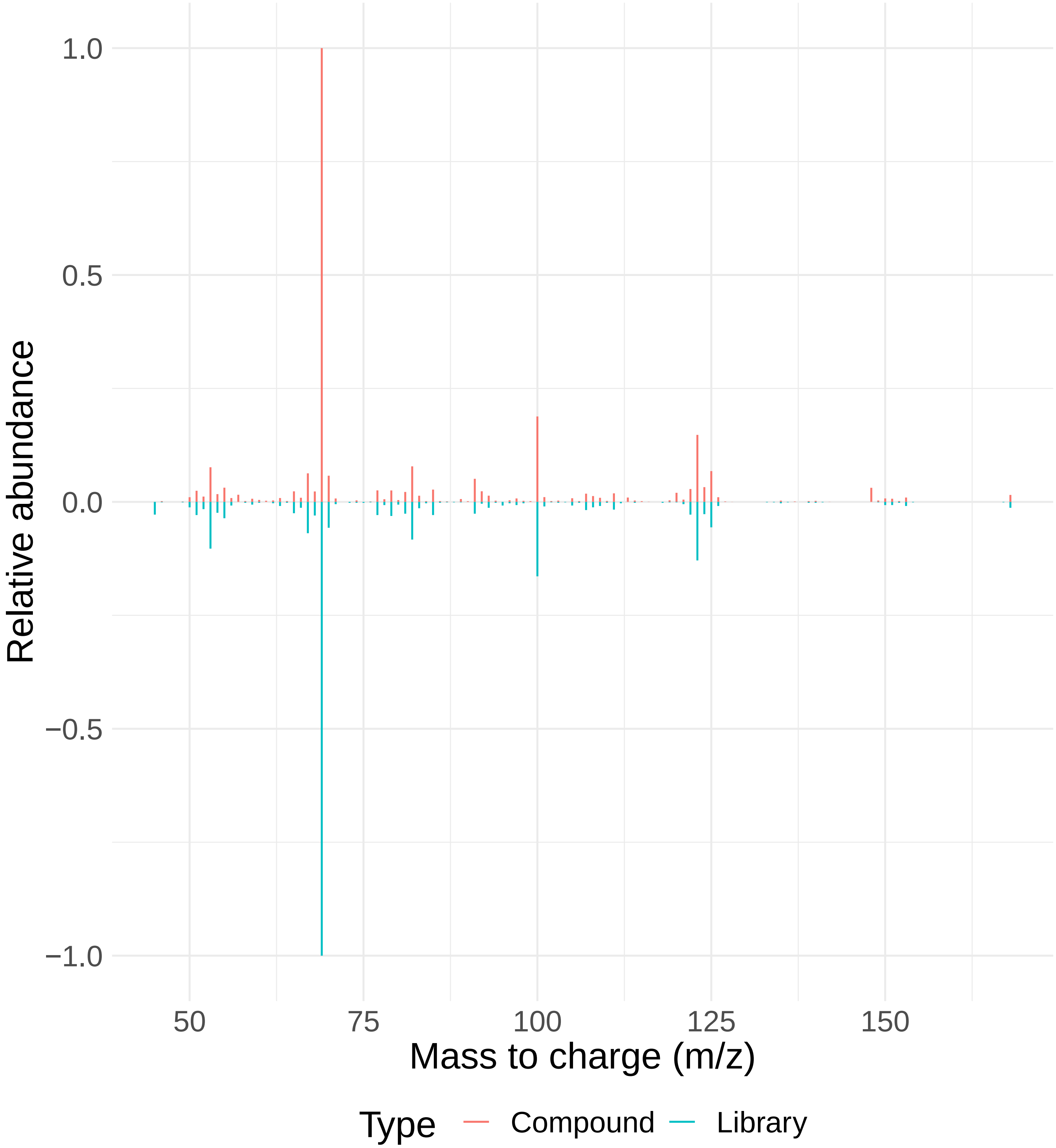

5−Methylbenzene−1,3−diol

Liquid−injection−GC−MS

splitless

ID: 24

RI: 1373

PubChem CID: 10436

https://pubchem.ncbi.nlm.nih.gov/compound/10436

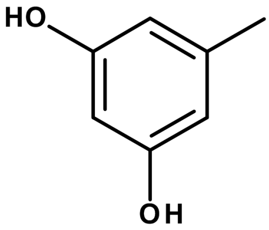

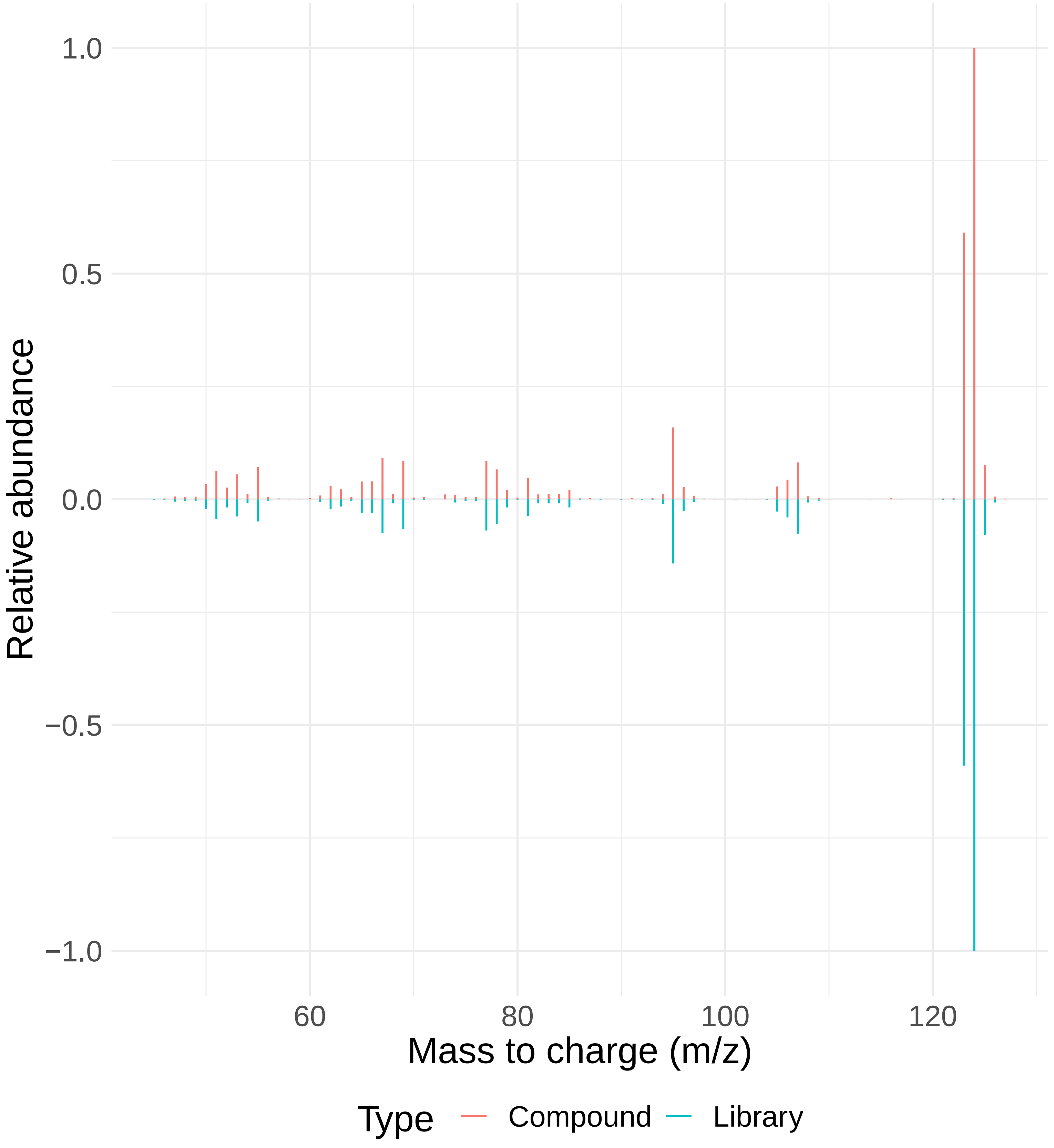

3−Acetyl−6−methylpyran−2,4−dione

Liquid−injection−GC−MS

splitless

ID: 25

RI: 1382

PubChem CID: 122903

https://pubchem.ncbi.nlm.nih.gov/compound/122903

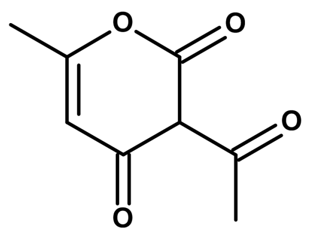

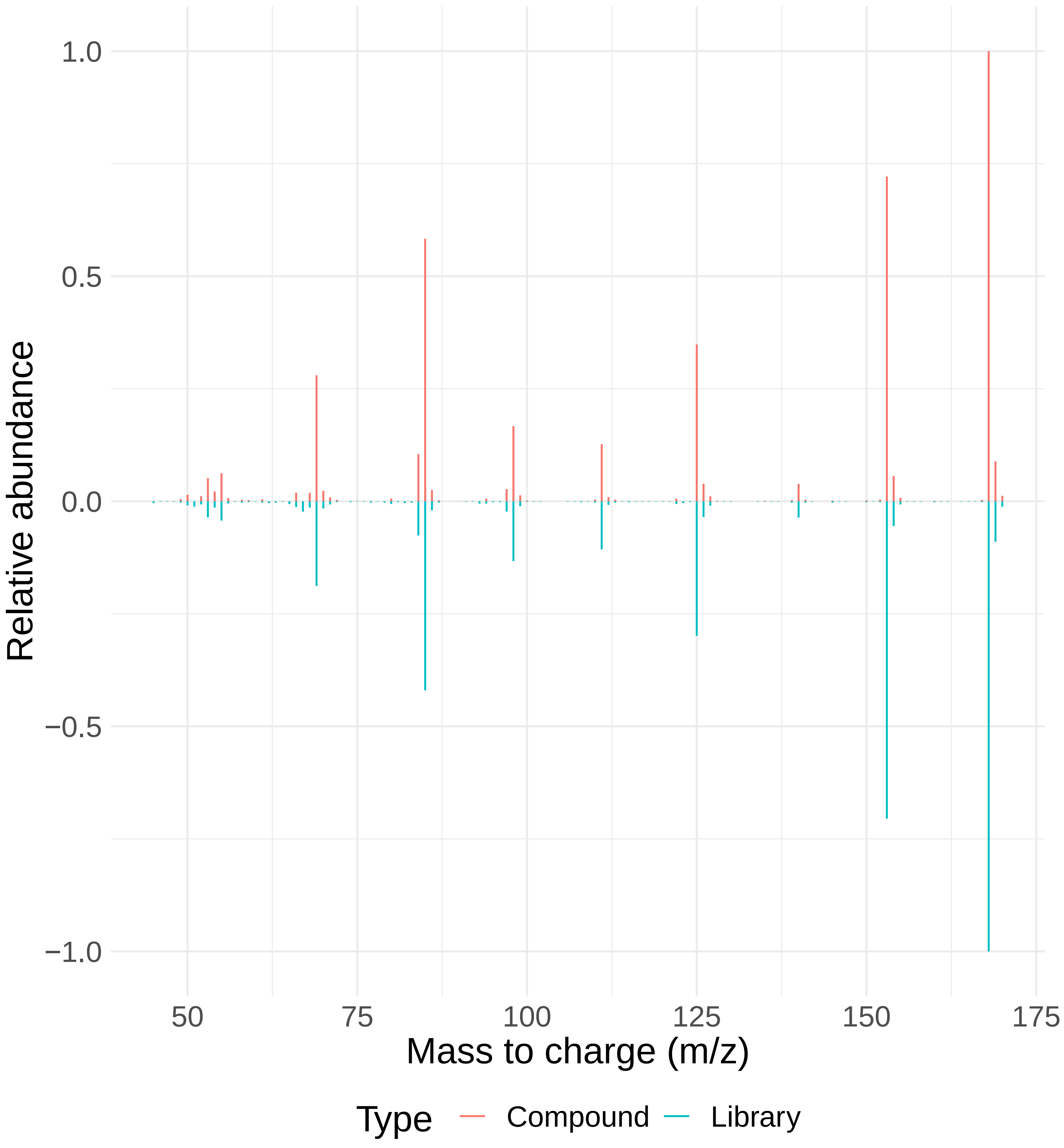

1−(2−hydroxy−4,5−dimethylphenyl)ethanone

HS−SPME−GC−MS

splitless

ID: 26

RI: 1444

PubChem CID: 118976

https://pubchem.ncbi.nlm.nih.gov/compound/118976

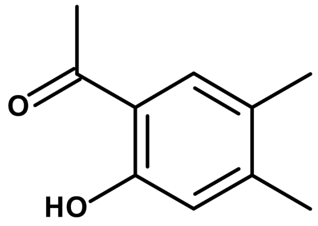

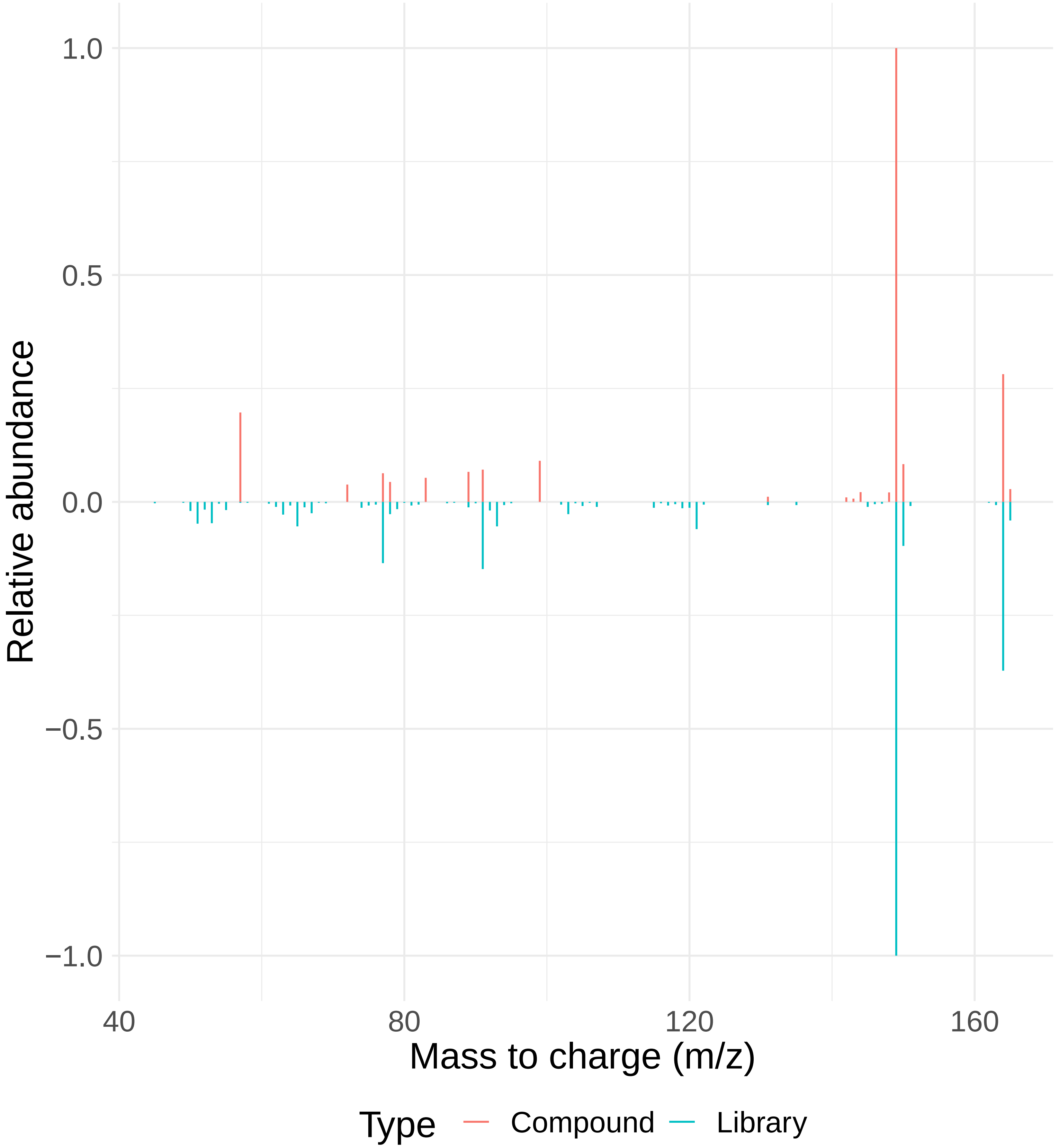

4−Hydroxy−6−methylpyran−2−one

Liquid−injection−GC−MS

split 10:1

ID: 27

RI: 1436

PubChem CID: 54675757

https://pubchem.ncbi.nlm.nih.gov/compound/54675757

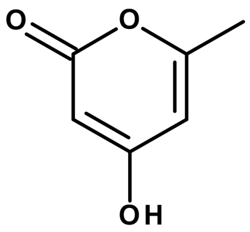

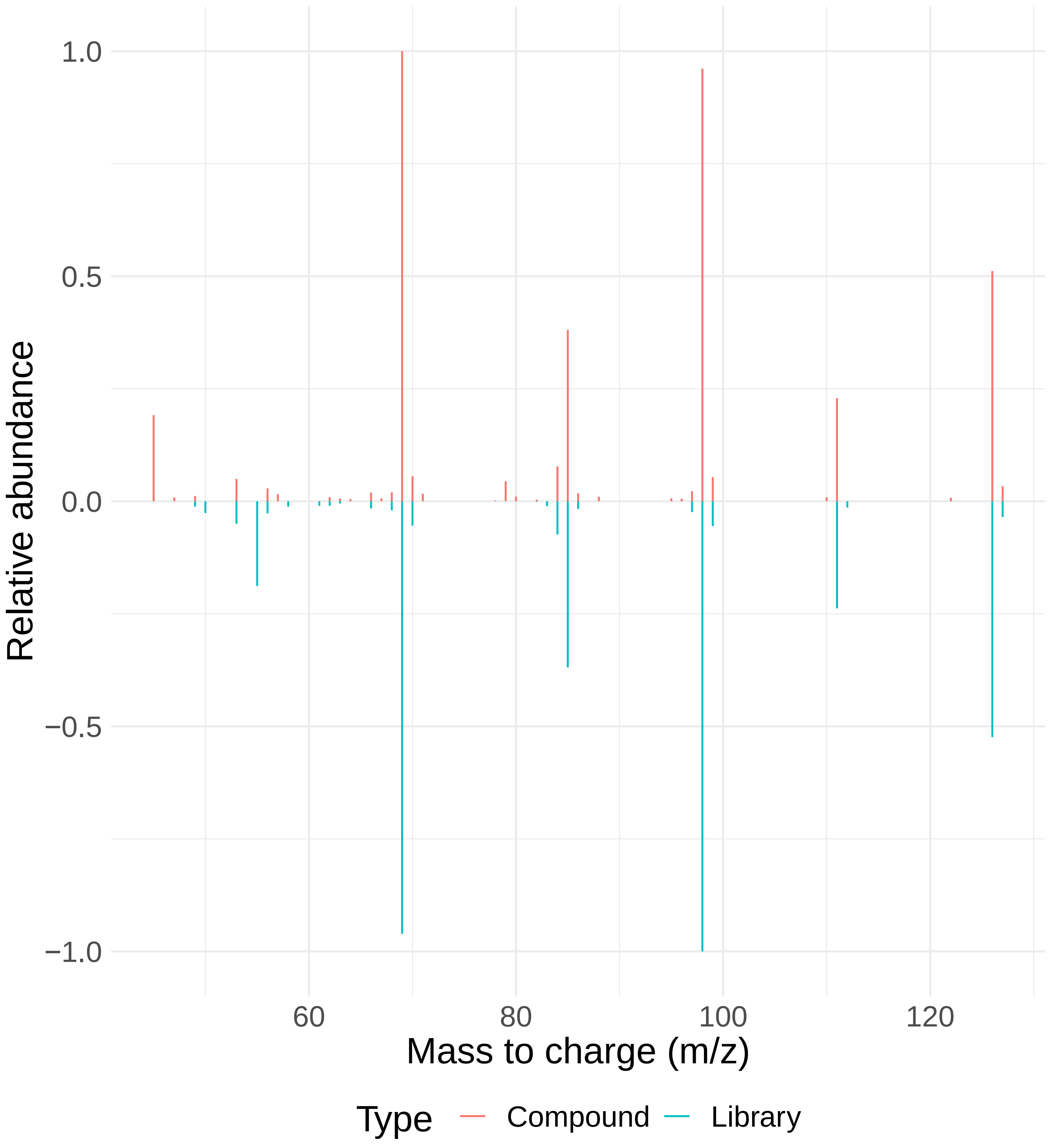

Pentadec−1−ene

HS−SPME−GC−MS

splitless

ID: 28

RI: 1492

PubChem CID: 25913

https://pubchem.ncbi.nlm.nih.gov/compound/25913

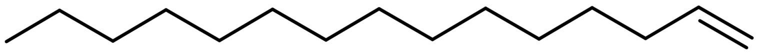

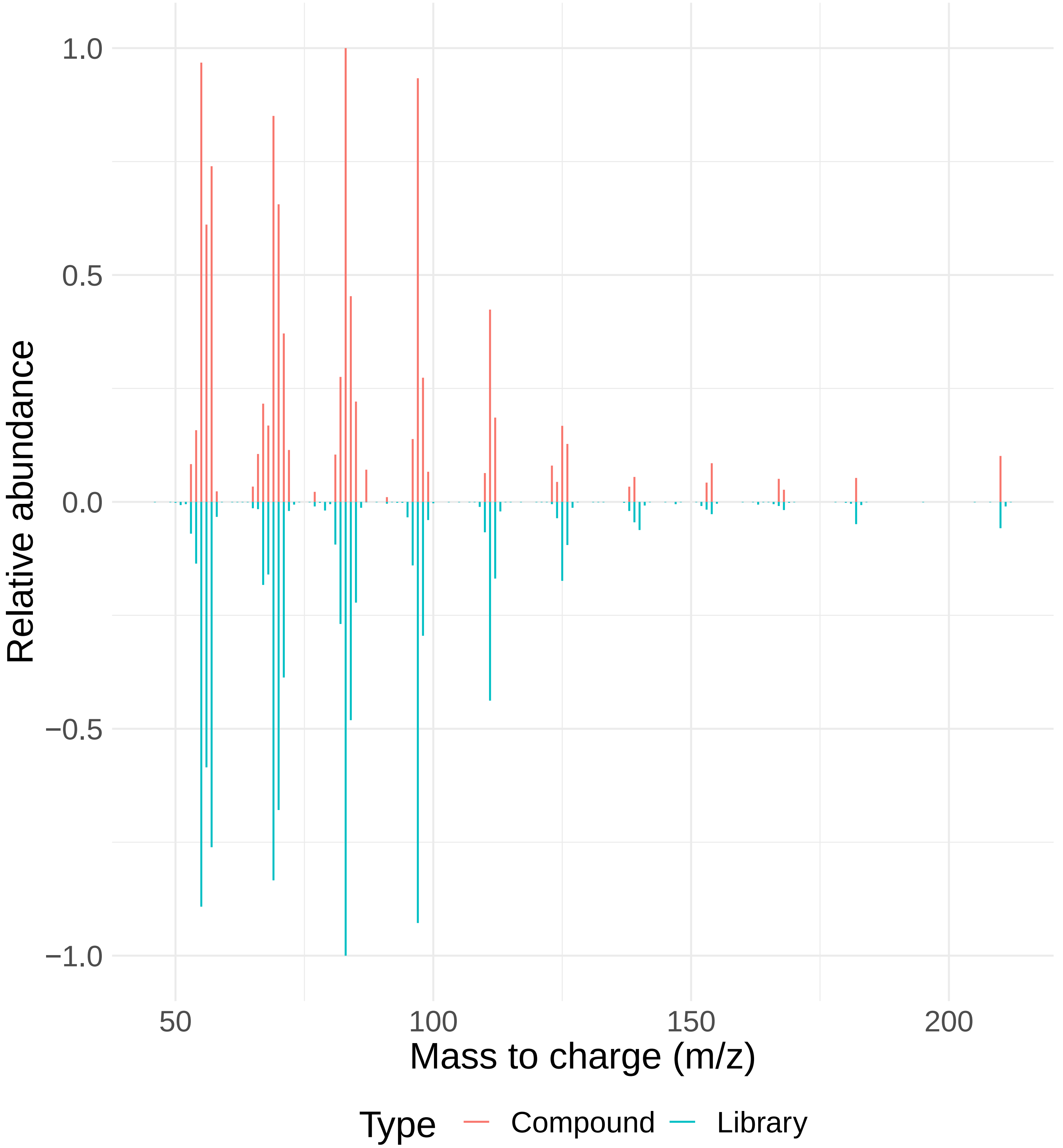

n−Pentadecane

HS−SPME−GC−MS

splitless

ID: 29

RI: 1502

PubChem CID: 12391

https://pubchem.ncbi.nlm.nih.gov/compound/12391

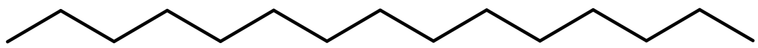

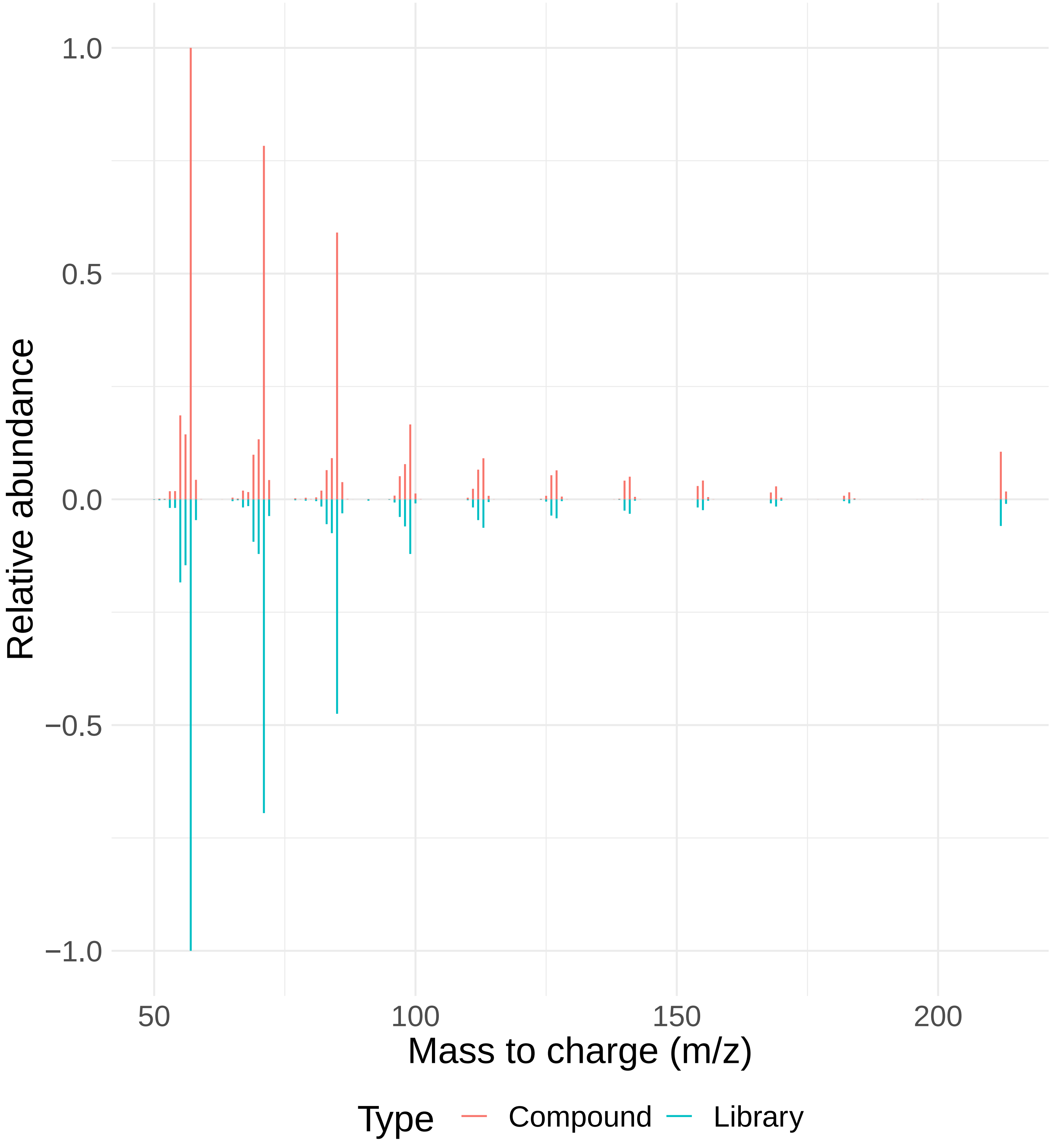

Benzene−1,3,5−triol

Liquid−injection−GC−MS

split 10:1

ID: 30

RI: 1593

PubChem CID: 359

https://pubchem.ncbi.nlm.nih.gov/compound/359

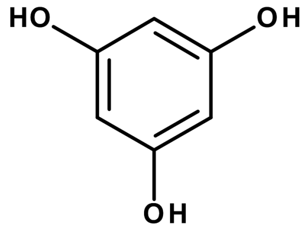

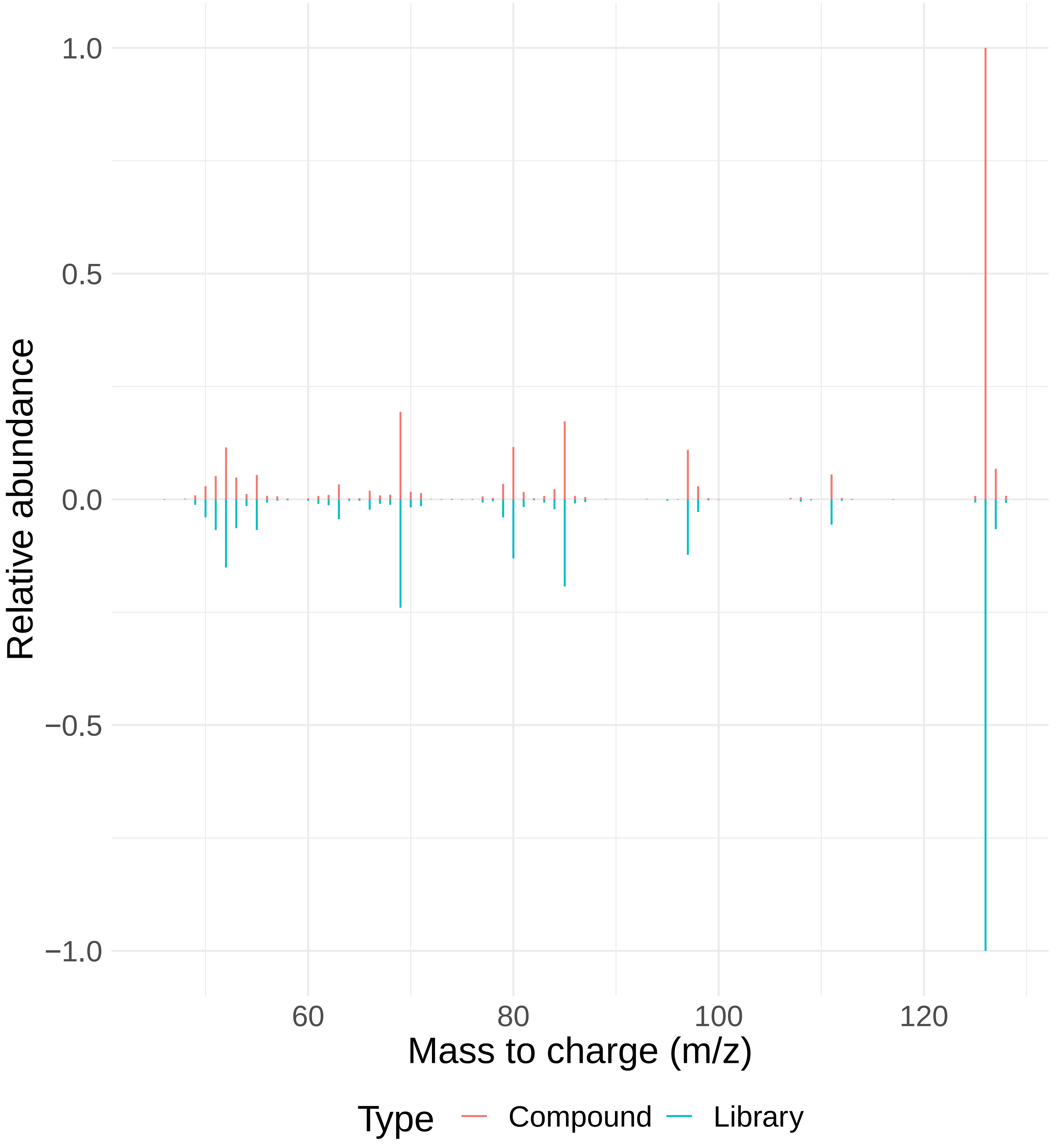

n−Hexadecane

HS−SPME−GC−MS

splitless

ID: 31

RI: 1601

PubChem CID: 11006

https://pubchem.ncbi.nlm.nih.gov/compound/11006

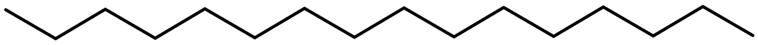

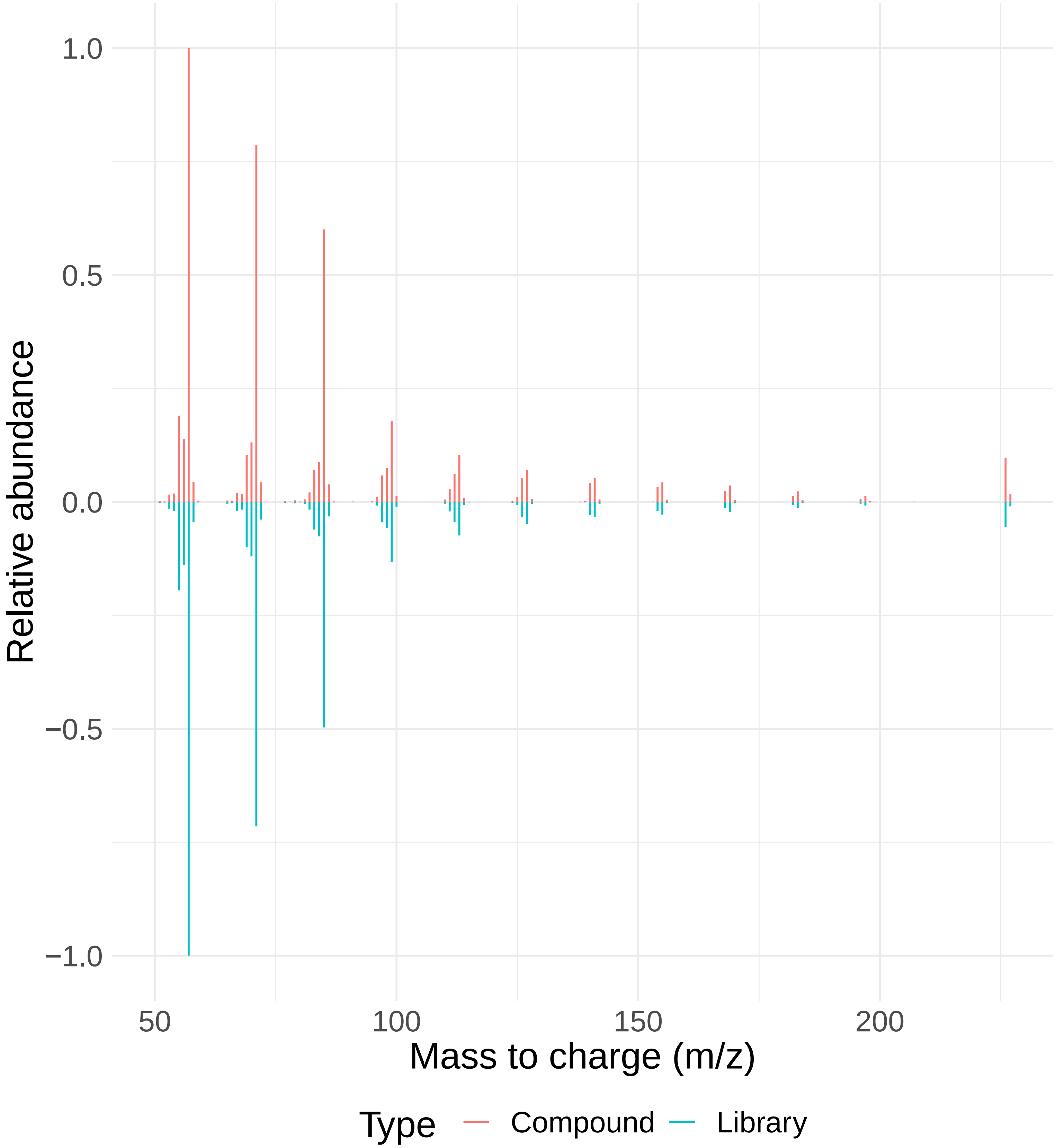

Heptadec−8−ene

HS−SPME−GC−MS

splitless

ID: 32

RI: 1680

PubChem CID: 5364555

https://pubchem.ncbi.nlm.nih.gov/compound/5364555

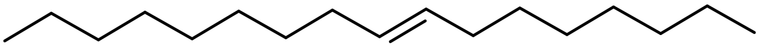

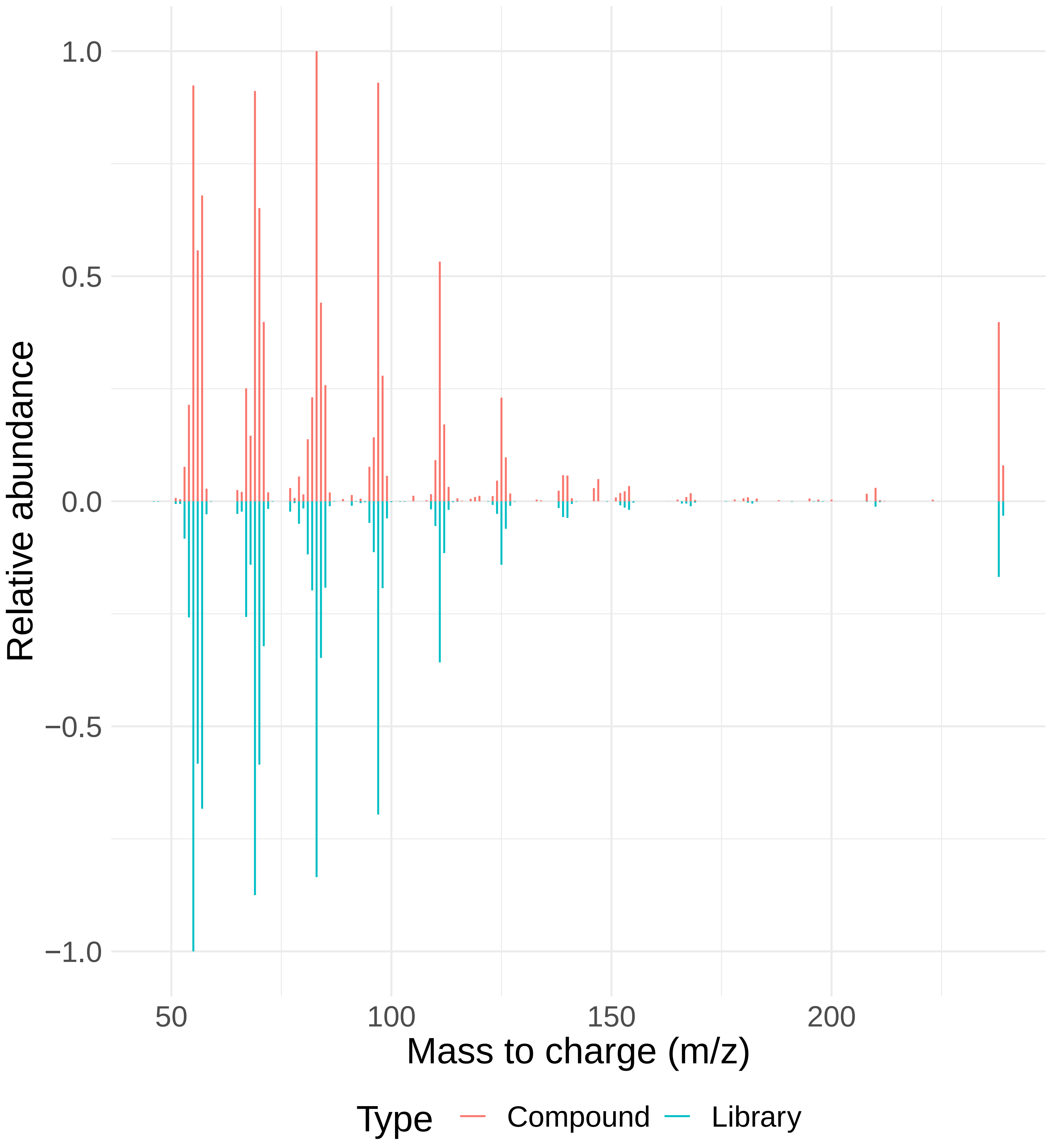

Heptadec−1−ene

HS−SPME−GC−MS

splitless

ID: 33

RI: 1693

PubChem CID: 23217

https://pubchem.ncbi.nlm.nih.gov/compound/23217

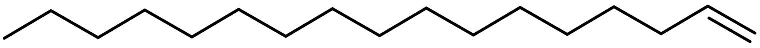

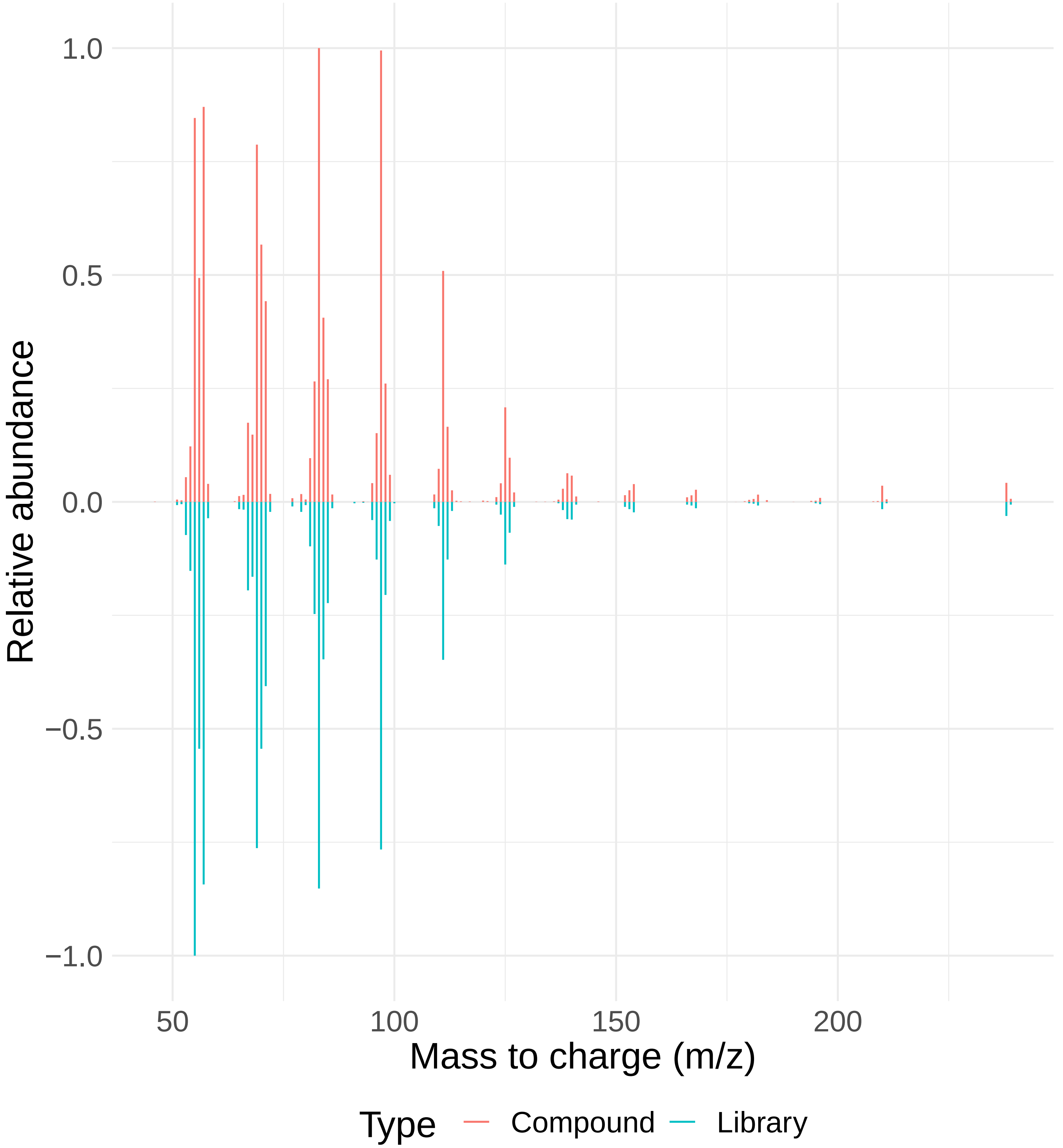

n−Heptadecane

HS−SPME−GC−MS

splitless

ID: 34

RI: 1704

PubChem CID: 12398

https://pubchem.ncbi.nlm.nih.gov/compound/12398

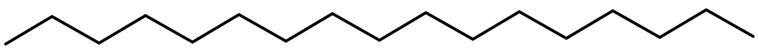

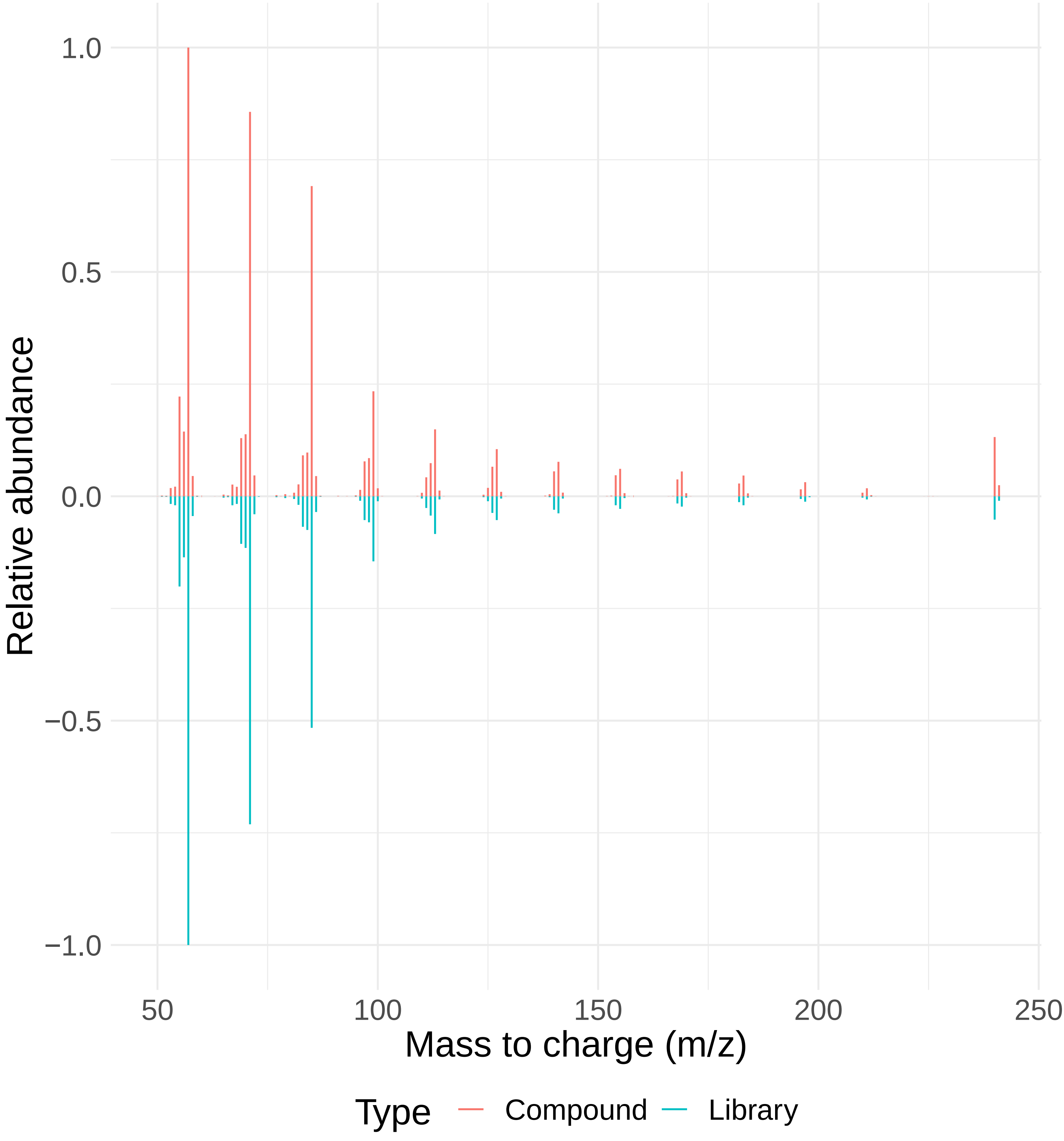

n−Octadecane

HS−SPME−GC−MS

splitless

ID: 35

RI: 1800

PubChem CID: 11635

https://pubchem.ncbi.nlm.nih.gov/compound/11635

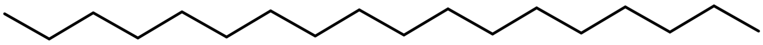

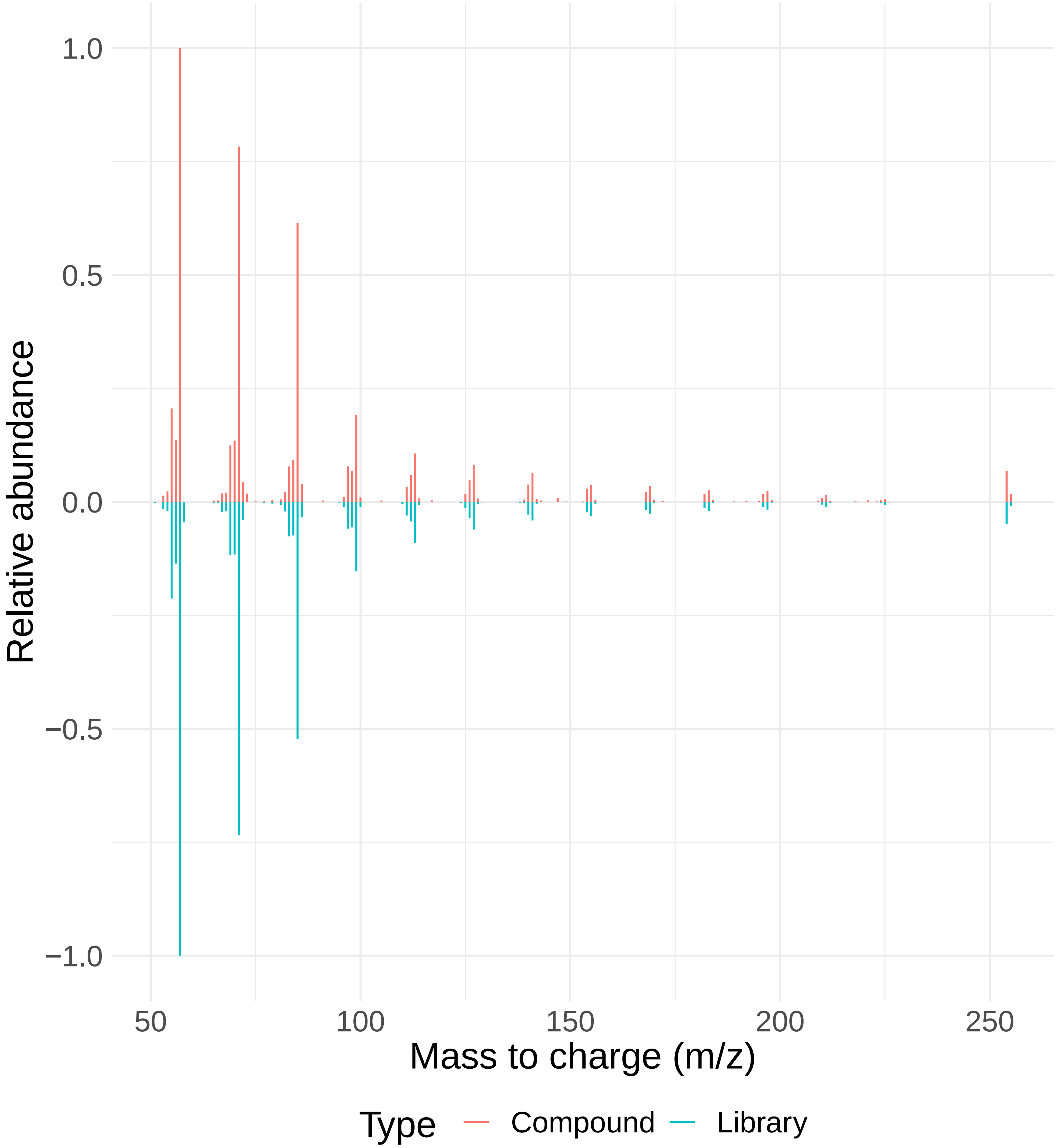

Hexadecan−1−al

Liquid−injection−GC−MS

splitless

ID: 36

RI: 1822

PubChem CID: 984

https://pubchem.ncbi.nlm.nih.gov/compound/984

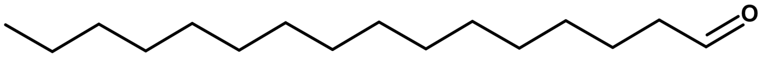

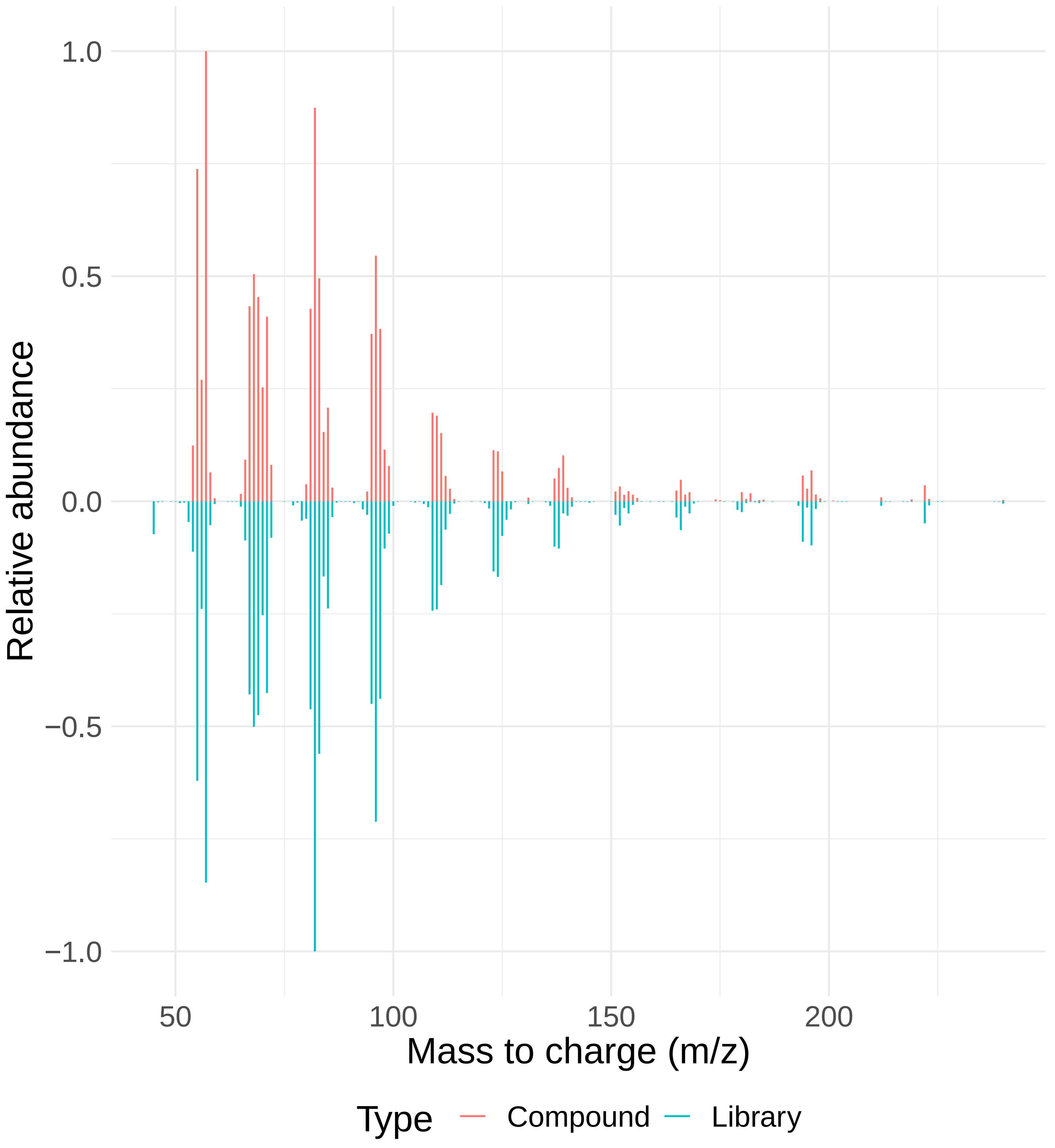

Hexadecan−1−ol

HS−SPME−GC−MS

splitless

ID: 37

RI: 1882

PubChem CID: 2682

https://pubchem.ncbi.nlm.nih.gov/compound/2682

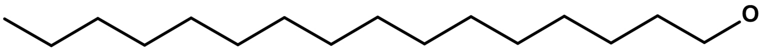

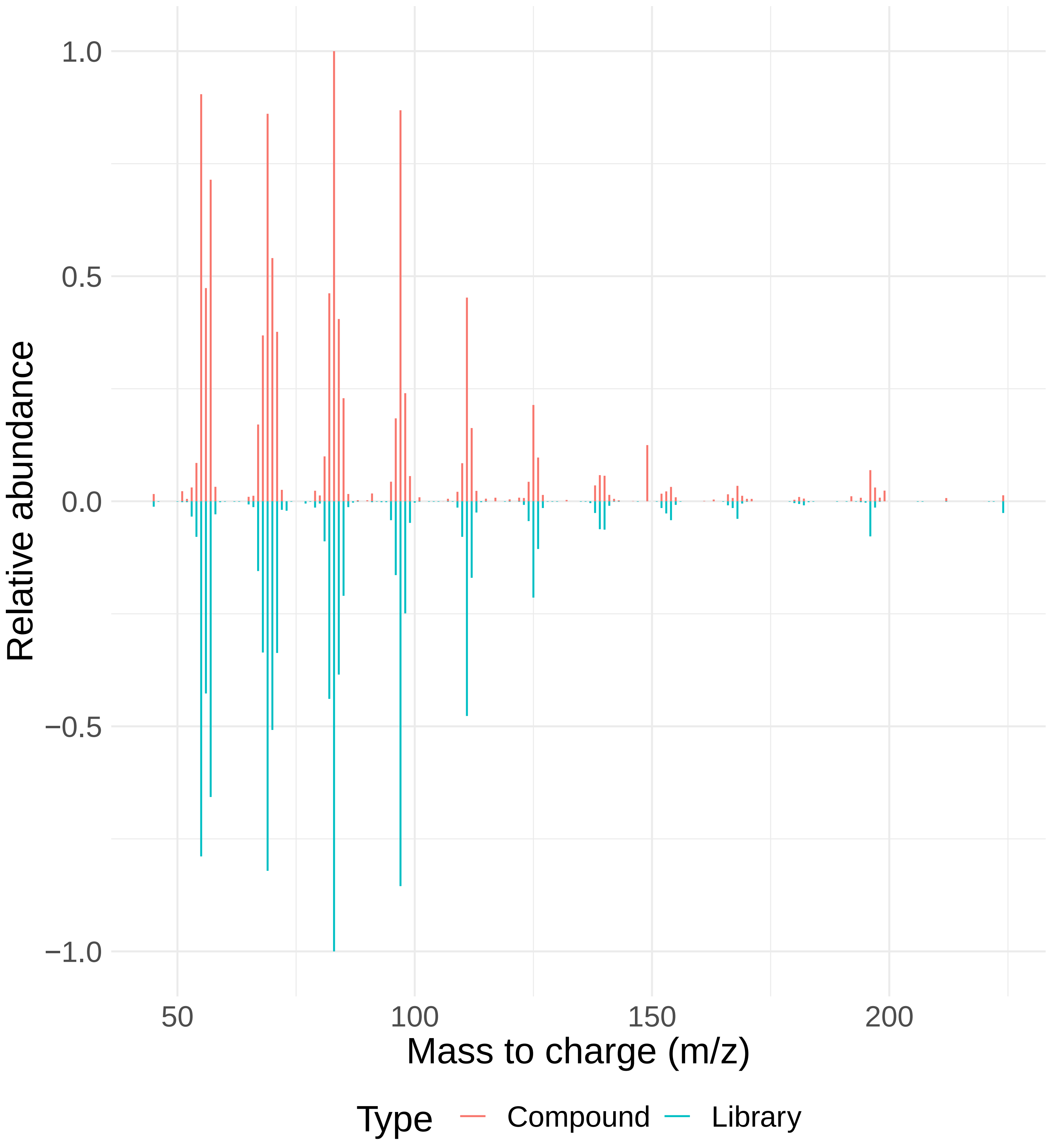

1−(2,4,6−Trihydroxyphenyl)ethanone

Liquid−injection−GC−MS

split 60:1

ID: 38

RI: 1883

PubChem CID: 68073

https://pubchem.ncbi.nlm.nih.gov/compound/68073

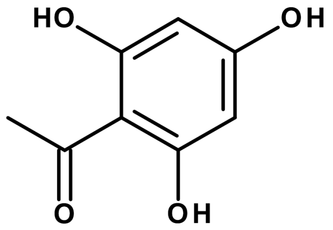

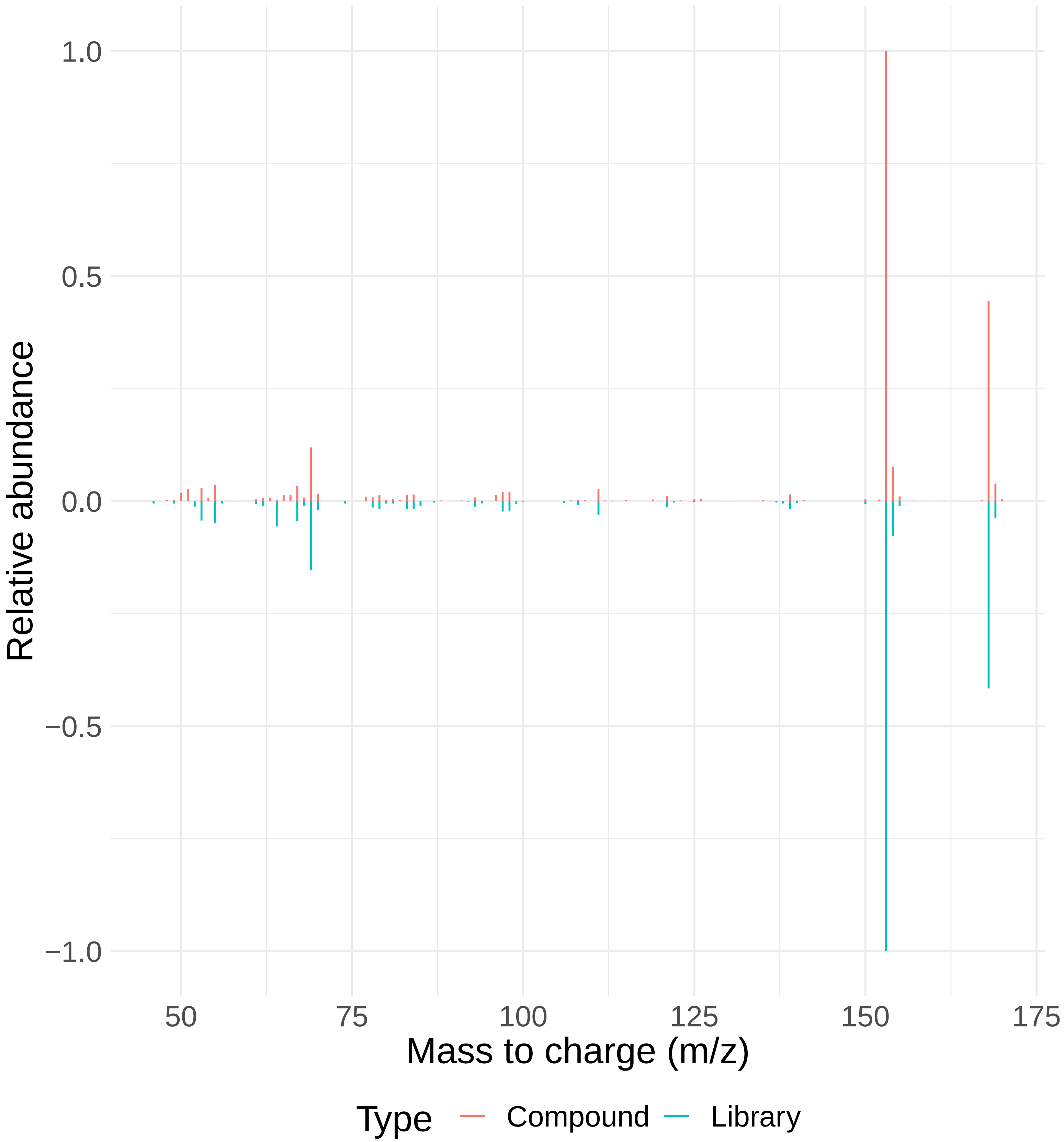

n−Nonadecane

HS−SPME−GC−MS

splitless

ID: 39

RI: 1900

PubChem CID: 12401

https://pubchem.ncbi.nlm.nih.gov/compound/12401

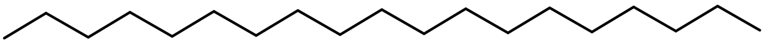

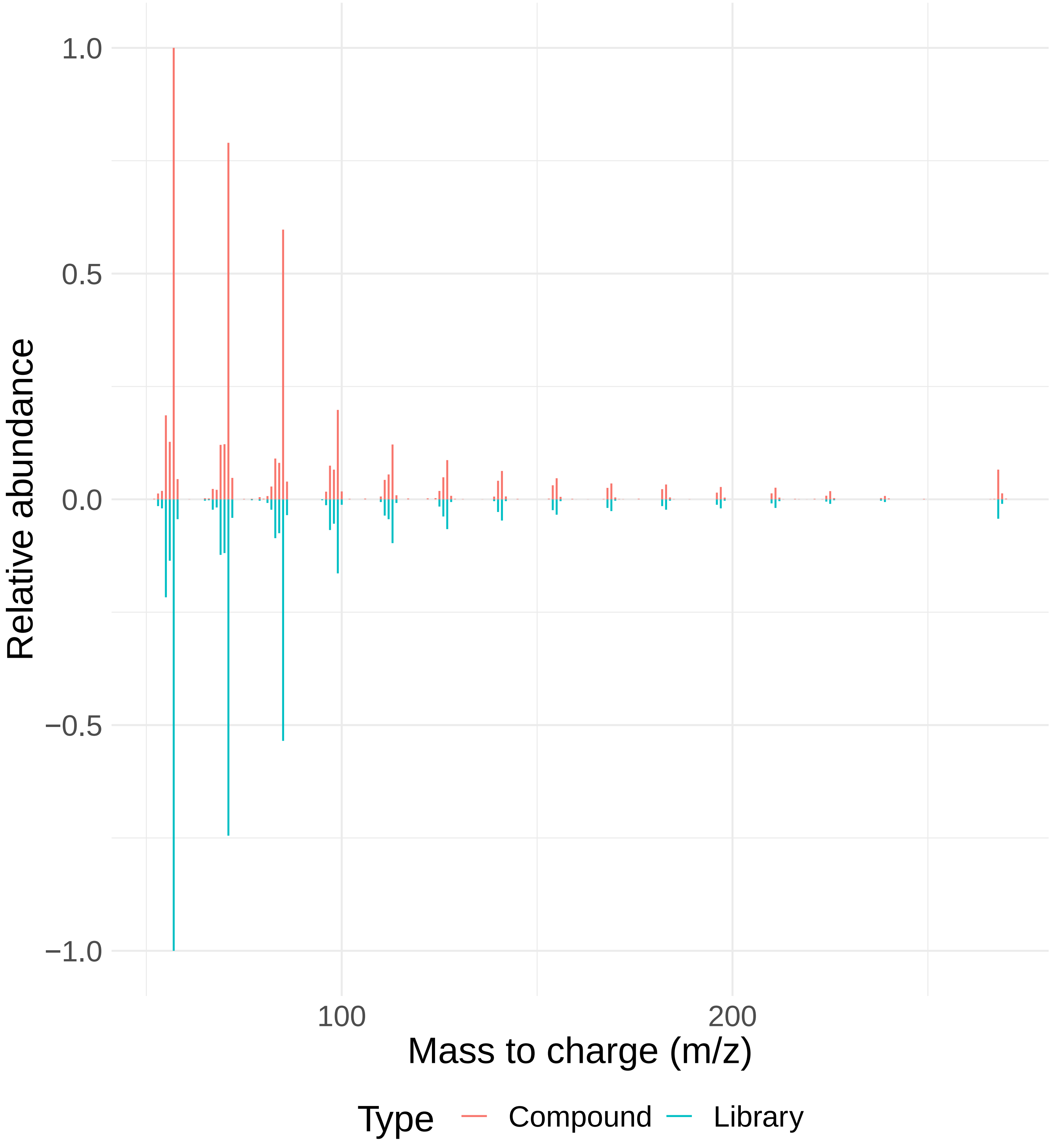

Heptadecan−2−one or isomer

HS−SPME−GC−MS

splitless

ID: 40

RI: 1905

PubChem CID: 18027

https://pubchem.ncbi.nlm.nih.gov/compound/18027

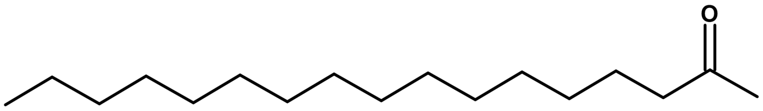

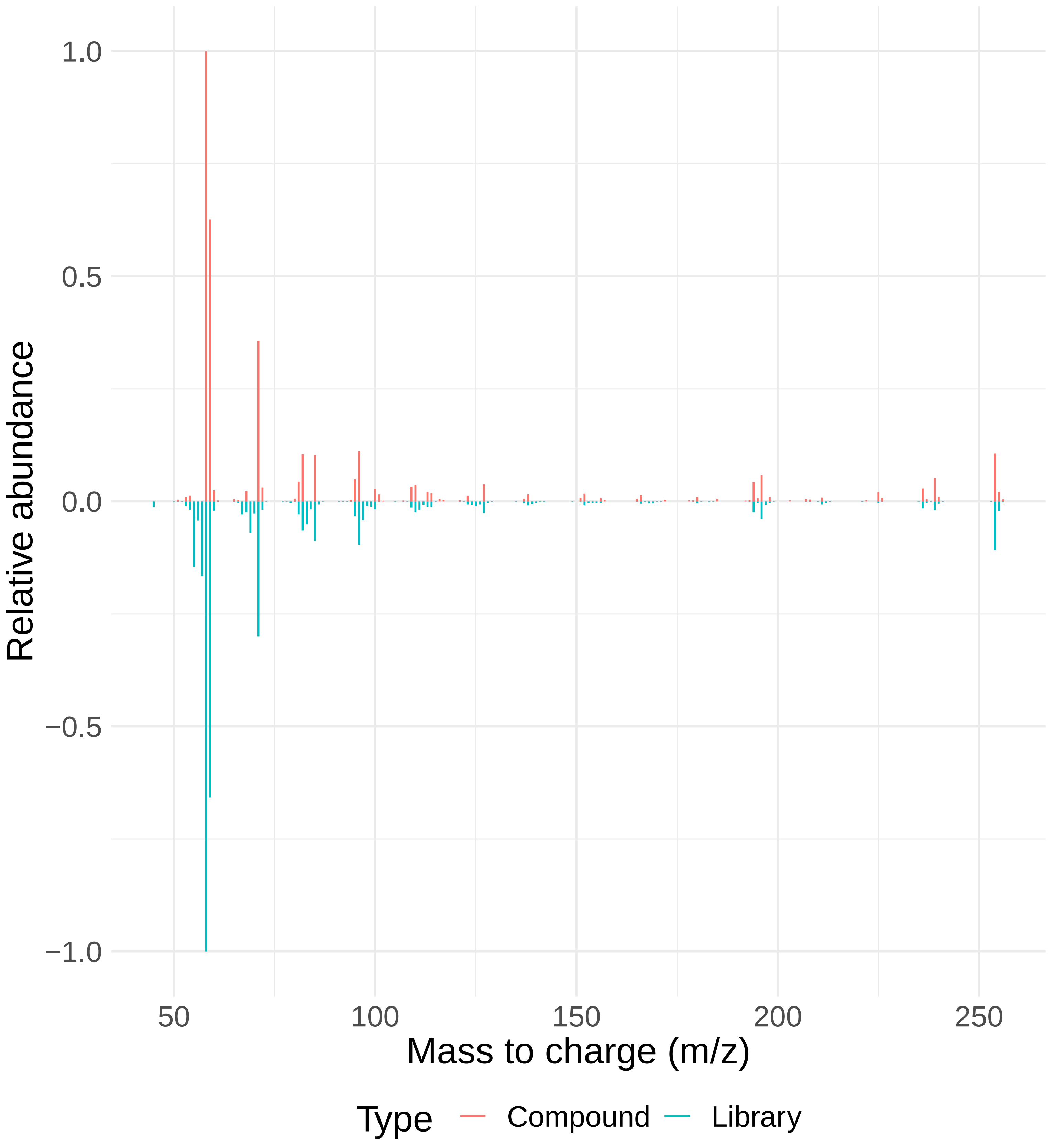

5,7−Dihydroxy−2−methylchromen−4−one

Liquid−injection−GC−MS

split 60:1

ID: 41

RI: 2000

PubChem CID: 5375252

https://pubchem.ncbi.nlm.nih.gov/compound/5375252

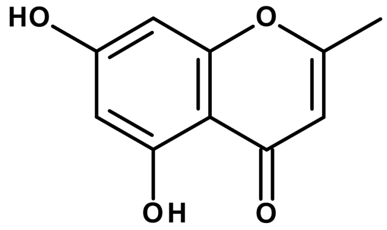

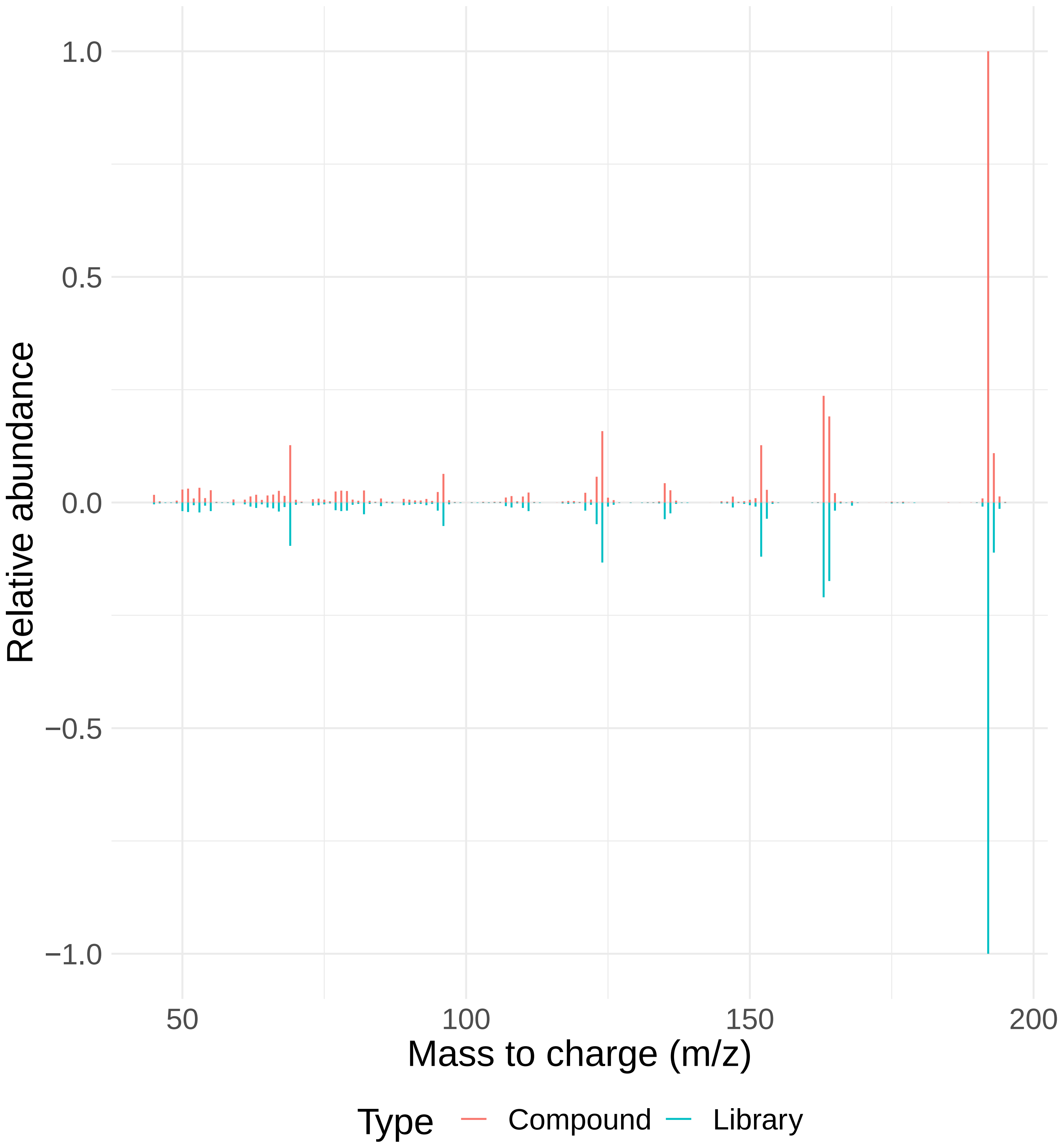

1−(3−Acetyl−2,4,6−trihydroxyphenyl)ethanone

Liquid−injection−GC−MS

split 10:1

ID: 42

RI: 2016

PubChem CID: 16547

https://pubchem.ncbi.nlm.nih.gov/compound/16547

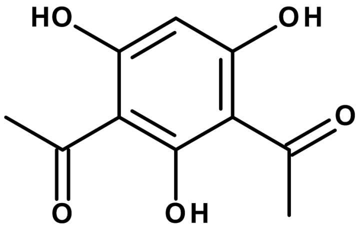

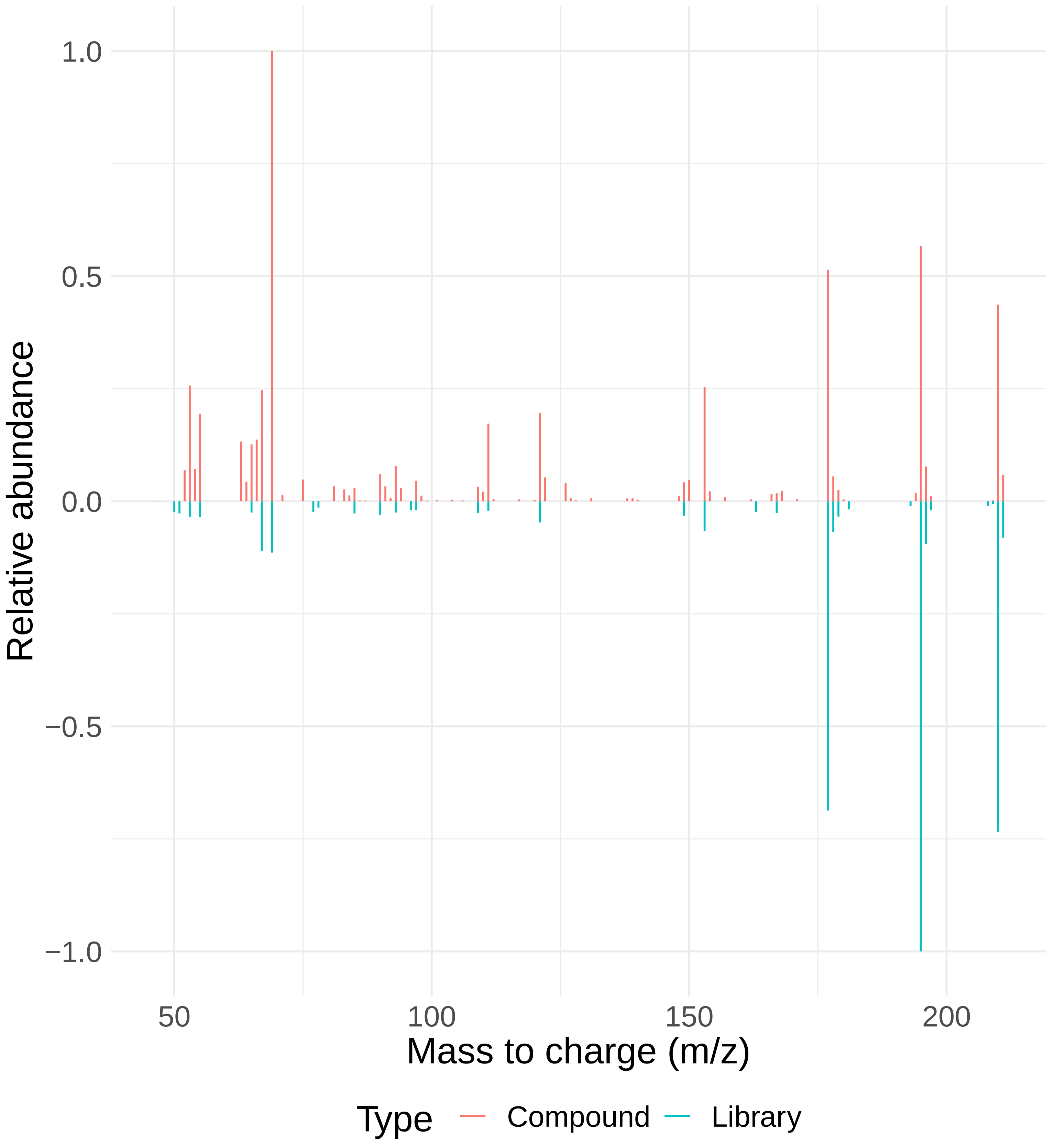

Octadecan−1−ol

HS−SPME−GC−MS

splitless

ID: 43

RI: 2089

PubChem CID: 8221

https://pubchem.ncbi.nlm.nih.gov/compound/8221

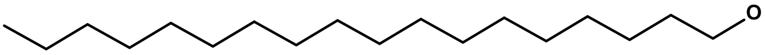

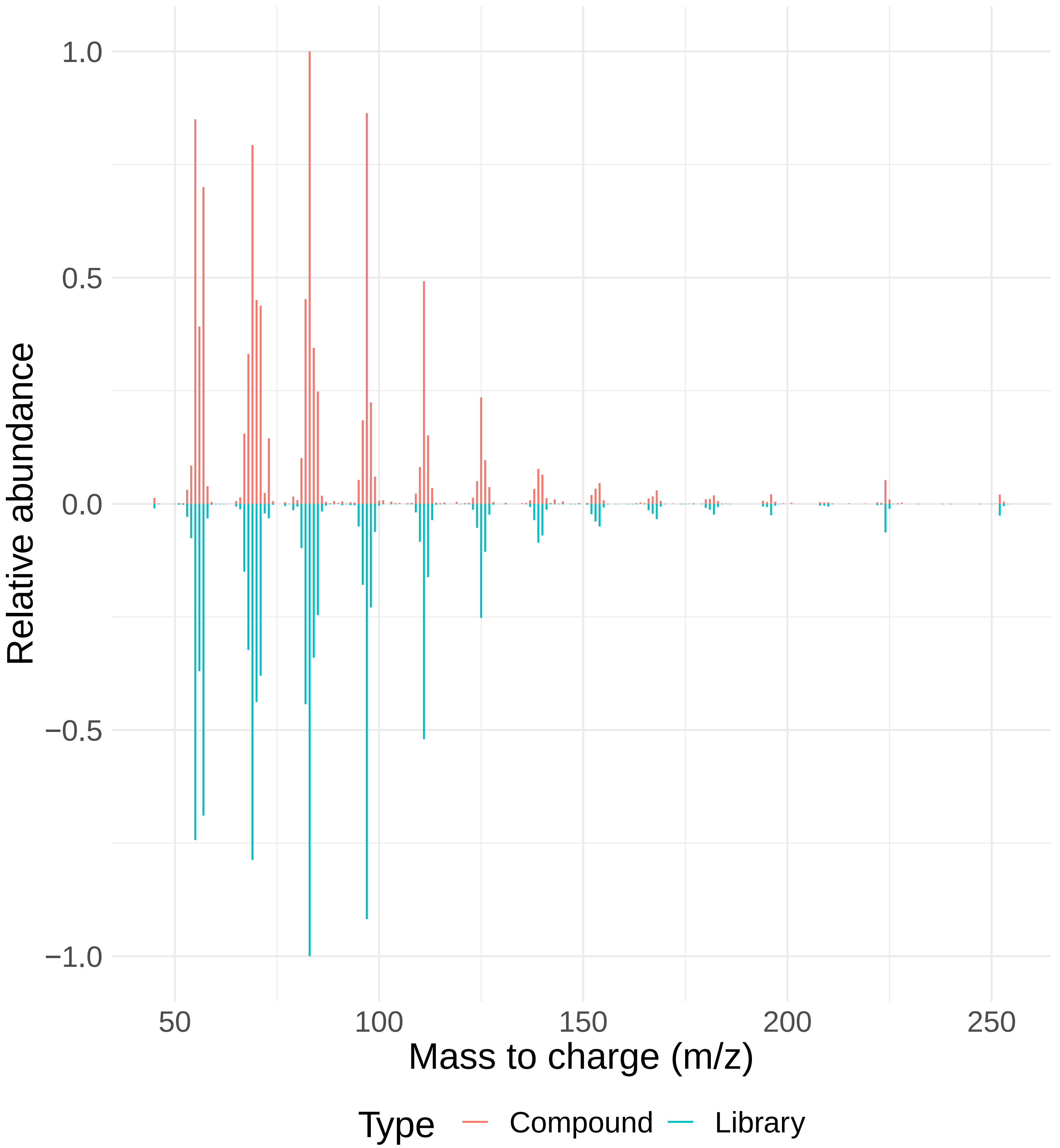

(Z)−Octadec−9−enoic acid

Liquid−injection−GC−MS

splitless

ID: 44

RI: 2146

PubChem CID: 445639

https://pubchem.ncbi.nlm.nih.gov/compound/445639

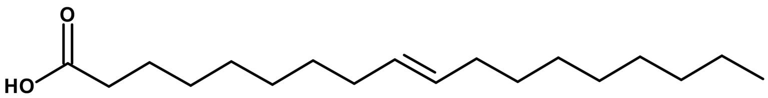

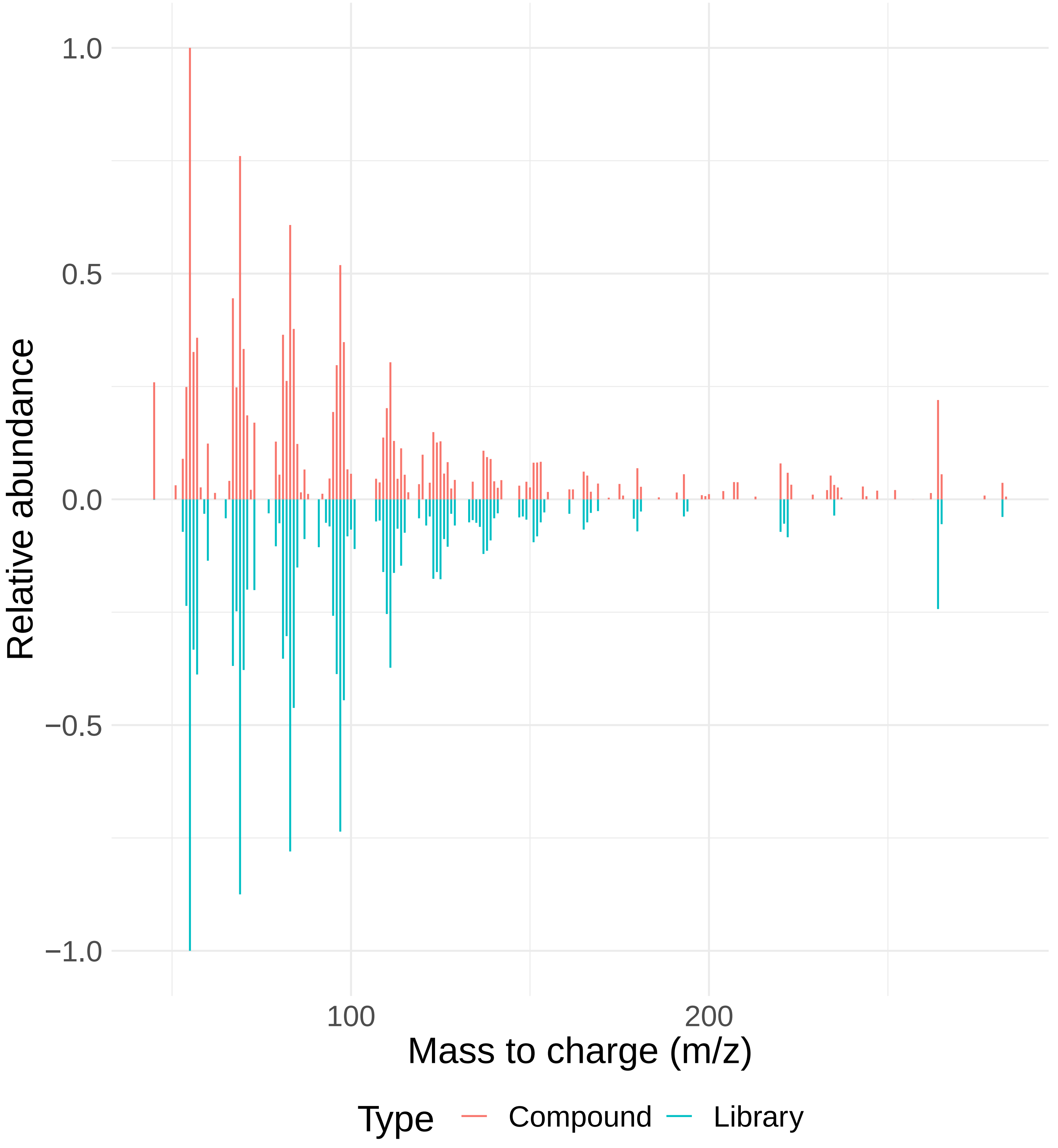

Octadecanoic acid or isomer

HS−SPME−GC−MS

splitless

ID: 45

RI: 2167

PubChem CID: 5281

https://pubchem.ncbi.nlm.nih.gov/compound/5281

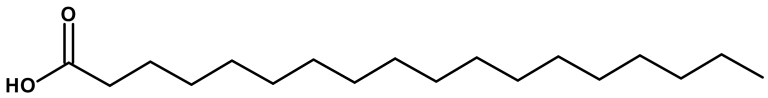

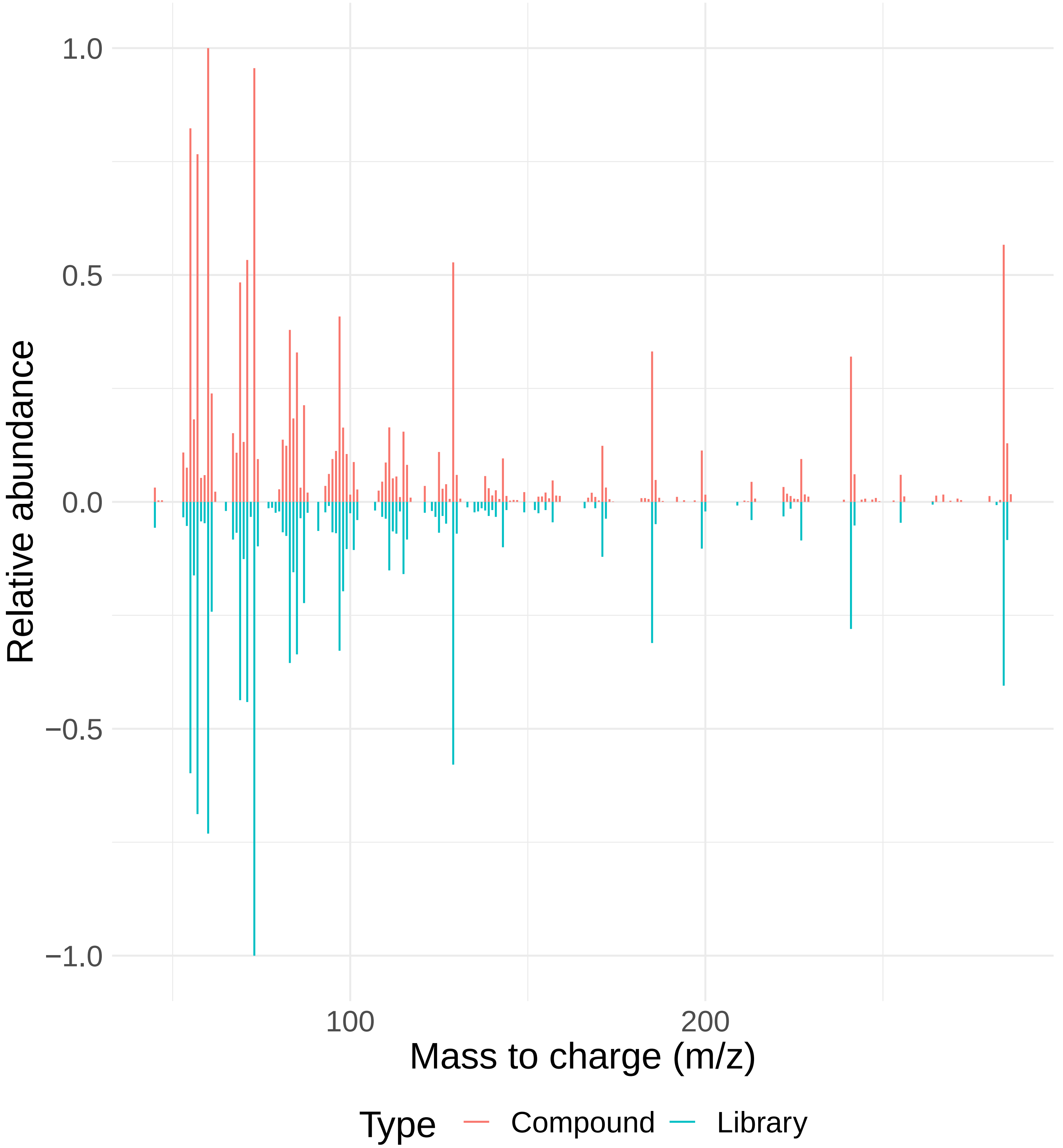

Supplement: Supplementary file 1 [file molecules-24-03468-s001.zip › Compounds.pdf]
