# Supplementary material for: Volatiles from the Mandibular Gland Reservoir Content of Colobopsis explodens Laciny and Zettel, 2018, Worker Ants (Hymenoptera: Formicidae)
Source: Molecules. 2019 Sep 24;24(19):3468. doi: 10.3390/molecules24193468 (PMC6804081; doi:10.3390/molecules24193468)
Supplement: Supplementary file 1 [file molecules-24-03468-s001.zip › Supplementals_revised.pdf]

SUPPLEMENTARY MATERIAL

TO THE ARTICLE

Volatiles from the Mandibular Gland Reservoir Content of *Colobopsis explodens* Laciny and Zettel, 2018 Worker Ants (Hymenoptera: Formicidae)

Michaela Hoenigsberger [1], Alexey G Kopchinskiy [2], Christoph Bueschl [1], Alexandra Parich [1], Alice Laciny [3], Herbert Zettel [3], Kamariah A Salim [4], Linda BL Lim [5], Irina S Druzhinina [2,6] and Rainer Schuhmacher [1,*]

[1] Institute of Bioanalytics and Agro-Metabolomics (iBAM), Department of Agrobiotechnology (IFA-Tulln), University of Natural Resources and Life Sciences, Vienna (BOKU), Konrad-Lorenz-Strasse 20, A-3430 Tulln, Austria; michaela.fischer@boku.ac.at (M.H.); christoph.bueschl@boku.ac.at (C.B.); alexandra.parich@boku.ac.at (A.P.); rainer.schuhmacher@boku.ac.at (R.S.)

[2] Institute of Chemical, Environmental and Bioscience Engineering (ICEBE), TU Wien, Gumpendorferstrasse 1a, A-1060 Vienna, Austria; alexey.kopchinskiy@tuwien.ac.at (A.G. K.); irina.druzhinina@tuwien.ac.at or irina.druzhinina@njau.edu.cn (I.S.D.)

[3] 2nd Zoological Department, Natural History Museum Vienna, Burgring 7, A-1010 Vienna, Austria; alice.laciny@nhm-wien.ac.at (A.L.); herbert.zettel@nhm-wien.ac.at (H.Z.)

[4] Environmental and Life Sciences, Faculty of Science, Universiti Brunei Darussalam, Jalan Tungku Gadong BE1410, Brunei Darussalam; udhl_2003@yahoo.com (K.A.S.)

[5] Chemical Sciences, Faculty of Science, Universiti Brunei Darussalam, Jalan Tungku Link, Gadong BE1410, Brunei Darussalam; linda.lim@ubd.edu.bn (L.B.L.L.)

[6] Fungal Genomics Group, College of Resources and Environmental Sciences, Nanjing Agricultural University, Weigang NO. 1, Nanjing 210095, China

* Correspondence: rainer.schuhmacher@boku.ac.at; Tel.: +43-1-47654-97307

Table of Contents

Table S1. *C. explodens* samples analyzed in this study. 2

Table S2. Overall similarity scores for identified or annotated VOCs in single sample files as obtained by MetaboliteDetector data evaluation. 3

Table S3. Identified or annotated VOCs in the MGRC of *C. explodens* already reported from ants or other insects in the literature. 5

Table S4. Data matrix used for statistical analysis of VOCs identified or annotated after liquid-injection-GC-MS. 7

Table S1. *C. explodens* samples analyzed in this study.

				HS-SPME-GC-MS			Liquid-injection-GC-MS		
**Designation**	**Sampled**	**#Ants collected [1]**	**#Ants used [2]**	**MGRCs [3]**	**Samples [4]**	**Weight [mg] [5]**	**MGRCs [3]**	**Samples [6]**	**Weight [mg] [5]**
Cexpl_5/2014	May 2014	300	70	50	2	17.7-20.8	20	2	6.92-7.14
Cexpl_4/2015	April 2015	1000	155	125	5	17.2-21-7	30	3	6.23-7.26
Cexpl_11/2015	Nov 2015	1000	155	125	5	19.4-26.5	30	3	7.01-8.02

All *C. explodens* minor workers originated from the main colony located next to the Kuala Belalong Field Studies Centre [1].

[1] Total number of ants collected on each field trip.

[2] Number of ants from which the MGRCs for GC-MS analysis were obtained. These ants were randomly chosen from the collection sampled on the respective field trip.

[3] Total number of ants from which the MGRC was obtained.

[4] Each analytical sample (12 in total) consisted of pooled MGRCs isolated from 25 ants.

[5] Weight range of analytical sample obtained from the respective sample batch.

[6] Each analytical sample (8 in total) consisted of pooled MGRCs isolated from 10 ants.

Table S2. Overall similarity scores for identified or annotated VOCs in single sample files as obtained by MetaboliteDetector data evaluation.

	**HS-SPME-GC-MS**														**Liquid-injection-GC-MS**									
	**Cexpl_5/2014**		**Cexpl_4/2015**					**Cexpl_11/2015**							**Cexpl_5/2014**		**Cexpl_4/2015**			**Cexpl_11/2015**				
**ID[1]**	**1**	**2**	**1**	**2**	**3**	**4**	**5**	**1**	**2**	**3**	**4**	**5**	**#ID**	**ID [%]**	**1**	**2**	**1**	**2**	**3**	**1**	**2**	**3**	**#ID**	**ID [%]**
**1**	0.97	0.98	0.96	0.94	0.94	0.95	0.94	0.94	0.94	0.94	0.94	0.94	12	100	n.d.	n.d.	n.d.	n.d.	n.d.	n.d.	n.d.	n.d.	<4	<50
**2**	1.00	1.00	1.00	0.98	0.99	0.98	0.99	0.98	0.98	0.98	0.96	0.98	12	100	n.d.	n.d.	n.d.	n.d.	n.d.	n.d.	n.d.	n.d.	<4	<50
**3***	0.93	0.93	0.94	0.94	0.93	0.93	0.93	0.94	0.94	0.93	0.94	0.94	12	100	0.91	0.97	0.98	0.98	0.95	0.79	0.95	0.95	7	88
**4**	0.90	0.94	0.98	0.98	1.00	0.99	0.99	1.00	1.00	0.99	1.00	0.99	12	100	0.93	0.94	0.97	0.99	0.98	0.95	0.90	0.92	8	100
**5**	0.96	n.d.	0.94	0.88	0.81	0.90	0.94	0.93	0.97	0.87	0.95	0.93	8	67	n.d.	n.d.	n.d.	n.d.	n.d.	n.d.	n.d.	n.d.	<4	<50
**6***	n.d.	n.d.	n.d.	n.d.	n.d.	n.d.	n.d.	n.d.	n.d.	n.d.	n.d.	n.d.	<6	<50	0.96	0.98	0.75	0.96	0.96	0.93	0.94	0.78	6	75
**7***	n.d.	n.d.	n.d.	n.d.	n.d.	n.d.	n.d.	n.d.	n.d.	n.d.	n.d.	n.d.	<6	<50	0.98	0.96	0.99	0.96	0.96	0.82	n.d.	0.98	6	75
**8**	0.99	0.96	0.96	0.96	0.96	0.96	0.96	0.95	0.96	0.96	0.95	0.96	12	100	0.95	0.94	0.98	0.92	0.85	0.74	0.92	0.94	6	75
**9**	0.91	0.96	0.99	0.98	0.90	0.97	0.92	0.97	0.90	0.84	0.95	0.97	11	92	0.91	0.91	0.98	0.97	0.97	0.97	0.89	0.93	7	88
**10**	0.96	0.99	0.83	0.84	0.92	0.83	0.89	0.90	0.92	0.94	0.78	0.92	7	58	1.00	0.77	0.92	0.90	0.74	0.57	0.79	0.85	3	38
**11**	0.99	0.99	0.98	0.98	0.96	0.99	0.98	0.99	0.98	0.98	0.99	0.98	12	100	0.97	0.94	0.99	0.99	0.98	0.92	0.97	0.93	8	100
**12**	0.99	0.92	0.59	0.64	0.85	0.69	0.89	0.98	0.96	0.94	0.99	0.97	7	58	0.99	1.00	1.00	1.00	1.00	1.00	1.00	1.00	8	100
**13**	n.d.	0.98	0.80	0.86	0.98	0.89	0.90	0.94	0.95	0.91	0.95	0.96	8	67	n.d.	n.d.	n.d.	n.d.	n.d.	n.d.	n.d.	n.d.	<4	<50
**14**	0.95	0.44	0.91	0.92	0.95	0.95	0.94	0.97	0.99	0.97	0.98	0.98	11	92	n.d.	n.d.	n.d.	n.d.	n.d.	n.d.	n.d.	n.d.	<4	<50
**15**	n.d.	n.d.	n.d.	n.d.	n.d.	n.d.	n.d.	n.d.	n.d.	n.d.	n.d.	n.d.	<6	<50	0.97	0.96	0.98	0.99	0.98	n.d.	n.d.	n.d.	5	63
**16**	0.95	0.99	0.92	0.94	0.92	0.95	0.96	0.88	0.89	0.90	0.81	0.87	8	67	0.92	0.84	0.85	0.84	0.78	0.75	0.80	0.83	1	13
**17**	1.00	0.99	0.92	0.94	0.92	0.95	0.96	0.96	0.95	0.95	0.95	0.96	12	100	0.98	0.96	0.99	0.99	0.99	0.98	0.97	0.96	8	100
**18**	0.97	0.91	0.99	0.98	0.97	0.97	0.93	0.93	0.94	0.90	0.98	0.97	12	100	0.92	0.85	0.99	0.98	0.99	0.99	0.98	0.96	7	88
**19**	0.83	0.37	0.93	0.91	0.91	0.95	0.92	0.86	0.73	0.77	0.71	0.95	6	50	0.96	0.95	0.94	0.96	0.95	0.91	0.91	0.94	8	100
**20**	0.98	0.98	0.98	0.96	0.97	0.95	0.88	0.90	0.85	0.78	0.94	n.d.	8	67	0.86	0.77	0.79	0.71	n.d.	n.d.	n.d.	n.d.	<4	<50
**21**	0.98	0.99	0.98	0.98	0.98	0.99	0.99	0.99	0.98	0.99	0.97	0.99	12	100	0.99	0.98	0.98	0.98	0.95	0.83	0.95	0.97	7	88
**22**	n.d.	n.d.	0.82	n.d.	0.88	0.79	0.78	0.73	0.71	n.d.	0.77	0.71	<6	<50	1.00	0.99	1.00	1.00	1.00	0.99	0.99	0.99	8	100
**23**	0.96	0.92	n.d.	n.d.	n.d.	n.d.	0.93	n.d.	n.d.	n.d.	n.d.	n.d.	3	25	0.94	0.91	0.96	0.95	0.94	0.94	0.95	0.94	8	100
**24**	0.74	n.d.	n.d.	0.87	0.71	0.72	0.77	0.78	n.d.	n.d.	0.75	n.d.	<6	<50	0.96	0.96	0.97	0.98	0.98	0.97	0.96	0.95	8	100
**25**	n.d.	n.d.	n.d.	n.d.	n.d.	n.d.	n.d.	n.d.	n.d.	n.d.	n.d.	n.d.	<6	<50	0.98	0.98	0.99	0.99	0.99	0.98	0.98	0.98	8	100
**26**	0.95	n.d.	0.97	0.96	0.95	0.97	0.95	0.90	0.87	0.87	0.92	0.90	9	75	0.83	0.85	0.94	0.91	0.89	0.88	0.78	0.91	3	38
**27**	n.d.	n.d.	n.d.	n.d.	n.d.	n.d.	n.d.	n.d.	n.d.	n.d.	n.d.	n.d.	<6	<50	0.94	0.96	0.92	0.98	0.96	0.98	0.93	0.91	8	100
**28**	0.97	0.94	0.94	0.96	0.84	0.85	0.82	0.91	0.74	0.93	n.d.	0.75	6	50	n.d.	n.d.	n.d.	n.d.	n.d.	n.d.	n.d.	n.d.	<4	<50
**29**	0.99	1.00	0.99	0.99	1.00	1.00	1.00	0.99	1.00	0.99	0.99	1.00	12	100	0.99	0.97	1.00	0.99	0.99	0.96	0.97	0.98	8	100
**30**	n.d.	n.d.	n.d.	n.d.	n.d.	n.d.	n.d.	n.d.	n.d.	n.d.	n.d.	n.d.	<6	<50	0.99	0.96	n.d.	0.92	0.90	0.83	0.78	0.80	4	50
**31**	0.99	0.99	0.99	0.99	0.99	0.99	0.99	0.99	0.99	0.99	0.99	0.99	12	100	0.98	0.94	0.95	0.69	0.91	0.79	0.92	0.94	6	75
**32**	0.97	0.91	0.93	0.95	0.95	0.95	0.95	0.92	0.90	0.92	0.92	0.93	12	100	0.98	0.98	0.98	0.98	0.98	0.94	0.97	0.98	8	100
**33**	0.99	0.98	0.98	0.97	0.99	0.99	0.97	0.98	0.98	0.96	0.98	0.99	12	100	0.88	0.88	0.79	n.d.	n.d.	0.50	0.51	n.d.	<4	<50
**34**	0.97	0.99	0.91	0.91	0.97	0.95	0.97	0.99	0.99	0.99	0.99	0.99	12	100	0.99	0.99	1.00	1.00	1.00	1.00	1.00	1.00	8	100
**35**	0.97	0.84	0.93	0.94	0.97	0.94	0.94	0.91	0.97	0.89	0.87	0.97	9	75	0.87	0.85	0.74	n.d.	0.58	n.d.	0.71	0.79	<4	<50
**36**	n.d.	n.d.	0.73	n.d.	n.d.	n.d.	n.d.	0.71	n.d.	n.d.	n.d.	n.d.	<6	<50	0.90	0.86	0.88	0.92	0.87	0.96	0.90	0.93	5	63

**37**	0.74	0.00	0.92	0.93	0.00	0.90	0.75	0.93	0.90	0.96	0.92	0.88	7	58	0.76	n.d.	0.86	0.75	n.d.	0.88	n.d.	n.d.	<4	<50
**38**	n.d.	n.d.	n.d.	n.d.	n.d.	n.d.	n.d.	n.d.	n.d.	n.d.	n.d.	n.d.	<6	<50	0.94	0.96	n.d.	0.95	0.97	0.92	0.94	0.95	7	88
**39**	0.99	0.96	0.96	0.97	0.99	0.96	0.96	0.95	0.99	0.95	0.96	0.98	12	100	0.85	0.80	0.73	0.70	0.68	0.71	0.83	0.90	1	13
**40***	0.90	n.d.	0.91	0.92	0.89	0.90	0.90	n.d.	0.85	0.90	0.92	0.86	7	58	0.86	0.83	0.83	0.83	0.72	0.86	0.82	0.78	<4	<50
**41**	n.d.	n.d.	n.d.	n.d.	n.d.	n.d.	n.d.	n.d.	n.d.	n.d.	n.d.	n.d.	<6	<50	0.98	0.99	0.96	1.00	1.00	0.96	0.94	0.94	8	100
**42**	n.d.	n.d.	n.d.	n.d.	n.d.	n.d.	n.d.	n.d.	n.d.	n.d.	n.d.	n.d.	<6	<50	0.95	0.97	n.d.	0.97	0.97	0.95	0.94	0.90	7	88
**43**	0.91	n.d.	0.91	0.98	0.79	0.94	0.93	0.77	0.85	0.83	0.76	0.90	6	50	0.94	0.94	n.d.	0.71	n.d.	n.d.	0.83	0.75	2	25
**44**	n.d.	n.d.	n.d.	n.d.	n.d.	n.d.	n.d.	n.d.	n.d.	n.d.	n.d.	n.d.	<6	<50	0.87	0.89	0.90	0.87	0.80	0.92	0.93	0.92	4	50
**45***	0.90	0.91	0.94	0.91	0.95	0.91	0.91	0.92	0.75	0.74	n.d.	n.d.	8	67	0.93	0.72	0.95	0.95	0.92	0.54	0.96	0.98	6	75

[1] VOCs are numbered according to Figure 2 and Table 1 in the main manuscript.

* For annotated compounds the calculated spectrum similarity score, based on spectra stored in the NIST/Wiley library, is given.

For compounds identified by authentic standards the overall similarity score (OSS, considering spectral- and RI match) for each file is given.

White fields…The OSS or the spectrum similarity score is ≥ 0.9; Grey fields…The OSS or the spectrum similarity score is ≤ 0.89; n.d.…the compound was not detected.

ID…Number of sample files in which the respective compound was identified or annotated.

ID [%] … Proportion of analyzed samples in which a compound was identified or annotated. Compounds which were identified or annotated in ≥ 50 % of samples are mentioned in Table 1 in the main manuscript.

Table S3. Identified or annotated VOCs in the MGRC of *C. explodens* already reported from ants or other insects in the literature.

ID [1]	Compound	Order	Subfamily	Genus	Gland/Source	Reference
1	Pentan-2-one	Hymenoptera	Myrmicinae	Tetramorium	Mandibular gland	[2]
2	Pentane-2,4-dione	Hymenoptera	Formicinae	Camponotus	Mandibular gland	[3]
3	Hept-5-en-2-one or isomer	Hymenoptera	Apoidea	Trigona	Cephalic gland	[4]
4	Heptan-2-one	Hymenoptera	Formicinae	Colobopsis	Mandibular gland	[5]
5	Benzaldehyde	Hymenoptera	Myrmicinae	Veromessor	Mandibular gland	[6]
6	Trimethylbenzene Isomer 1	Coleoptera [2]	Dermestinae	Dermestes	Abdominal extract	[7]
7	Trimethylbenzene Isomer 2	Coleoptera [2]	Dermestinae	Dermestes	Abdominal extract	[7]
8	n-Decane	Hymenoptera	Formicinae	Camponotus	Dufour’s gland	[8]
9	Hexanoic acid	Hymenoptera	Myrmicinae	Atta	Mandibular gland	[9]
10	Undec-1-ene	Hymenoptera	Formicinae	Paratrechina	Dufour’s gland	[10]
11	2-Methoxyphenol	Orthoptera [2]	Cyrtacanthacridinae	Schistocerca	Gut	[11]
12	n-Undecane	Hymenoptera	Formicinae	Colobopsis	Mandibular gland	[5]
13	3,7-Dimethyloct-6-enal	Hymenoptera	Formicinae	Lasius	Mandibular gland	[12]
14	Benzoic acid	Hymenoptera	Formicinae	Camponotus	Poison gland	[8]
15	Benzene-1,2-diol	Coleoptera [2]	Tenebrioninae	Tribolium	Homogenate extract	[13]
16	n-Dodecane	Hymenoptera	Myrmicinae	Pogonomyrmex	Dufour’s gland	[14]
17	Methyl 2-hydroxybenzoate	Hymenoptera	Formicinae	Myrmecocystus	Mandibular gland	[15]
18	2-Phenylacetic acid	Hymenoptera	Myrmicinae	Atta	Metapleural	[16]
19	Nonanoic acid	Hymenoptera	Formicinae	Camponotus	Mandibular gland	[17]
20	Tridec-1-ene	Hymenoptera	Formicinae	Paratrechina	Dufour’s gland	[10]
21	n-Tridecane	Hymenoptera	Formicinae	Colobopsis	Mandibular gland	[5]
22	1H-indole	Hymenoptera	Myrmicinae	Tetramorium	Venom gland	[18]
23	(E)-3,7-Dimethylocta-2,6-dienoic acid	Hymenoptera	Formicinae	Camponotus	Mandibular gland	[17]
24	5-Methylbenzene-1,3-diol	Hymenoptera	Dolichoderinae	Dolichoderus	Anal gland	[19]
25	3-Acetyl-6-methylpyran-2,4-dione [4]	[3]				
26	1-(2-hydroxy-4,5-dimethylphenyl)ethanone	[3]				
27	4-Hydroxy-6-methylpyran-2-one [4]	[3]				
28	Pentadec-1-ene	Hymenoptera	Ponerinae	Platythyrea	Dufour’s gland	[20]
29	n-Pentadecane	Hymenoptera	Formicinae	Colobopsis	Mandibular gland	[5]
30	Benzene-1,3,5-triol	[3]				
31	n-Hexadecane	Hymenoptera	Formicinae	Camponotus	Dufour’s gland	[8]
32	Heptadec-8-ene	Hymenoptera	Formicinae	Polyrhachis	Mandibular gland	[21]
33	Heptadec-1-ene	Hymenoptera	Myrmicinae	Messor	Poison gland	[22]
34	n-Heptadecane	Hymenoptera	Formicinae	Colobopsis	Mandibular gland	[5]
35	n-Octadecane	Hymenoptera	Ponerinae	Diacamma	Dufour’s gland	[20]
36	Hexadecan-1-al	Hymenoptera	Formicinae	Rossomyrmex	Dufour’s gland	[23]

37	Hexadecan-1-ol	Hymenoptera	Formicinae	Colobopsis	Mandibular gland	[5]
38	1-(2,4,6-Trihydroxyphenyl)ethanone	Hymenoptera	Formicinae	Colobopsis	Mandibular gland	[5]
39	n-Nonadecane	Hymenoptera	Formicinae	Camponotus	Dufour's gland	[8]
40	Heptadecan-2-one or isomer	Hymenoptera	Formicinae	Myrmecocystus	Dufour's gland	[15]
41	5,7-Dihydroxy-2-methylchromen-4-one	Hymenoptera	Formicinae	Colobopsis	Mandibular gland	[5]
42	1-(3-Acetyl-2,4,6-trihydroxyphenyl)ethanone	Coleoptera [2]	Scolytinae	Scolytoplatypus	Symbiotic microorganims	[24]
43	Octadecan-1-ol	Hymenoptera	Formicinae	Colobopsis	Mandibular gland	[5]
44	(Z)-Octadec-9-enoic acid	Hymenoptera	Apinae	Apis	Mandibular gland	[25]
45	Octadecanoic acid	Hymenoptera	Formicinae	Camponotus	Poison gland	[8]

[1] ID…VOCs numbered as shown in Figure 1 and Table 1 in the main manuscript.

[2] To the best of our knowledge, there is no literature available reporting the respective compound from Hymenoptera.

[3] To the best of our knowledge, there is no literature available reporting the respective compound from insects.

Table S4. Data matrix used for statistical analysis of VOCs identified or annotated after liquid-injection-GC-MS.

# [1]	SR [2]	ID [3]	Compound	QI [4] (m/z)	EIC Peak Areas							
					Cexpl_ 5/2014_1	Cexpl_ 5/2014_2	Cexpl_ 4/2015_1	Cexpl_ 4/2015_2	Cexpl_ 4/2015_3	Cexpl_ 11/2015_1	Cexpl_ 11/2015_2	Cexpl_ 11/2015_3
1	1	3	5-Hepten-2-one or isomer	112	2.65E+04	2.37E+04	2.90E+04	5.15E+04	4.33E+04	3.22E+04	2.92E+04	2.99E+04
2	1	4	Heptan-2-one	114	1.77E+07	1.98E+07	2.28E+07	3.23E+07	3.00E+07	1.87E+07	2.02E+07	1.77E+07
3	1	6	Trimethylbenzene isomer 1	120	3.27E+04	8.64E+04	7.86E+04	4.49E+04	5.58E+04	4.38E+04	6.34E+04	5.52E+04
4	1	7	Trimethylbenzene isomer 2	91	8.91E+05	9.09E+05	6.19E+05	9.77E+05	8.60E+05	6.75E+05	7.24E+05	7.02E+05
5	1	8	n-Decane	142	1.42E+06	1.14E+06	6.00E+05	7.59E+05	5.76E+05	2.67E+05	5.37E+05	8.27E+05
6	1	9	Hexanoic acid	60	3.54E+06	3.17E+06	5.57E+06	7.67E+06	5.82E+06	2.14E+06	2.64E+06	2.10E+06
7	1	11	2-Methoxyphenol	109	3.92E+05	4.49E+05	3.72E+05	4.73E+05	4.41E+05	2.54E+05	1.91E+05	1.88E+05
8	1	12	n-Undecane	156	4.96E+07	3.88E+07	1.80E+07	2.26E+07	1.21E+07	6.82E+06	1.61E+07	2.78E+07
9	1	15	Benzene-1,2-diol	110	2.06E+06	2.54E+06	1.93E+06	3.88E+06	2.64E+06	1.42E+03	9.97E+04	0.00E+00
10	1	17	Methyl 2-hydroxybenzoate	152	4.81E+05	5.08E+05	5.85E+05	6.93E+05	6.68E+05	4.58E+05	3.83E+05	3.60E+05
11	1	18	2-Phenylacetic acid	136	1.56E+06	9.70E+05	3.89E+06	4.44E+06	3.28E+06	1.21E+06	1.82E+06	1.36E+06
12	1	19	Nonanoic acid	129	7.39E+05	5.98E+05	1.27E+06	9.39E+05	9.83E+05	3.27E+05	3.99E+05	3.19E+05
13	1	21	n-Tridecane	184	2.72E+06	1.92E+06	1.14E+06	1.41E+06	8.49E+05	5.32E+05	7.90E+05	1.15E+06
14	1	22	1H-indole	117	9.49E+06	7.81E+06	1.12E+07	1.48E+07	1.19E+07	7.22E+06	5.90E+06	4.67E+06
15	1	23	3,7-Dimethylocta-2,6-dienoic acid	100	3.37E+06	2.77E+06	5.48E+06	5.91E+06	5.85E+06	3.35E+06	4.35E+06	3.18E+06
16	1	24	5-Methylbenzene-1,3-diol	124	1.78E+07	1.66E+07	2.41E+07	2.94E+07	2.51E+07	9.16E+06	1.07E+07	8.09E+06
17	1	25	3-Acetyl-6-methylpyran-2,4-dione	153	5.36E+06	4.56E+06	7.31E+06	8.55E+06	8.31E+06	5.16E+06	3.38E+06	2.61E+06
18	10	27	4-Hydroxy-6-methylpyran-2-one	126	8.38E+06	4.63E+06	3.81E+06	3.84E+06	5.04E+06	2.60E+06	1.61E+06	2.13E+06
19	1	29	n-Pentadecane	212	4.98E+06	4.04E+06	3.65E+06	3.65E+06	3.83E+06	1.82E+06	1.97E+06	2.52E+06
20	10	30	Benzene-1,3,5-triol	126	7.32E+06	3.15E+06	2.94E+06	3.95E+06	4.15E+06	5.05E+06	1.51E+06	5.11E+05
21	1	31	n-Hexadecane	226	4.21E+06	2.57E+06	1.66E+06	3.77E+06	1.53E+06	1.35E+06	2.02E+06	1.78E+06
22	1	32	Heptadec-8-ene	238	1.62E+06	1.63E+06	2.57E+06	2.63E+06	2.33E+06	1.42E+06	1.60E+06	1.20E+06
23	1	34	n-Heptadecane	240	3.70E+07	2.31E+07	1.54E+07	1.59E+07	1.23E+07	6.43E+06	1.09E+07	1.16E+07
24	1	36	Hexadecan-1-al	96	5.65E+05	5.03E+05	8.97E+05	9.51E+05	8.50E+05	1.44E+06	7.82E+05	9.26E+05
25	60	38	1-(2,4,6-Trihydroxyphenyl)ethanone	168	2.93E+07	2.64E+07	2.90E+07	3.37E+07	3.02E+07	2.85E+07	1.16E+07	1.80E+07
26	60	41	5,7-Dihydroxy-2-methylchromen-4-one	192	1.21E+07	1.19E+07	1.55E+07	1.59E+07	1.41E+07	1.55E+07	8.25E+06	9.49E+06
27	10	42	1-(3-Acetyl-2,4,6-trihydroxyphenyl)ethanone	210	4.36E+06	3.02E+06	4.37E+06	4.97E+06	5.71E+06	4.72E+06	1.81E+06	2.52E+06
28	1	44	(Z)-Octadec-9-enoic acid	264	3.97E+05	4.27E+05	1.24E+06	4.74E+05	2.85E+05	4.98E+05	7.10E+05	3.92E+05
29	1	45	Octadecanoic acid	284	1.07E+06	2.45E+06	1.32E+06	1.02E+06	5.56E+05	6.32E+05	1.31E+06	2.39E+06

EIC peak areas were obtained with the batch quantification function of MetaboliteDetector.

[1] Sorted by order of appearance in the chromatogram (*i.e.* by RI, see Figure 1 and Table 1 in main manuscript).

[2] SR = split ratio used in GC analysis to obtain narrow peak shapes, see also Figure 2 in main manuscript.

[3] IDs given according to Table 1 in the main manuscript.

[1] QI = quantification ion used for EIC peak area determination by MetaboliteDetector.

References

1. Laciny, A.; Zettel, H.; Kopchinskiy, A.; Pretzer, C.; Pal, A.; Salim, K.A.; Rahimi, M.J.; Hoenigsberger, M.; Lim, L.; Jaitrong, W., et al. Colobopsis explodens sp. n., model species for studies on "exploding ants"(Hymenoptera, Formicidae), with biological notes and first illustrations of males of the Colobopsis cylindrica group. *ZooKeys* **2018**, 751, 751-740.
2. Ali, M.F.; Cammaerts, R.; Evershed, R.P.; Morgan, E.D. A re-examination of the mandibular gland contents of Tetramorium caespitum (Hymenoptera: Formicidae). *Insect biochemistry* **1987**, *17*, 237-241.
3. Brand, J.; Mabinya, L.; Morgan, E. Volatile chemicals in glands of the carpenter ant, Camponotus arminius. *African Zoology* **1999**, *34*, 140-142.
4. Bian, Z.; Fales, H.; Blum, M.; Jones, T.; Rinderer, T.; Howard, D. Chemistry of cephalic secretion of fire beeTrigona (Oxytrigona) tataira. *Journal of chemical ecology* **1984**, *10*, 451-461.
5. Sakolrak, B.; Blatrix, R.; Sangwanit, U.; Arnamnart, N.; Noisripoom, W.; Thanakitpipattana, D.; Buatois, B.; Hossaert-McKey, M.; Kobmoo, N. Ant-produced chemicals are not responsible for the specificity of their Ophiocordyceps fungal pathogens. *Fungal Ecology* **2018**, *32*, 80-86.
6. Blum, M.S.; Padovani, F.; Curley, A.; Hawk, R.E. Benzaldehyde: defensive secretion of a harvester ant. *Comparative biochemistry and physiology* **1969**, *29*, 461-465.
7. Korada, R.R.; Griepink, F.C. Aggregation pheromone compounds of the black larder beetle Dermestes haemorrhoidalis Kuster (Coleoptera: Dermestidae). *Chemoecology* **2009**, *19*, 177-184.
8. Kohl, E.; Hölldobler, B.; Bestmann, H. Trail and recruitment pheromones in Camponotus socius (Hymenoptera: Formicidae). *Chemoecology* **2001**, *11*, 67-73.
9. de Lima Mendonça, A.; Da Silva, C.E.; de Mesquita, F.L.T.; da Silva Campos, R.; Do Nascimento, R.R.; de Azevedo Ximenes, E.C.P.; Sant'Ana, A.E.G. Antimicrobial activities of components of the glandular secretions of leaf cutting ants of the genus Atta. *Antonie Van Leeuwenhoek* **2009**, *95*, 295-303.
10. Witte, V.; Abrell, L.; Attygalle, A.B.; Wu, X.; Meinwald, J. Structure and function of Dufour gland pheromones from the crazy ant Paratrechina longicornis. *Chemoecology* **2007**, *17*, 63-69.
11. Dillon, R.J.; Vennard, C.T.; Charnley, A.K. Pheromones: exploitation of gut bacteria in the locust. *Nature* **2000**, *403*, 851-851.
12. Blum, M.S.; Padovani, F.; Hermann JR, H.R.; Kannowski, P.B. Chemical releasers of social behavior. XI. Terpenes in the mandibular glands of Lasius umbratus. *Annals of the Entomological Society of America* **1968**, *61*, 1354-1359.
13. Unruh, L.M.; Xu, R.; Kramer, K.J. Benzoquinone levels as a function of age and gender of the red flour beetle, Tribolium castaneum. *Insect Biochemistry and Molecular Biology* **1998**, *28*, 969-977.
14. Regnier, F.E.; Nieh, M.; Hölldobler, B. The volatile Dufour's gland components of the harvester ants Pogonomyrmex rugosus and P. barbatus. *Journal of Insect Physiology* **1973**, *19*, 981-992.
15. Lloyd, H.; Blum, M.; Snelling, R.; Evans, S. Chemistry of mandibular and Dufour's gland secretions of ants in genusMyrmecocystus. *Journal of chemical ecology* **1989**, *15*, 2589-2599.
16. Do Nascimento, R.R.; Schoeters, E.; Morgan, E.D.; Billen, J.; Stradling, D.J. Chemistry of metapleural gland secretions of three attine ants, Atta sexdens rubropilosa, Atta cephalotes, andAcromyrmex octospinosus (Hymenoptera: Formicidae). *Journal of chemical ecology* **1996**, *22*, 987-1000.
17. Lloyd, H.; Blum, M.; Duffield, R. Chemistry of the male mandibular gland secretion of the ant, Camponotus clarithorax. *Insect Biochemistry* **1975**, *5*, 489-494.
18. Jackson, B.; Keegans, S.; Morgan, E.; Cammaerts, M.-C.; Cammaerts, R. Trail pheromone of the antTetramorium meridionale. *The Science of Nature* **1990**, *77*, 294-296.

19. Blum, M.S.; Jones, T.H.; Snelling, R.R.; Overal, W.L.; Fales, H.M.; Highet, R.J. Systematic implications of the exocrine chemistry of some Hypoclinea species. *Biochemical Systematics and Ecology* **1982**, *10*, 91-94.
20. Morgan, E.D.; Jungnickel, H.; Keegans, S.J.; Do Nascimento, R.R.; Billen, J.; Gobin, B.; Ito, F. Comparative survey of abdominal gland secretions of the ant subfamily Ponerinae. *Journal of chemical ecology* **2003**, *29*, 95-114.
21. Bellas, T.; Hölldobler, B. Constituents of mandibular and Dufour's glands of an AustralianPolyrhachis weaver ant. *Journal of chemical ecology* **1985**, *11*, 525-538.
22. Co, J.E.; Jones, T.H.; Hefetz, A.; Tinaut, A.; Snelling, R.R. The comparative exocrine chemistry of nine Old World species of Messor (Formicidae: Myrmicinae). *Biochemical systematics and ecology* **2003**, *31*, 367-373.
23. Ruano, F.; Hefetz, A.; Lenoir, A.; Francke, W.; Tinaut, A. Dufour's gland secretion as a repellent used during usurpation by the slave-maker ant Rossomyrmex minuchae. *Journal of Insect Physiology* **2005**, *51*, 1158-1164.
24. Kenny, P.T.; Tamura, S.Y.; Fredenhagen, A.; Naya, Y.; Nakanishi, K.; Nishiyama, K.; Sugiura, M.; Kita, H.; Komura, H. Symbiotic micro-organisms of insects: A potential new source for biologically active substances. *Pesticide science* **1989**, *27*, 117-131.
25. ENGELS, W.; ROSENKRANZ, P.; ADLER, A.; TAGHIZADEH, T.; LÜBKE, G.; FRANCKE, W. Mandibular gland volatiles and their ontogenetic patterns in queen honey bees, Apis mellifera carnica. *Journal of Insect Physiology* **1997**, *43*, 307-313.
26. Bacardit, R.; Moreno-Mañas, M. Synthesis of δ-lactonic pheromones ofXylocopa hirsutissima andVespa orientalis and an allomone of some ants of genusCamponotus. *Journal of chemical ecology* **1983**, *9*, 703-714.
